# Insights into the Competing Mechanisms and Origin of Enantioselectivity for N-Heterocyclic Carbene-Catalyzed Reaction of Aldehyde with Enamide

**DOI:** 10.1038/srep38200

**Published:** 2016-12-01

**Authors:** Yan Qiao, Xinhuan Chen, Donghui Wei, Junbiao Chang

**Affiliations:** 1 Department of Pathophysiology, School of Basic Medical Sciences; 2 Henan Provincial Cooperative Innovation Center for Cancer Chemoprevention, Zhengzhou 450001, Henan, China; 3 The College of Chemistry and Molecular Engineering, Zhengzhou University, Zhengzhou 450001, Henan, China

## Abstract

Hydroacylation reactions and aza-benzoin reactions have attracted considerable attention from experimental chemists. Recently, Wang *et al*. reported an interesting reaction of N-heterocyclic carbene (NHC)-catalyzed addition of aldehyde to enamide, in which both hydroacylation and aza-benzoin reactions may be involved. Thus, understanding the competing relationship between them is of great interest. Now, density functional theory (DFT) investigation was performed to elucidate this issue. Our results reveal that enamide can tautomerize to its imine isomer with the assistance of HCO_3_^−^. The addition of NHC to aldehydes formed Breslow intermediate, which can go through cross-coupling with enamide via hydroacylation reaction or its imine isomer via aza-benzoin reaction. The aza-benzoin reaction requires relatively lower free energy barrier than the hydroacylation reaction. The more polar characteristic of C=N group in the imine isomers, and the more advantageous stereoelectronic effect in the carbon-carbon bond forming transition states in aza-benzoin pathway were identified to determine that the imine isomer can react with the Breslow intermediate more easily. Furthermore, the origin of enantioselectivities for the reaction was explored and reasonably explained by structural analyses on key transition states. The work should provide valuable insights for rational design of switchable NHC-catalyzed hydroacylation and aza-benzoin reactions with high stereoselectivity.

As powerful organocatalysts, N-heterocyclic carbenes (NHCs) have been widely used in various carbon‒carbon and carbon‒heteroatom bonds forming reactions^1^,^2^. An attractive feature for NHCs is that they can catalyze the polarity reversal of various carbonyl compounds and generate acyl anion equivalents, which provide an elegant access to a wide range of organic transformations^3^,^4^. Among them, the NHC-catalyzed benzoin condensation and Stetter reactions are the two most exploited reactions as a result of the success of these reactions in various useful transformations^1^. Noteworthy, almost all the previously reported Stetter reactions catalyzed by NHC involve electron-withdrawing olefins, the use of electron-rich olefins in NHC-catalyzed cross-coupling reactions is still very challenging. Until recently, Wang *et al*. reported the first example of NHC-catalyzed highly enantioselective intermolecular addition of aldehydes to electron-rich olefins, i.e. enamides, to synthesize valuable N-acyl-protected amine derivatives (Fig. 1)^ ^5. As shown in Fig. 1, the reactions are proposed to be initiated by the nucleophilic attack of NHC to the aldehyde substrates yielding the well-known Breslow intermediate, which can then go through cross-coupling with substrate enamides and result into the final products. Interestingly, both hydroacylation and aza-benzoin reaction mechanisms may be involved in the special reaction, and some important mechanistic questions need to be answered. For example: (1) Substrate enamide **2** can tautomerize to its imine isomer **2′**. However, how the tautomerization happen remains elusive. (2) The Breslow intermediate can react with **2** via hydroacylation pathway, while it can also react with **2′** via aza-benzoin reaction pathway. Thus, it is very difficult to discern which one is the actual reactant and which competing reaction occurs preferentially. (3) The reaction is highly enantioselective, and it is meaningful to explore the origin of the stereoselectivity and identify the factors that control the stereoselectivity. Due to the difficulty on the structural detection of the intermediates and transition states involved in the rate- and stereoselectivity-determining steps in the experiment, it is highly desirable to perform a theoretical study to obtain the general principle for this kind of organocatalytic reactions.

In recent years, NHC-catalyzed reactions have attracted much attention from theoretical chemists^6^,^7^,^8^,^9^,^10^,^11^,^12^,^13^. Particularly, the NHC-catalyzed Stetter reactions^7^,^10^,^11^,^12^,^13^, and the NHC-catalyzed aldehyde-aldehyde and aldehyde-ketone cross-benzoin reactions^14^,^15^,^16^,^17^ have been theoretically investigated, respectively. However, the hydroacylation mechanism involved in the reaction shown in Fig. 1 has been demonstrated to be different from the Stetter reactions^8^,^18^, and the aldehyde-imine aza-benzoin reactions catalyzed by NHC have never been studied in theory, not to mention the competing relationship between them. Besides, our interest in NHC-catalyzed reactions also prompts us to investigate the origin of selectivities for the competing reactions in detail^19^,^20^,^21^,^22^,^23^,^24^,^25^,^26^,^27^,^28^,^29^,^30^,^31^. In the present study, the commonly used DFT theoretical investigation^32^,^33^,^34^,^35^,^36^,^37^,^38^,^39^,^40^,^41^,^42^ on the reaction between R1 (in which Ar_1_ = para chlorphenyl) and R2 (in which Ar_2_ = phenyl) catalyzed by NHC depicted in Fig. 1, was pursued in order to shed light on details of each elementary step at the molecular level and to reach more comprehensive understanding to the enantioselectivity of this interesting reaction.

## Results

**Figure 1:**
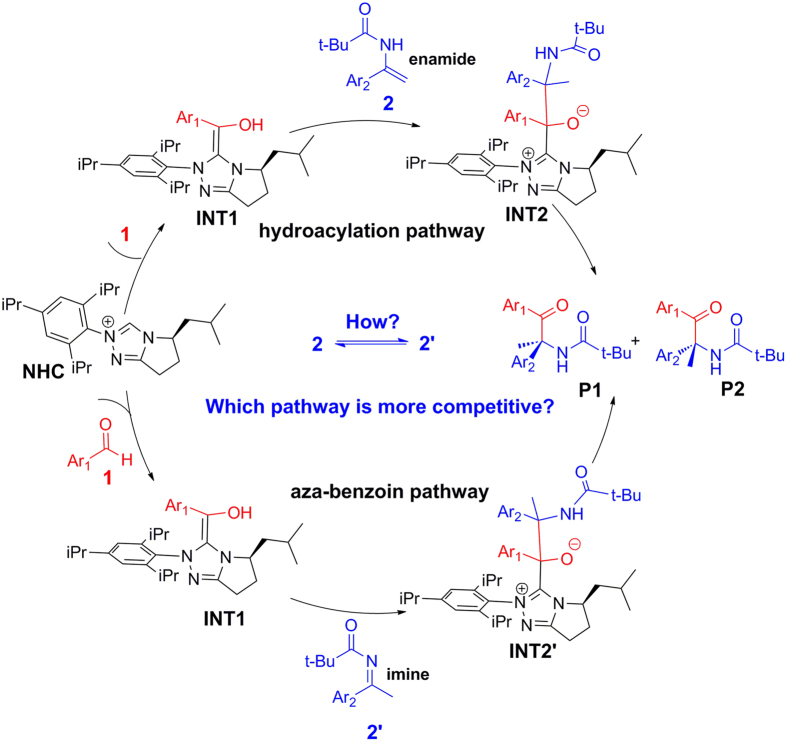
The possible competing mechanisms for NHC-catalyzed reactions between benzaldehyde and enamide **2** (or its isomer **2′**).

Our calculated results confirmed that NHC indeed initiate the reaction by nucleophilic attack to the aldehyde substrate yielding the well-known Breslow intermediate, which can then go through a cross-coupling with the other substrate via two competing pathways depicted in Fig. 1 to form the same final products. In the following sections, we will discuss the detailed reaction processes, including the formation of Breslow intermediate, the tautomerization between enamide and the corresponding imine isomer, hydroacylation reactions between Breslow intermediate and enamide, aza-benzoin reaction between Breslow intermediate and imine, the competition of hydroacylation reaction and aza-benzoin reaction, and origin of the enantioselectivity.

### Formation of Breslow intermediate

**Figure 2:**
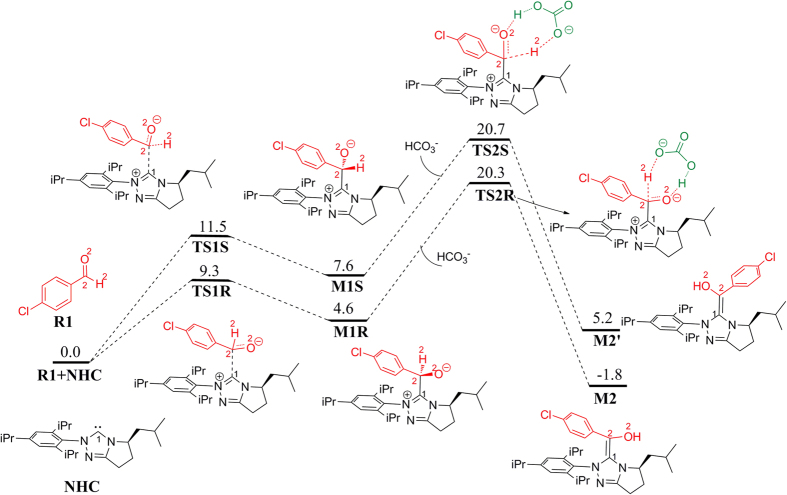
Free energy profile for the formation of Breslow intermediates.

As shown in Fig. 2, the reaction is initiated by the nucleophilic addition of NHC to 4-chlorobenzaldehyde **R1**. Noteworthy, **R1** has a prechiral center, i.e. C2 atom. Attack from NHC on the *Si* or *Re* face of **R1** can respectively lead to two stereoselective intermediate **M1R** or **M1S**, in which the **R**/**S** represents the chirality of C2 atom. The two kinds of stereochemically distinct attack modes have different free energy barriers. According to our calculations, the *Si* face attack requires the free energy barrier of 9.3 kcal/mol, which is 2.3 kcal/mol lower than that of the *Re* face attack (ΔG = 11.5 kcal/mol). The produced intermediates **M1R** and **M1S** are zwitterions. In the following, zwitterionic intermediates **M1R** and **M1S** would go through 1,2-proton transfer to afford the Breslow intermediates **M2/M2′**, in which C2 has no chirality. Direct 1, 2-proton transfer is impossible because of the strong strain in the three-membered ring transition state. Protic solvents in the reaction system have been demonstrated many times to be able to mediate the proton transfer process in the formation of Breslow intermediates as well as in many other proton transfer processes^11^,^20^,^25^. Herein, we proposed that the small amount of HCO_3_^−^ in the reaction system can mediate the proton transfer process. The corresponding transition state **TS2R** was located 0.4 kcal/mol lower in free energy than **TS2S**. The newly formed Breslow intermediate **M2** produced from **TS2R** is 7.0 kcal/mol more stable than **M2′** generated via **TS2S**. Structural analyses (Supplementary Fig. S1) reveal that the advantageous π‒π interaction, C‒H•••π interaction, C‒H•••Cl and C‒H•••O hydrogen bond interactions determined that **M2** is more stable than **M2′**. From the free energy profile shown in Fig. 2, it can be concluded that the reaction pathway affording Breslow intermediate **M2** is more likely to occur. Therefore, in the following reaction steps, we only considered the reaction processes associated with **M2**.

### The tautomerization between enamide and imine

**Figure 3:**
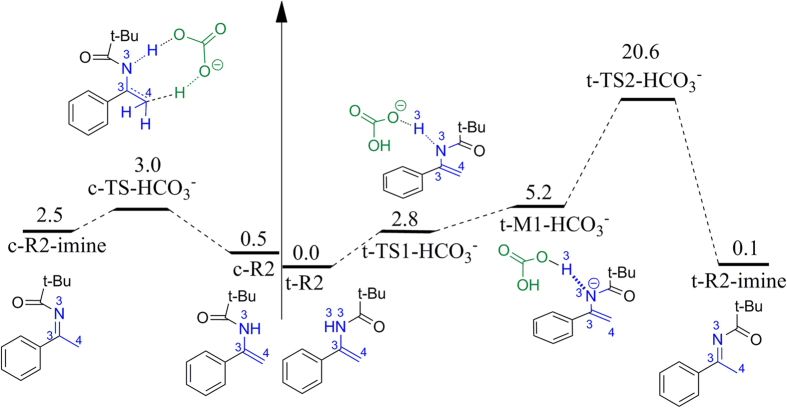
Free energy profile for tautomerization between enamide and its imine isomer.

The reactant enamide **R2** can tautomerize to its imine isomer. In the imine configuration, we selected C3=N3 bond as a reference standard. As shown in Fig. 3, if the acyl group and phenyl group are in *trans* configuration, we denote it as ***t*****-R2-imine**, while in *cis* configuration, we denote it as ***c*****-R2-imine**. Correspondingly, ***t*****-R2-imine** can tautomerize to ***t*****-R2**, and ***c-*****R2-imine** can tautomerize to ***c*****-R2**. In total, there are four distinctive conformations for **R2**. In the following, we considered the tautomerization between ***t*****-R2** and ***t*****-R2-imine**, and between ***c*****-R2** and ***c*****-R2-imine**.

Firstly, we considered the bimolecular reaction mechanisms for the tautomerization between ***t*****-R2** and ***t*****-R2-imine**. The bimolecular reaction consists of two reaction processes with the highest activation free energy barrier of 46.4 kcal/mol (Supplementary Fig. S2), which is so high that it is unlikely for the reaction to happen. In addition, we also considered HCO_3_^−^-mediated tautomerization mechanism between them. According to the calculated results, HCO_3_^−^ would first abstract the hydrogen from ‒NH group *via* transition state ***t*****-TS1-HCO**_**3**_^**−**^. The free energy barrier for this step was calculated to be 2.8 kcal/mol. The formed intermediate ***t*****-M1-HCO**_**3**_^**−**^ is 0.3 kcal/mol lower in energy than ***t*****-TS1-HCO**_**3**_^**−**^. However, by addition of the thermal correction, ***t*****-M1-HCO**_**3**_^**−**^ is 2.6 kcal/mol higher in free energy than ***t*****-TS1-HCO**_**3**_^**−**^. Followed by the formation of ***t*****-M1-HCO**_**3**_^**−**^ is the hydrogen transfer from **H**_**2**_**CO**_**3**_ to the terminal alkene carbon C4 *via* transition state ***t*****-TS2-HCO**_**3**_^**−**^ resulting into ***t*****-R2-imine**. This reaction step requires a free energy barrier of 20.6 kcal/mol, which is significantly lower than that of the bimolecular fashion.

For the *cis* configuration, we also considered the bimolecular and HCO_3_^−^-mediated mechanisms. The bimolecular fashion is also a two-step process with activation free energy barrier as high as 48.6 kcal/mol (Supplementary Fig. S3), while the HCO_3_^−^-mediated mechanism is a concerted process with activation free energy barrier of only 2.5 kcal/mol. The produced isomer ***c*****-R2-imine** is 2.0 kcal/mol unstable than ***c*****-R2**, and the free energy barrier for the reverse reaction is only 0.5 kcal/mol, indicating that this reaction process is highly reversible. Given this reason, we did not take ***c*****-R2-imine** into consideration in the following reaction processes.

As described on the above, the C=C group in enamides would undergo hydroacylation reactions with the Breslow intermediate, while the C=N group in imines would undergo cross-aza-benzoin reactions with the Breslow intermediate. Based on this, ***t*****-R2** and ***c*****-R2** would undergo hydroacylation reactions, while***t*****-R2-imine** would go through cross-aza-benzoin reactions. In the following, we will discuss these competing reaction pathways in detail.

### Hydroacylation reactions between Breslow intermediate and enamide

**Figure 4:**
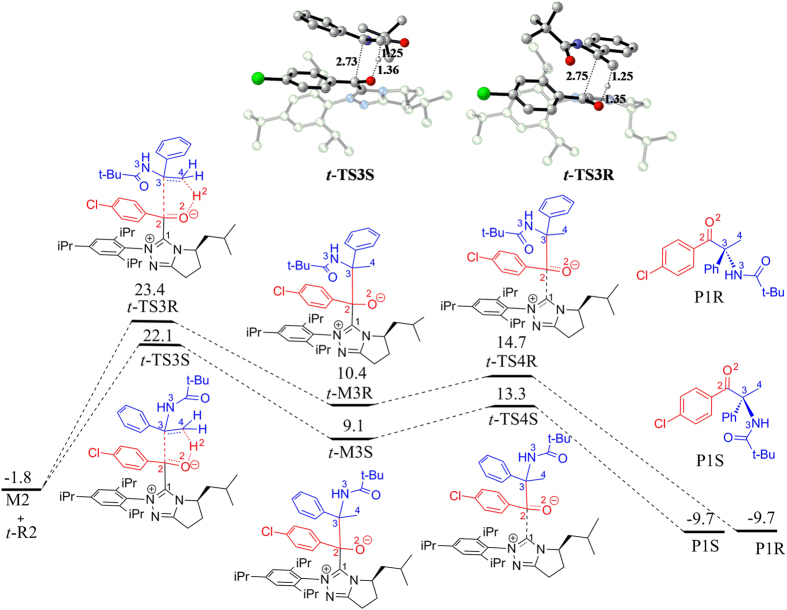
Free energy profile for the hydroacylation reaction between M2 and *t*-R2.

Firstly, we take ***t*****-R2** into consideration for the hydroacylation reaction with **M2**. The negatively charged carbon atom C2 in Breslow intermediate **M2** would nucleophilic attack on the prechiral center C3 atom of ***t*****-R2**. Attack to the *Re*-face of ***t*****-R2** via transition state ***t*****-TS3S** can lead to***t*****-M3S**, in which the “S” represents *S* configuration of C3 atom, while attack to the *Si*-face of ***t*****-R2** via transition state ***t*****-TS3R** can lead to ***t*****-M3R**, in which C3 is in *R* configuration. As shown in Fig. 4, the free energy barrier for ***t*****-TS3S** with respect to **M2** + ***t-*****R2** amounts to 23.9 kcal/mol, which is 1.3 kcal/mol lower than that of ***t*****-TS3R** (ΔG = 25.2 kcal/mol). Noteworthy, the nucleophilic attack is accompanied by the proton transfer from O2 to C4 atom. In the newly formed intermediates ***t*****-M3S** and ***t*****-M3R**, C2‒C3 bond is formed and H2 atom is transferred to C4 atom. The bond distances for C2‒C3, O2‒H2, and H2‒C4 in transition states ***t*****-TS3S/*****t*****-TS3R** are 2.73/2.75, 1.36/1.35 and 1.25/1.25 Å, respectively, indicating that proton migration occurs prior to carbon-carbon bond formation. This observation is very different from the previous theoretical study on Stetter reactions^7^. Subsequently, the elimination of NHC from ***t*****-M3S** and ***t*****-M3R** *via* transition states ***t*****-TS4S** and ***t*****-TS4R** can produce the final products **P1S** and **P1R**, respectively. According to the calculated results, the free energy barriers for this single step are 4.2 and 4.3 kcal/mol for the *S* and *R* configurations, respectively. According to the free energy profile shown in Fig. 4, the reaction process leading to product **P1S** is more energetically favorable than the process leading to **P1R**, which is in agreement with the experimentally observed results that **P1S** is the major product.

In addition to***t*****-R2**, we also considered***c-*****R2** as a reactant of hydroacylation reaction. There are also two possible stereoselective nucleophilic attack modes, which is corresponding to the attack from the Breslow intermediate **M2** on the *Re/Si*-face of ***c*****-R2.** The corresponding free energies for the carbon‒carbon bond forming transition states ***c-*****TS3S** and ***c-*****TS3R** with respect to **M2** + ***t-*****R2** are 24.8 and 27.6 kcal/mol (Supplementary Fig. S4), respectively, which are higher than those of ***t-*****TS3S** (ΔG = 23.9 kcal/mol) and ***t-*****TS3R** (ΔG = 25.2 kcal/mol). Therefore, it is easier for ***t*****-R2** to react with the Breslow intermediate **M2** in relative to ***c*****-R2**. Likewise, proton transfer and carbon-carbon bond formation also take place in the concerted manner although proton transfer occurs first. Finally, the elimination of NHC can produce the final products **P1S** and **P1R**, respectively. Interestingly, the reaction process for *S* configuration also requires lower activation free energies than the *R* configuration.

### Aza-benzoin reaction between Breslow intermediate and imine

**Figure 5:**
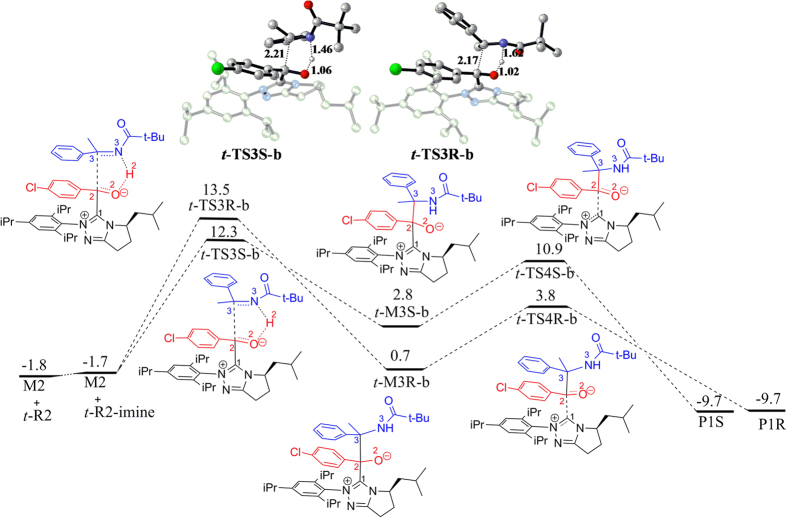
Free energy profile for the cross-aza-benzoin reaction between M2 and *t*-R2-imine.

As described above, ***t*****-R2** can tautomerize to its imine isomer ***t*****-R2-imine**, which then reacts with Breslow intermediate *via* aza-benzoin condensation. During this reaction process, C2 atom in Breslow intermediate **M2** would nucleophilic attack on C3 atom of ***t*****-R2-imine**. Attack to the *Re*-face of ***t*****-R2-imine** *via* transition state ***t*****-TS3S-b** can lead to***t*****-M3S-b**, in which C3 is in *S* configuration. Attack to the *Si*-face of ***t*****-R2** *via* transition state ***t*****-TS3R** can lead to ***t*****-M3R**, in which C3 is in *R* configuration. In contrast to the hydroacylation reaction for which the terminal alkene carbon abstracts the proton from the hydroxyl group, it is the nitrogen atom in the C=N group that abstracts the proton in the aza-benzoin reaction. As shown in Fig. 5, the free energy barriers for ***t*****-TS3S-b** and ***t*****-TS3R-b** with respect to **M2** + ***t-*****R2** amount to 14.1 and 15.3 kcal/mol, which are significantly lower than those of the hydroacylation reactions. Noteworthy, the nucleophilic attack is accompanied by proton transfer from O2 to N3 atom instead of C4 atom. The bond distances for C2‒C3, N2‒H2, and H2‒O2 in transition state ***t*****-TS3S-b/*****t*****-TS3R-b** (shown in Fig. 5) are 2.21/2.17, 1.46/1.62 and 1.06/1.02 Å, respectively, indicating that carbon-carbon bond formation is more advanced than the proton migration. This observation is remarkably different from the hydroacylation reactions described on the above but similar with the previous reported aldehyde-aldehyde benzoin reactions^14^. In the formed intermediates ***t*****-M3S-b** and ***t*****-M3R-b**, C2‒C3 bond is formed and H2 atom is transferred to N3. Subsequently, the elimination of NHC from ***t*****-M3S-b** and ***t*****-M3R-b** via transition states ***t*****-TS4S-b** and ***t*****-TS4R-b** can produce the final products **P1S** and **P1R**, respectively. According to the free energy profile, ***t*****-TS3S-b** is 1.2 kcal/mol lower than that of ***t*****-TS3R-b**, indicating that the reaction process leading to product **P1S** is also more energetically favorable than the process leading to **P1R**.

#### Pathway Comparison

**Figure 6:**
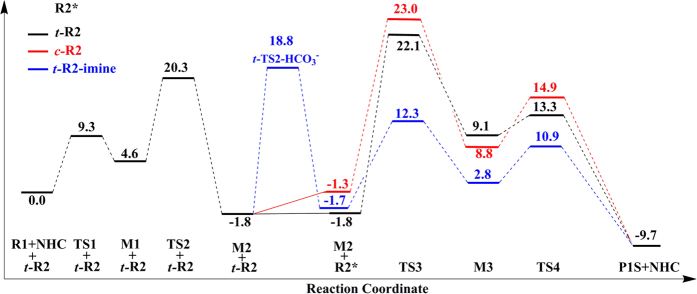
An overview of the reaction coordinates for the pathways leading to P1S.

By now, the reaction pathways for the three possible reactants ***t-*****R2**, ***c*****-R2**, and ***t*****-R2-imine** leading to two enantioselective products have been discussed in detail. An overlay of each reaction pathway leading to the preferred product **P1S** is depicted in Fig. 6. Nucleophilic attack of NHC to the aryl aldehyde **R1** followed by a proton transfer forms Breslow intermediate **M2**, which can then go through cross-coupling with ***t*****-R2** and ***c-*****R2** via hydroacylation reactions. As described on the above, the *trans* configuration of enamide, i.e. ***t*****-R2**, can cross transition state ***t-*****TS2-HCO**_**3**_^**−**^ with a potentially high barrier (ΔG = 20.6 kcal/mol) to tautomerize to its imine isomer ***t-*****R2-imine**, which can also react with the Breslow intermediate **M2** via aza-benzoin reaction. As shown in Fig. 6, for the hydroacylation reactions involving ***t-*****R2** and ***c-*****R2,** the carbon-carbon bond formation step has the highest free energy barrier and thus is rate-determining, while for the aza-benzoin reaction pathway involving ***t-*****R2-imine**, the tautomerization step is rate-determining. Obviously, the aza-benzoin reaction pathways involving ***t*****-R2-imine** have relative lower free energy barrier with respect to the hydroacylation reactions involving ***t-*****R2** and ***c*****-R2**, indicating that aza-benzoin reaction pathway is very competitive.

**Figure 7:**
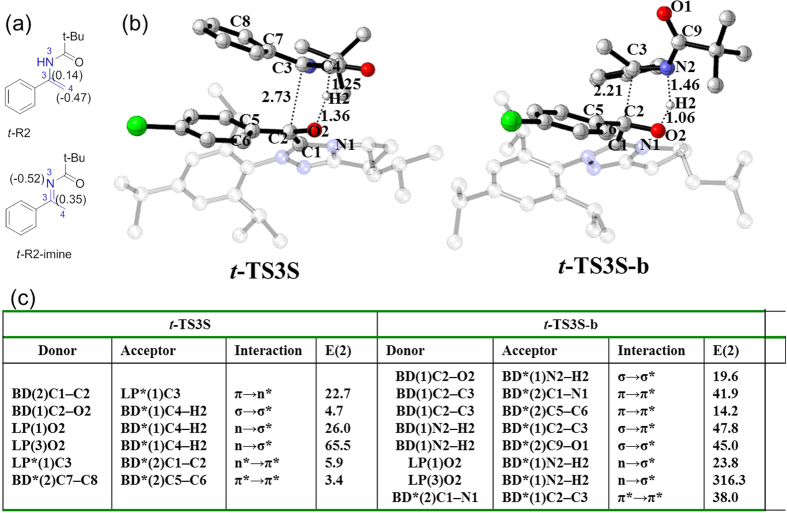
(**a**) the NBO charges for **t-R2** and **t-R2-imine**; (**b**) the transition state structures of **t-TS3S** and **t-TS3S-b**; (**c**) the Second-Perturbation Energies *E*(2) (kcal/mol) of Donor-Acceptor Interactions with Respect to ***t*****-TS3S** and ***t-*****TS3S-b**.

To identify why the carbon-carbon bond formation transition state in aza-benzoin reaction has lower free energy barrier relative to the hydroacylation reactions, we first performed NBO charge analyses on ***t*****-R2** and ***t*****-R2-imine**. As shown in Fig. 7a, the NBO charges on C2 of ***t*****-R2** and ***t*****-R2-imine** are 0.14 e and 0.35 e, respectively, indicating that the electrophilicity of C2 in ***t*****-R2-imine** is stronger than that in ***t*****-R2**, which facilitates the nucleophilic attack of Breslow intermediate **M2** on C2 atom of ***t*****-R2-imine**. In addition, N2 atom of ***t*****-R2-imine** is also more negative than C3 atom of ***t*****-R2**, and the more negative charge on N2 relative to C3 atom should facilitate the proton transfer from the hydroxyl group of Breslow intermediate to N2 atom of ***t*****-R2-imine**.

In addition to the NBO charge analysis on the reactants, we also compared the structures of two representative key transition states ***t*****-TS3S** and ***t*****-TS3S-b**. As indicated on the above, the carbon-carbon bond formation and proton transfer take place through a concerted but asynchronous mechanism in both hydroacylation reaction and aza-benzoin reaction. However, the sequence of the two events is very different. In the hydroacylation reactions, proton migration occurs prior to carbon-carbon bond formation while the sequence is reversed in aza-benzoin reactions. By comparison, the forming C2‒C3 bond in ***t*****-TS3S** and ***t*****-TS3S-b** is 2.73 and 2.21 Å (shown in Fig. 7b), respectively. The shorter C2‒C3 bond distance in ***t*****-TS3S-b** reveals that the interaction between the two fragments of **M2** and ***t*****-R2-imine** in ***t*****-TS3S-b** should be stronger than that in ***t*****-TS3S**. In order to verify this speculation, we also analyzed the second-order perturbative donor-acceptor interactions^43^ for the two transition states. Listed in Fig. 7c is the second-order perturbation energy *E*(2) (kcal/mol) in transition states ***t*****-TS3S** and ***t*****-TS3S-b**. As shown, the main stabilization donor-acceptor interactions in transition state ***t*****-TS3S** come from the charge transfer from the lone pair orbital of O2 atom to the σ* orbital of the forming C4‒H2 bond (E_n→σ*_ = 65.5 kcal/mol and E_n→σ*_ = 26.0 kcal/mol) and the charge transfer from the π orbital of C1‒C2 bond to the vacant lone pair orbital of C3 atom (E_π→n*_ = 22.7 kcal/mol). By comparison, the main stabilization interactions in ***t*****-TS3S-b** come from the charge transfer from the lone pair orbital of O2 atom to the σ* orbital of the forming N2‒H2 bond with E_n→σ*_ = 316.3 kcal/mol and E_n→σ*_ = 23.8 kcal/mol. Besides, there are some other significant stabilization interactions in ***t*****-TS3S-b**, like the interaction between N2‒H2 σ orbital and the C2‒C3 π* orbital with E_σ→π*_ = 47.8 kcal/mol, and the interaction between N2‒H2 σ orbital and C9‒O1 σ* orbital with E_σ→σ*_ = 45.0 kcal/mol as well as the interaction between C1‒N1 π* orbital and the C2‒C3 π* orbital with E_π→π*_ = 38.0 kcal/mol. Taken together, the stereoelectronic effect in ***t*****-TS3S-b** is more favorable than that in ***t*****-TS3S**.

To sum up, the high polar characteristic of C2=N2 group in ***t*****-R2-imine** and the more advantageous stereoelectronic effect in ***t*****-TS3S-b** render the reaction of ***t*****-R2-imine** with Breslow intermediate **M2** more easily.

### Origin of the enantioselectivity

**Figure 8:**
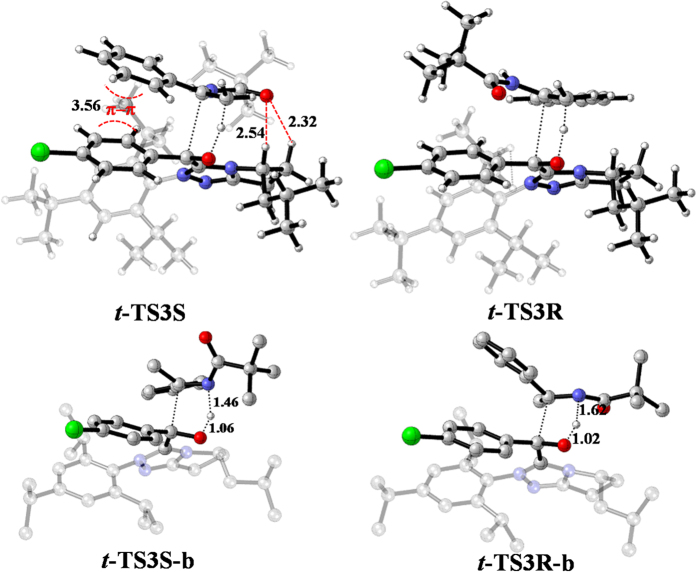
The enantioselective transition state structures of ***t*****-TS3S**, ***t*****-TS3R**, ***t*****-TS3S-b** and ***t*****-TS3R-b**.

For both the hydroacylation reactions and the aza-benzoin reactions, the *S*-configured product is preferentially produced. To explain the observed enantioselectivity, we compared the structures of the enantioselective transition states (shown in Fig. 8). For ***t*****-TS3S** and ***t*****-TS3R**, the C‒H•••O hydrogen bond interaction and π‒π interaction present in ***t*****-TS3S** (colored in red) are absent in ***t*****-TS3R**, which determined that ***t*****-TS3S** has lower free energy relative to ***t*****-TS3R**. For ***t*****-TS3S-b** and ***t*****-TS3R-b**, the N2•••H2 distance in ***t*****-TS3S-b** is shorter than that in ***t*****-TS3R-b**, demonstrating that N•••H‒O hydrogen bond interaction in ***t*****-TS3S-b** is stronger than that in***t*****-TS3R-b.** Therefore, it is the stronger N•••H‒O hydrogen interaction in ***t*****-TS3S-b** relative to***t*****-TS3R-b** that determine the lower energy of ***t*****-TS3S-b**. On the whole, the more advantageous hydrogen bond interaction and π‒π interaction in the favorable carbon-carbon bond formation transition state determine the observed enantioselectivity.

## Discussion

The detailed reaction mechanisms as well as the origin of enantioselectivity for the NHC-catalyzed cross-coupling reaction of aldehyde with enamide were investigated. Our calculated results indicate that the NHC catalyst initiate the nucleophilic attack on aldehyde to afford the Breslow intermediate, which can then react with enamide via hydroacylation reaction or its isomer imine via aza-benzoin reaction, preferentially leading to the experimentally observed *S*-configured product. According to the computational results, the tautomerization step is rate-limiting in aza-benzoin reaction pathway, while for the hydroacylation reactions, the carbon-carbon formation step is rate-determining. NBO analyses reveal that the more polar characteristic of C=N bond in ***t*****-R2-imine** and advantageous stereoelectronic effect in ***t*****-TS3S-b** determine that the reaction between **M2** and ***t*****-R2-imine** is more likely to occur. Further structural analyses on the key enantioselective transition states reveal that the hydrogen bond interaction and π‒π interaction determine the observed enantioselectivity. This present work can help people understand the details of the competing hydroacylation and aza-benzoin reaction pathways for NHC-catalyzed cross-coupling reactions of aldehydes with enamides, and thus provide valuable mechanistic insights for the rational design on the switchable and novel NHC-catalyzed cross-coupling reactions in future.

## Methods

### Computational details

The Gaussian 09^44^ software was used for all theoretical calculations in the present study. The M06-2X method^45^,^46^,^47^ with 6–31 G(d, p) basis set was used for all geometrical optimizations in gas phase. For transition states, the Berny algorithm was employed for both minimizations and optimizations^48^. The corresponding vibrational frequencies were calculated at the same level to identify whether the structure is a transition state or a minimum. It was confirmed that all reactants and intermediates had no imaginary frequencies, and each transition state had only one imaginary frequency. Intrinsic reaction coordinate (IRC) calculations^49^,^50^, at the same level of theory, were also performed to ensure that the transition states led to the expected reactants and products. Afterwards, the single-point energies in solvent methyl *tert*-butyl (MTBE) were refined at the M06-2X/6-311++G(d, p) level^45^,^46^,^47^ using IEFPCM solvent model^51^,^52^. Finally, the Gibbs free energies at the M06-2X/6-311++G(d, p) level in the solvent MTBE are used through the whole paper.

## Additional Information

**How to cite this article**: Qiao, Y. *et al*. Insights into the Competing Mechanisms and Origin of Enantioselectivity for N-Heterocyclic Carbene-Catalyzed Reaction of Aldehyde with Enamide. *Sci. Rep.* **6**, 38200; doi: 10.1038/srep38200 (2016).

**Publisher's note:** Springer Nature remains neutral with regard to jurisdictional claims in published maps and institutional affiliations.

## Supplementary Material

Supplementary Information
