# Supplementary material for: Insights into the Competing Mechanisms and Origin of Enantioselectivity for N-Heterocyclic Carbene-Catalyzed Reaction of Aldehyde with Enamide
Source: Sci Rep. 2016 Dec 1;6:38200. doi: 10.1038/srep38200 (PMC5131292; doi:10.1038/srep38200)
Supplement: Supplementary Information [file srep38200-s1.doc]

**Supporting Information**

**Insights into the Competing Mechanisms and Origin of Enantioselectivity for** **N-Heterocyclic Carbene-Catalyzed Reaction of Aldehyde with Enamide**

Yan Qiao^1,3^, Xinhuan Chen^1,3^, Donghui Wei^2,*^,and Junbiao Chang^1,3,*^

^1^Department of Pathophysiology, School of Basic Medical Sciences, and ^2^The College of Chemistry and Molecular Engineering, Zhengzhou University, Zhengzhou 450001, ^3^Henan Provincial Cooperative Innovation Center for Cancer Chemoprevention, Zhengzhou 450001, Henan, China.

E-mail: [donghuiwei@zzu.edu.cn](mailto:donghuiwei@zzu.edu.cn), [changjunbiao@zzu.edu.cn](mailto:changjunbiao@zzu.edu.cn)

**Supporting Information**

**Table of Contents**

**Table 1**. The ZPE, δG, E+ZPE, and G (stationary points) at the M06-2X/6-31G(d, p) level in gas phase and E at M06-2X/6-311++G (d, p) level in MTBE using IEFPCM model (unit: a.u.)……………..………………………………………………………S2

**Fig. S1** The optimized transition state structures and Breslow intermediates M2 and M2′………..…………………………………………………………………....…….S4

**Fig. S2** Free energy profile for the bimolecular tautomerization between *t*-R2 and *t*-R2-imine. …..………………………………………………………………..….….S5

**Fig. S3** Free energy profile for the bimolecular tautomerization between *c*-R2 and *c*-R2-imine. …..………………………………………………………………..…….S5

**Fig. S4** Free energy profile for the hydroacylation reaction between M2 and *c*-R2...S6

Cartesian Coordinates of all stationary points at M06-2X/6-31G(d, p) level…..……S6

**Table 1．** The ZPE, δG, E, E+ZPE, and G of SP (stationary points) at the M06-2X/6-31G (d,p) level in gas phase, and E at B2, i.e. M06-2X/6-311++G (d,p) level in solvent MTBE (unit: a.u.)

| SP | δE | δG | E+ZPE | G | E/B2 |
| --- | --- | --- | --- | --- | --- |
| **R1**  **HCO_3_^-^**  **t-R2**  **t-2TS1**  **t-2M1**  **t-2TS2**  **t-R2-imine**  **t-TS1-HCO_3_^-^**  **t-M1-HCO_3_^-^**  **t-TS2-HCO_3_^-^**  **c-R2**  **c-2TS1**  **c-2M1**  **c-2TS2**  **c-R2-imine**  **c-TS1-HCO_3_^-^**  **Cat**  **TS1R**  **M1R**  **TS2R**  **M2**  **TS1S**  **M1S**  **TS2S**  **M2′**  **t-TS3S**  **t-M3S**  **t-TS4S**  **P1S**  **t-TS3R**  **t-M3R**  **t-TS4R**  **P1R**  **t-TS3S-b**  **t-M3S-b**  **t-TS4S-b**  **t-TS3R-b**  **t-M3R-b**  **t-TS4R-b**  **c-TS3S**  **c-M3S**  **c-TS4S**  **c-TS3R**  **c-M3R**  **c-TS4R** | 0.101569  0.027188  0.276791  0.551328  0.552973  0.548715  0.275312  0.300409  0.303360  0.298448  0.275790  0.549430  0.550836  0.548225  0.274483  0.298939  0.575876  0.679141  0.680808  0.705406  0.681150  0.679952  0.681419  0.704814  0.680634  0.955747  0.962409  0.960658  0.381162  0.955399  0.959968  0.960478  0.381162  0.957374  0.960940  0.960130  0.958590  0.960875  0.959054  0.955354  0.960572  0.959396  0.954988  0.961417  0.96054 | 0.068866  0.001451  0.234533  0.493224  0.494100  0.490346  0.231824  0.252141  0.254823  0.249278  0.232835  0.488310  0.490886  0.487600  0.231180  0.250568  0.514396  0.607942  0.609898  0.633757  0.611989  0.611855  0.613354  0.631331  0.609703  0.871637  0.880054  0.876613  0.328896  0.872238  0.876351  0.873860  0.328896  0.871165  0.878039  0.876307  0.875027  0.876713  0.871418  0.872529  0.878295  0.876385  0.870270  0.878570  0.876791 | -804.894531  -264.307144  -635.063257  -1270.084443  -1270.096303  -1270.096309  -635.064094  -899.422340  -899.419860  -899.392649  -635.060231  -1270.072821  -1270.072487  -1270.074673  -635.059313  -899.394863  -1100.037363  -1904.944731  -1904.948458  -2169.297754  -1904.966040  -1904.944502  -1904.945636  -2169.292199  -1904.951921  -2540.027063  -2540.049638  -2540.041175  -1440.002054  -2540.023464  -2540.043411  -2540.034981  -1440.002054  -2540.040719  -2540.058899  -2540.044774  -2540.041760  -2540.058336  -2540.051836  -2540.025651  -2540.048381  -2540.038551  -2540.019383  -2540.041040  -2540.031625 | -804.927234  -264.332882  -635.105515  -1270.142547  -1270.155176  -1270.154678  -635.107581  -899.470609  -899.468397  -899.441819  -635.103186  -1270.133942  -1270.132437  -1270.135298  -635.102616  -899.443234  -1100.098843  -1905.015930  -1905.019368  -2169.369402  -1905.035202  -1905.012599  -1905.013701  -2169.365681  -1905.022851  -2540.111173  -2540.131992  -2540.125221  -1440.054320  -2540.106625  -2540.127029  -2540.121599  -1440.054320  -2540.126928  -2540.141800  -2540.128597  -2540.125323  -2540.142498  -2540.139471  -2540.108476  -2540.130658  -2540.121562  -2540.104101  -2540.123887  -2540.115383 | -805.120065540  -264.512756729  -635.50105519  -1270.95229253  -1270.96488488  -1270.95774232  -635.49824761  -900.02546140  -900.02402704  -899.99419865  -635.498594818  -1270.94389485  -1270.94758071  -1270.94351587  -635.493681664  -900.02359105  -1100.86789669  -1905.99785094  -1906.00733717  -2170.51745112  -1906.01957948  -1905.99818441  -1906.00590741  -2170.51438490  -1906.00605998  -2541.50757792  -2541.53673235  -2541.52666648  -1440.66213430  -2541.50617436  -2541.53099728  -2541.52170875  -1440.66213459  -2541.52282641  -2541.54477578  -2541.53008814  -2541.52465909  -2541.54675050  -2541.53650538  -2541.50706376  -2541.53554655  -2541.52379387  -2541.50034682  -2541.52867120  -2541.51773083 |


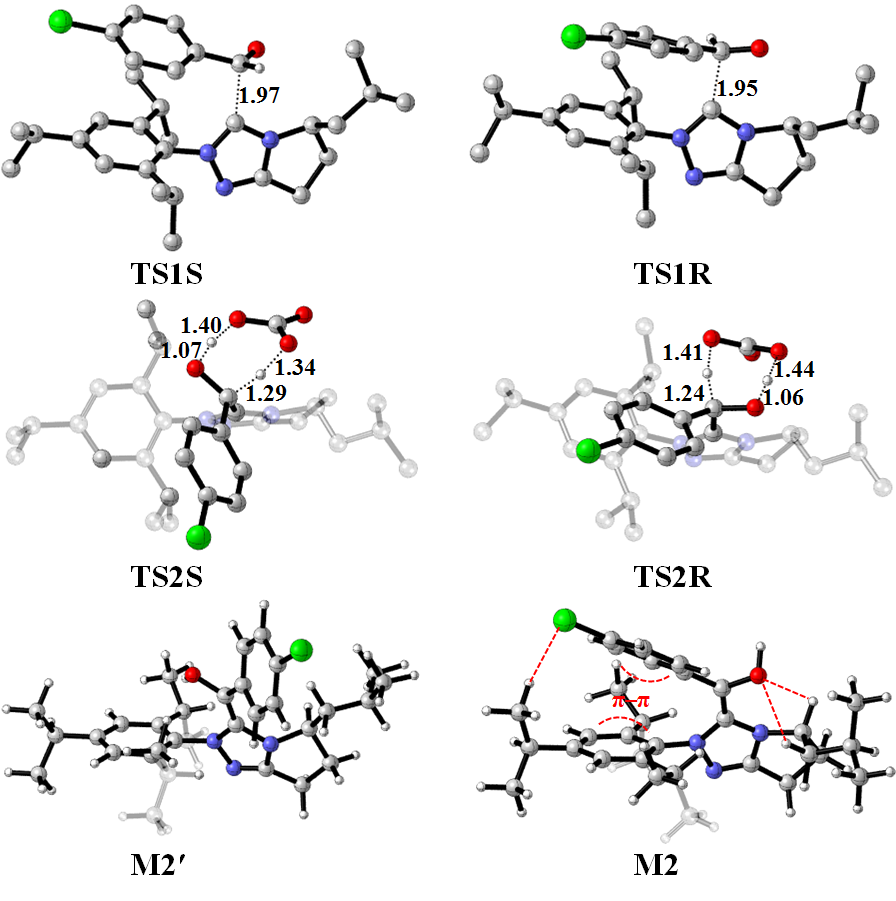


Figure S1. The optimized transition state structures and Breslow intermediates **M2** and **M2′** (the hydrogens not involved in the reaction are omitted, and the NHC catalysts in **TS2S** and **TS2R** are shown in transparent for clarity).

Figure S2. Free energy profile for the bimolecular tautomerization between ***t*-R2** and ***t*-R2-imine**.

Figure S3. Free energy profile for the bimolecular tautomerization between ***c*-R2** and ***c*-R2-imine**.

Figure S4. Free energy profile for the hydroacylation reaction between **M2** and ***c*-R2.**

Cartesian Coordinate for all stationary points

R1

C -0.74987300 1.25739100 0.00000900

C 0.63594600 1.36396700 0.00000400

C 1.43092500 0.21868200 -0.00001600

C 0.83800300 -1.04625600 -0.00002900

C -0.54284100 -1.16796600 -0.00002000

C -1.32203200 -0.01083400 -0.00000200

H -1.38364600 2.13654600 0.00002300

H 1.10319400 2.34574100 0.00001400

H 1.48028500 -1.92108400 -0.00004600

H -1.02331000 -2.13969700 -0.00002900

Cl -3.05644200 -0.15815600 0.00001100

C 2.90893000 0.33960300 -0.00002800

O 3.66166800 -0.60511400 0.00006500

H 3.29529800 1.38053600 -0.00017400

Cat

C 2.96573200 -0.48012400 -2.58095200

C 4.13341700 -0.43490900 -1.55902300

C 3.62741000 0.38057900 -0.33614700

N 2.17914700 0.20072900 -0.46611900

C 1.78735300 -0.27167500 -1.68742000

C 1.11395000 0.41457600 0.35987800

N 0.11871200 0.03945200 -0.48141200

N 0.50012900 -0.39621300 -1.74491500

C -1.27854500 0.01901000 -0.16739700

C -1.86977800 -1.21591100 0.12562000

C -3.23807700 -1.23921800 0.39881200

C -4.00667800 -0.07894500 0.39676900

C -3.37594300 1.13007500 0.10394700

C -2.01499000 1.21217900 -0.19072000

C 4.10435300 -0.07332800 1.04212700

C 5.45499400 0.49404100 1.48863200

C 6.59086500 0.16556900 0.51886200

C 5.78666200 -0.01966600 2.88992400

C -1.42962200 2.59007400 -0.46814600

C -0.82504300 3.18731500 0.81075400

C -0.43760800 2.67609600 -1.63655900

C -1.06478200 -2.50388700 0.11053300

C -1.39587300 -3.41641500 1.29495200

C -1.27380400 -3.23862800 -1.21996000

C -5.49268400 -0.12924500 0.69633100

C -5.84145000 0.69238300 1.94215900

C -6.31994900 0.33215800 -0.50849400

H 3.03406800 0.33628200 -3.30707100

H 2.91944200 -1.41899100 -3.13418800

H 4.36326600 -1.45173100 -1.22430900

H 5.04041300 -0.01544700 -1.99447300

H 3.85432500 1.44785000 -0.46735200

H -3.72034800 -2.18826200 0.62016900

H -3.95911000 2.04923300 0.09436800

H 3.33352800 0.23182600 1.75909800

H 4.13625000 -1.17216800 1.06256200

H 5.35473800 1.58761400 1.53973300

H 7.55199500 0.49883700 0.92133200

H 6.45526900 0.65172500 -0.45102900

H 6.65536200 -0.91621500 0.35082600

H 6.71409200 0.42352600 3.26376300

H 4.98697600 0.21248100 3.59899900

H 5.91789000 -1.10763900 2.87579900

H -2.29131500 3.21062900 -0.74450300

H -1.57187200 3.23615600 1.60846000

H -0.45878000 4.20124400 0.61797300

H 0.00823400 2.56797000 1.15460800

H -0.74986800 2.05682900 -2.48145100

H -0.37081900 3.71512800 -1.97348400

H 0.56677000 2.36390000 -1.33813300

H -0.00612900 -2.23559600 0.18735400

H -1.30827000 -2.88366500 2.24541200

H -0.70955700 -4.26821200 1.31204300

H -2.41055100 -3.82021100 1.22177100

H -0.98317000 -2.60374000 -2.05989600

H -0.67928100 -4.15751900 -1.24867100

H -2.32799200 -3.51230000 -1.33929200

H -5.74672300 -1.17701800 0.90054200

H -5.26217000 0.36188000 2.80809900

H -6.90566700 0.59933500 2.17945900

H -5.62715200 1.75375000 1.77958000

H -6.08533100 -0.25911500 -1.39736800

H -7.39029900 0.23888200 -0.30082900

H -6.11358900 1.38234500 -0.73967500

*t*-R2

C -1.83731600 0.55375900 0.13228600

O -2.21091200 1.65894700 0.48188200

C -2.86421000 -0.55738700 -0.15483100

C -3.78065100 -0.64035200 1.07303800

C -3.67806400 -0.09232600 -1.37168400

C -2.25205000 -1.93241700 -0.43489200

H -3.23000200 -0.99031300 1.95209200

H -4.19456100 0.34429500 1.29701600

H -4.60036600 -1.33951600 0.88072200

H -3.04753300 -0.02167800 -2.26402700

H -4.48386500 -0.80403400 -1.57693500

H -4.11265600 0.89055400 -1.17734900

H -3.05281100 -2.66363900 -0.57636200

H -1.65073900 -1.93910600 -1.35145400

H -1.63487400 -2.28129200 0.40066800

N -0.51630200 0.22789900 -0.04065000

H -0.30879600 -0.69583200 -0.38615700

C 0.60363500 1.06633400 0.13972100

C 0.54923200 2.38317600 0.37326600

H 1.47808300 2.91104900 0.55226100

H -0.38592100 2.91797300 0.42605100

C 1.89459900 0.32901000 0.06444500

C 2.03044200 -0.94712600 0.62409000

C 2.99948800 0.90511200 -0.57071700

C 3.24410800 -1.62446200 0.55869600

H 1.18875400 -1.39869600 1.14310400

C 4.21271200 0.22983900 -0.63207900

H 2.89015200 1.88081500 -1.03355200

C 4.33910300 -1.03832000 -0.06942000

H 3.33648500 -2.60850800 1.00722300

H 5.05893500 0.68952400 -1.13258200

H 5.28496400 -1.56750800 -0.12272500

*c*-R2

C 1.30118500 0.04911300 0.39133300

O 0.68733300 -0.52821800 1.26551000

C 2.74396500 -0.34828700 0.03462800

C 3.48597200 -0.61994000 1.34694900

C 2.62964300 -1.64991900 -0.77569700

C 3.50053700 0.70379200 -0.78201900

H 3.60848800 0.30088600 1.92576200

H 2.92439000 -1.33067900 1.95479800

H 4.47915000 -1.02887700 1.13755800

H 2.10084500 -1.47956100 -1.71966400

H 3.62753600 -2.03574600 -1.00785500

H 2.08442600 -2.40411600 -0.20281200

H 4.53103600 0.37071000 -0.93567600

H 3.06295200 0.84965600 -1.77587500

H 3.54157600 1.66803500 -0.26342000

N 0.72607200 1.02807300 -0.39213800

H 1.33729900 1.61286100 -0.93977500

C -0.60152800 1.49217800 -0.23128100

C -0.85979900 2.80278600 -0.29392600

H -1.88000900 3.16388800 -0.31186700

H -0.06072700 3.53392100 -0.35685300

C -1.67198200 0.46925900 -0.12697300

C -1.56954300 -0.74440100 -0.81320400

C -2.82238800 0.73105500 0.61994900

C -2.60739100 -1.66622000 -0.77018000

H -0.67118500 -0.95686100 -1.38544000

C -3.86144700 -0.19223200 0.66277600

H -2.88651900 1.65292700 1.18962400

C -3.75867100 -1.39221800 -0.03453100

H -2.51791900 -2.60352400 -1.31012800

H -4.74725300 0.02183300 1.25223300

H -4.56656000 -2.11592200 0.00332600

*t*-R2-imine

C 1.84017800 0.21604900 -0.68954000

O 2.19187500 0.91433500 -1.61604700

C 2.81449500 -0.41893300 0.30482400

C 4.23013000 0.04991500 -0.02438600

C 2.42078600 0.02155700 1.72289400

C 2.71617400 -1.94790400 0.19329700

H 4.51044000 -0.24345600 -1.03851000

H 4.30638600 1.13860700 0.03631300

H 4.94071400 -0.39144700 0.68109800

H 1.41489900 -0.32128000 1.98134700

H 3.12272300 -0.40248400 2.44747000

H 2.45443800 1.11211900 1.82226500

H 3.41961600 -2.41069300 0.89297800

H 1.70726100 -2.29455600 0.42814900

H 2.96977600 -2.28268400 -0.81711200

N 0.49994500 -0.14619700 -0.49431600

C -0.40629500 0.69514200 -0.18044300

C -0.14197400 2.15218600 0.11507700

H -0.39331300 2.37265700 1.15739300

H 0.89932700 2.41041500 -0.07119700

C -1.81118700 0.20280400 -0.07392600

C -2.10447500 -1.12398100 -0.41232600

C -2.84709000 1.03768900 0.35460100

C -3.40466100 -1.60073500 -0.32800900

H -1.29270000 -1.76308300 -0.74245000

C -4.15077900 0.55717700 0.44307000

H -2.64596400 2.06950000 0.62275700

C -4.43240200 -0.76092100 0.10100300

H -3.62077700 -2.62981900 -0.59652300

H -4.94564000 1.21544800 0.77815500

H -5.44899300 -1.13519900 0.16776300

H -0.77020000 2.78438200 -0.51812800

*c*-R2-imine

C 1.34197400 0.15069000 0.68079200

O 1.07772500 -0.29251200 1.77719500

C 2.50921500 -0.38022500 -0.16071700

C 3.76015100 0.34780900 0.36333400

C 2.64434000 -1.88372500 0.08537000

C 2.33425500 -0.08380000 -1.65250600

H 3.66263600 1.42928300 0.23171400

H 3.90577300 0.13723800 1.42622000

H 4.64421000 0.00830700 -0.18614200

H 1.77629800 -2.42960000 -0.30041300

H 3.53620900 -2.26588900 -0.42065200

H 2.72206100 -2.08892900 1.15427900

H 3.19414100 -0.47650600 -2.20426300

H 1.43449300 -0.55566200 -2.06192200

H 2.26741000 0.99101000 -1.83652600

N 0.74814000 1.31688800 0.18327800

C -0.49043300 1.48304900 -0.06004100

C -0.95477200 2.87923200 -0.39236900

H -1.53936800 3.28843700 0.43812100

H -0.08640800 3.51788400 -0.54785000

C -1.52304200 0.40086600 -0.05906300

C -1.19879200 -0.91470900 -0.40447100

C -2.85147000 0.70346300 0.25793200

C -2.17212400 -1.90501800 -0.41889300

H -0.17977500 -1.16738700 -0.67903400

C -3.82447700 -0.29030400 0.25900300

H -3.12900400 1.71874000 0.52110200

C -3.48763500 -1.59671200 -0.08090200

H -1.90305300 -2.91929300 -0.69465300

H -4.84742500 -0.04165300 0.52196300

H -4.24735200 -2.37172000 -0.08733300

H -1.59277000 2.88392000 -1.27992700

t-2TS1

C 1.78046800 1.48210900 -0.46207700

O 2.31170700 0.64086800 -1.21282100

C 2.68341800 2.43209700 0.35796400

C 4.13388500 1.95600000 0.30415000

C 2.56462800 3.81914400 -0.29364100

C 2.23091200 2.52273300 1.81907800

H 4.23331600 0.97097600 0.76924500

H 4.47892100 1.87873900 -0.72975500

H 4.77928900 2.65983900 0.84106900

H 1.53061200 4.17072800 -0.24979800

H 3.20894600 4.53813000 0.22511400

H 2.87052300 3.78118900 -1.34428400

H 2.85350500 3.24836000 2.35465500

H 1.18864500 2.84531600 1.88669000

H 2.34020800 1.55533900 2.31977400

N 0.46110700 1.70595000 -0.29561300

C -0.47988100 1.23409300 -1.19996000

C -0.27353000 0.56260800 -2.36046000

H -1.09088800 0.39375400 -3.05063000

H 0.72385500 0.30747300 -2.68287200

C -1.88171900 1.62251200 -0.81499600

C -2.09255800 2.55688800 0.20907800

C -3.01227100 1.06645100 -1.43005300

C -3.37928900 2.90665700 0.61257400

H -1.22262200 3.00472300 0.67572800

C -4.29702000 1.43091900 -1.04481000

H -2.89101800 0.30649500 -2.19457300

C -4.49086700 2.34610600 -0.01167100

H -3.51255800 3.63248400 1.40935700

H -5.15212400 0.97958900 -1.54007300

H -5.49421000 2.62435300 0.29587400

H 0.34836500 -2.04889000 -0.55015400

C 2.00785900 -1.44786500 0.54294900

O 2.43031900 -0.85381100 1.49822400

C 2.85608200 -2.31161600 -0.38711300

C 4.25210600 -1.68943000 -0.47331500

C 2.93254600 -3.69343000 0.29224600

C 2.25824800 -2.43939400 -1.79393900

H 4.18913500 -0.72002000 -0.97251900

H 4.67367100 -1.54780400 0.52424200

H 4.90684200 -2.35788300 -1.04033300

H 1.94193200 -4.14889600 0.40637000

H 3.54140900 -4.36206100 -0.32303700

H 3.39334500 -3.61376500 1.28053900

H 2.99922800 -2.90465500 -2.44936400

H 1.37479300 -3.09199800 -1.82436500

H 2.00900500 -1.45144200 -2.18875800

N 0.59426100 -1.55006500 0.29641800

C -0.42878200 -1.05727100 0.99354800

C -0.25250600 0.02703600 1.93186200

H -1.12343100 0.20211300 2.55549100

H 0.67519800 -0.03984900 2.49652100

C -1.75266500 -1.62490000 0.70098500

C -1.86286200 -2.94254500 0.22099000

C -2.91898600 -0.88994600 0.95644300

C -3.10900900 -3.50490500 -0.00721000

H -0.97415000 -3.55067600 0.07569600

C -4.16375100 -1.45675600 0.71323700

H -2.85912300 0.14177300 1.28978800

C -4.26284000 -2.75977000 0.23531700

H -3.18244200 -4.52724400 -0.36150600

H -5.05513000 -0.86352600 0.88620300

H -5.23846200 -3.19976200 0.05563600

H -0.03805900 0.91430400 1.19811000

t-2M1

C 1.20170700 -1.60553800 0.51307000

O 1.93798400 -0.80063100 1.14360300

C 1.86287800 -2.77238700 -0.25510700

C 3.37928000 -2.73681300 -0.07108000

C 1.29685000 -4.08742200 0.29940700

C 1.52914200 -2.66968900 -1.74888100

H 3.79959700 -1.81404700 -0.48148800

H 3.64576700 -2.78669200 0.98834400

H 3.83853000 -3.58687600 -0.58756700

H 0.21367000 -4.12103900 0.16344500

H 1.75231800 -4.94162000 -0.21457700

H 1.50913400 -4.18108800 1.36953800

H 1.89571400 -3.55470600 -2.28145800

H 0.44681400 -2.59796100 -1.89220700

H 2.01651500 -1.79215300 -2.18759400

N -0.12754800 -1.57359700 0.41197700

C -0.90931000 -0.76391300 1.20455500

C -0.51339300 0.08303200 2.19822800

H -1.23936400 0.63519700 2.78177100

H 0.51370900 0.09666100 2.52899700

C -2.37544600 -0.90462700 0.87659100

C -2.76108900 -1.69539400 -0.21579000

C -3.38822500 -0.28948100 1.62174500

C -4.09827400 -1.83324000 -0.57119800

H -1.97847200 -2.19986600 -0.77033600

C -4.72800300 -0.42952200 1.27160900

H -3.14157300 0.30486000 2.49442200

C -5.09185600 -1.19416300 0.16671000

H -4.36718600 -2.45095800 -1.42339200

H -5.49179800 0.05783800 1.87098900

H -6.13777800 -1.30667800 -0.10272900

H 0.77658200 1.20828100 0.64620600

C 2.56002700 0.97765800 -0.45436900

O 2.98346000 0.45023400 -1.44901500

C 3.44825600 1.65271700 0.58471000

C 4.62895300 0.72118700 0.87206500

C 3.94199900 2.93589800 -0.11574700

C 2.73964700 2.02002100 1.88890000

H 4.26468500 -0.19073900 1.34959100

H 5.14212300 0.45443400 -0.05472500

H 5.33781400 1.22809000 1.53373400

H 3.10971100 3.60514900 -0.36180800

H 4.62009700 3.47347000 0.55318800

H 4.47875500 2.69135700 -1.03569400

H 3.45615600 2.52211100 2.54521800

H 1.91052200 2.72036800 1.73050000

H 2.37435500 1.12397100 2.39236600

N 1.13247200 1.22871800 -0.32077700

C 0.23445500 1.19124100 -1.27522200

C 0.58362800 0.86380900 -2.69144000

H -0.20870000 1.20063300 -3.35586600

H 1.53305400 1.31611200 -2.97567900

C -1.13205000 1.58548900 -0.93874700

C -1.38063600 2.44479800 0.14456800

C -2.21086900 1.11580000 -1.70399800

C -2.67776800 2.83722200 0.43908700

H -0.55461500 2.84672200 0.72278800

C -3.50761600 1.49439400 -1.39241800

H -2.03811800 0.40919400 -2.50890600

C -3.73980500 2.36000600 -0.32603300

H -2.86092800 3.51444800 1.26613200

H -4.33835700 1.09160800 -1.96041200

H -4.75599400 2.65362500 -0.08289000

H 0.71634700 -0.21887600 -2.78229900

t-2TS2

C 1.41038600 -1.69313300 0.52207800

O 2.09989100 -0.85704000 1.13312700

C 2.10566700 -2.79929500 -0.29141800

C 3.62111300 -2.68071500 -0.14016500

C 1.62038100 -4.16405200 0.21443300

C 1.72308600 -2.63646300 -1.76995000

H 3.97305700 -1.71742400 -0.52082600

H 3.91571600 -2.75438200 0.91039000

H 4.11655300 -3.48121700 -0.69985600

H 0.53544500 -4.24192600 0.11552000

H 2.09236000 -4.96763600 -0.36184100

H 1.87992500 -4.30496000 1.26880400

H 2.18137500 -3.43408800 -2.36544100

H 0.63746300 -2.68685800 -1.89096300

H 2.08611300 -1.67644900 -2.15240600

N 0.05557800 -1.73770200 0.44945900

C -0.71574300 -0.87646800 1.13363500

C -0.30521500 0.16931600 1.96769900

H -1.06320000 0.76207500 2.46809800

H 0.65031600 0.08133400 2.46466500

C -2.18353700 -1.06301200 0.84283800

C -2.57231500 -1.84580700 -0.25396500

C -3.18564300 -0.51305100 1.64756100

C -3.91414600 -2.04785600 -0.55024900

H -1.78947500 -2.29424300 -0.85534500

C -4.53229900 -0.71989400 1.35632800

H -2.92077900 0.06352700 2.52698900

C -4.90306000 -1.48227300 0.25363300

H -4.19180300 -2.65700900 -1.40546600

H -5.29236000 -0.29051700 2.00244300

H -5.95286700 -1.64854700 0.03107300

C 2.35362800 1.12339100 -0.41534800

O 2.79171300 0.58015800 -1.39635300

C 3.22354600 1.87501100 0.58232800

C 4.56439500 1.14631900 0.68949100

C 3.41579800 3.26353500 -0.06515000

C 2.60349800 2.03972300 1.96992700

H 4.40360800 0.15078000 1.11038300

H 5.03073800 1.04075200 -0.29234400

H 5.23607200 1.71292100 1.34146000

H 2.46066100 3.79081000 -0.16858000

H 4.06884200 3.86980000 0.56908200

H 3.87712400 3.17288100 -1.05253400

H 3.26395900 2.67032700 2.57284600

H 1.62375800 2.52778100 1.93303700

H 2.50066000 1.07023100 2.45761800

N 0.91793100 1.29738300 -0.24589700

C 0.08451200 1.32264800 -1.24651800

C 0.46365800 1.09590900 -2.67913000

H -0.33817800 1.43309300 -3.33350700

H 1.39252700 1.61119200 -2.92553800

C -1.31506800 1.63798700 -0.92248400

C -1.60654600 2.47360600 0.16511600

C -2.36367900 1.09894200 -1.67983700

C -2.92501500 2.76347500 0.48761200

H -0.79217800 2.91404700 0.73164900

C -3.68058800 1.36996600 -1.33790500

H -2.14833200 0.42170000 -2.50038500

C -3.96028400 2.20634800 -0.25924200

H -3.14552900 3.41989700 1.32256900

H -4.48885000 0.91423600 -1.89865000

H -4.99187700 2.41793500 0.00406000

H 0.65521300 0.02936400 -2.83326400

H 0.42736000 0.99291600 0.77922800

t-TS1-HCO_3_^-^

C -1.94661500 -1.02970100 0.10976300

O -2.33711000 -2.12805700 0.50468300

C -2.98253100 -0.00655400 -0.41817200

C -4.34620100 -0.69430900 -0.49517000

C -3.06518100 1.17006700 0.56563900

C -2.58967100 0.50130600 -1.81289200

H -4.31704500 -1.54113400 -1.18643700

H -4.64011100 -1.08297600 0.48220100

H -5.10229300 0.01990900 -0.84150900

H -2.11143800 1.69459300 0.63967700

H -3.82247600 1.88748100 0.22767800

H -3.35123700 0.81785600 1.56285700

H -3.38900400 1.13905300 -2.21017100

H -1.67023100 1.08878200 -1.78610800

H -2.45435500 -0.33863100 -2.50340200

N -0.65346900 -0.59827200 0.09009400

C 0.40146000 -1.43651700 0.48451600

C 0.33216200 -2.57203300 1.20598700

H -0.10446700 0.85739400 1.62253500

H -0.60566500 -2.96260800 1.56613500

C 1.73996500 -0.97528100 -0.00128200

C 1.88512000 -0.44747700 -1.28921200

C 2.87829600 -1.08764300 0.80316500

C 3.13525000 -0.07255300 -1.76684600

H 1.00072500 -0.32597700 -1.90637600

C 4.13025900 -0.71148900 0.32724600

H 2.76610000 -1.45479500 1.81878500

C 4.26446600 -0.20639700 -0.96289800

H 3.22664300 0.33733200 -2.76827400

H 4.99999300 -0.79489000 0.97289600

H 5.23890500 0.09953700 -1.33245900

H 1.24534800 -3.12855400 1.38828400

C 0.48252000 2.37656200 0.60228300

O 0.04877700 1.73447300 -0.44712100

O 0.30164800 1.72044600 1.79598100

O 0.99404800 3.47321200 0.61466500

H -0.30979100 0.62705200 -0.23540400

t-M1-HCO_3_^-^

C 1.94430200 -1.01905000 -0.12667600

O 2.34621400 -2.11146900 -0.53398700

C 2.98444000 -0.00269400 0.41533400

C 4.35353700 -0.68275800 0.45896500

C 3.05260900 1.20335200 -0.53189300

C 2.60685800 0.46327100 1.82828500

H 4.33502300 -1.55213400 1.12178700

H 4.63951900 -1.03925100 -0.53294300

H 5.10995100 0.02404200 0.82030200

H 2.10111200 1.73558600 -0.56944700

H 3.82125400 1.90750200 -0.19081800

H 3.31153700 0.88242500 -1.54684200

H 3.40489700 1.09622200 2.23641600

H 1.68259200 1.04359100 1.83205400

H 2.48263200 -0.39635100 2.49614100

N 0.65527000 -0.58454100 -0.09272000

C -0.38615800 -1.44067200 -0.48174800

C -0.32127400 -2.58226800 -1.19709500

H 0.13235900 0.80862100 -1.50901700

H 0.61463400 -2.97549100 -1.55915800

C -1.73014100 -0.98736800 0.00062100

C -1.88377100 -0.47898900 1.29544300

C -2.86412400 -1.08242000 -0.81272100

C -3.13506300 -0.10311800 1.77000800

H -1.00261400 -0.37868900 1.92173000

C -4.11740700 -0.70499800 -0.34080900

H -2.74606600 -1.43820600 -1.83178900

C -4.25890500 -0.21700500 0.95526300

H -3.23205600 0.29029900 2.77764000

H -4.98301400 -0.77455000 -0.99376700

H -5.23438500 0.08950600 1.32167800

H -1.23605700 -3.13900000 -1.37327300

C -0.50658400 2.38222100 -0.62105000

O -0.10481300 1.80598400 0.49822600

O -0.28013000 1.65933300 -1.74836300

O -1.01783100 3.47257400 -0.68728900

H 0.23435300 0.81125900 0.32809000

t-TS2-HCO_3_^-^

C -1.90712300 -1.31727300 0.38330600

O -2.27635200 -2.02983400 1.30361000

C -2.92662200 -0.51250300 -0.44158100

C -4.29218500 -1.19489700 -0.35949800

C -2.99592100 0.87236800 0.22747100

C -2.49691900 -0.35586200 -1.90310100

H -4.26969200 -2.16914600 -0.85932300

H -4.57401700 -1.36146000 0.68178300

H -5.05318700 -0.57496000 -0.84668600

H -2.01822900 1.36394400 0.24378900

H -3.68784200 1.51900100 -0.32389900

H -3.34953000 0.78229600 1.25972900

H -3.28869000 0.15737400 -2.46157200

H -1.58215900 0.23318800 -1.99169400

H -2.32217700 -1.33094500 -2.36848900

N -0.58759000 -1.20004300 -0.01459300

C 0.32711200 -0.71898300 0.78571700

C 0.10972000 0.09894600 1.94179600

H 0.17456600 1.41519500 1.42571200

C 1.73888700 -0.87328800 0.30215000

C 2.10485700 -2.02504300 -0.39856600

C 2.69307900 0.12413600 0.52448200

C 3.41120500 -2.19446900 -0.84243100

H 1.34177700 -2.77394800 -0.58495400

C 3.99699900 -0.04059600 0.06561500

H 2.39708400 1.04219000 1.02535700

C 4.36194200 -1.20216700 -0.60976500

H 3.68879200 -3.09832200 -1.37739100

H 4.72689800 0.74702800 0.22656900

H 5.38075800 -1.32939900 -0.96469300

C 0.34257400 2.89554200 -0.17291600

O 0.18556000 1.86346400 -1.07147700

O 0.26667300 2.57955200 1.08496400

O 0.53182800 4.00585500 -0.62234100

H 0.03423500 1.02215700 -0.61906100

H 0.92164400 0.08108100 2.66803700

H -0.87439800 -0.01273800 2.39481400

c-2TS1

C -1.37225100 -2.30863200 0.17125200

O -2.45985800 -2.81093100 0.23321100

C -0.19311300 -2.81960900 -0.64058200

C -0.63821600 -4.07700100 -1.38583000

C 0.20349500 -1.70880300 -1.62866800

C 0.99094800 -3.14836900 0.28627300

H -0.92375700 -4.86691500 -0.68688600

H -1.49774400 -3.87058100 -2.02813000

H 0.18610400 -4.43861300 -2.00626600

H 0.40492400 -0.76396100 -1.11172800

H 1.11609400 -2.00197100 -2.15589100

H -0.58317400 -1.54800600 -2.37440100

H 1.79981200 -3.56513700 -0.32081100

H 1.39659400 -2.26746400 0.79047200

H 0.70809300 -3.89218600 1.03743500

N -1.10325000 -1.08771400 0.90591700

C -1.96713800 -0.13291200 1.21765700

C -1.50582800 0.87916300 2.14075900

H -1.14476500 0.45869500 3.07977000

H -0.49449200 1.23693000 1.59638200

C -3.25555000 0.00325400 0.52035900

C -3.35934700 -0.20890100 -0.86033900

C -4.37280600 0.43448700 1.24251700

C -4.56868300 0.00058200 -1.50437700

H -2.48238700 -0.50437400 -1.42867500

C -5.59125200 0.61293700 0.59794100

H -4.28931800 0.60245900 2.31153600

C -5.68791500 0.40068700 -0.77404100

H -4.64117600 -0.14559300 -2.57646500

H -6.46118100 0.92518300 1.16515900

H -6.63563600 0.55511700 -1.27941000

C 1.45020600 2.30606500 0.18522600

O 2.60156500 2.72583200 0.28031500

C 0.47994400 3.06608000 -0.74984700

C 0.49210400 4.54283700 -0.34138800

C 1.06291600 2.92986400 -2.16530900

C -0.94823700 2.52598500 -0.74640900

H 0.07425100 4.67303600 0.66273000

H 1.51923900 4.91272300 -0.33260100

H -0.10300200 5.14192400 -1.03955100

H 0.99265700 1.89381600 -2.51982500

H 0.50841100 3.56078400 -2.86895100

H 2.11406200 3.22646600 -2.16610700

H -1.53465400 3.00323900 -1.53980700

H -0.95164700 1.44385800 -0.91943500

H -1.45701700 2.72399800 0.20195600

N 0.96371100 1.19417200 0.80430300

C 1.88207100 0.33225900 1.39565500

C 1.63580500 -0.25874900 2.59255700

H 0.86474200 0.11219900 3.25469100

C 3.07905800 -0.12377700 0.60782200

C 3.08594000 -0.07949300 -0.79035000

C 4.20012200 -0.65603800 1.25334200

C 4.15072000 -0.59408400 -1.51967300

H 2.24916200 0.37736700 -1.30635600

C 5.27002600 -1.16942500 0.52695500

H 4.24842700 -0.63906800 2.33694500

C 5.24573300 -1.15210700 -0.86400300

H 4.13113900 -0.54683800 -2.60440900

H 6.13093000 -1.57248800 1.05150900

H 6.08113300 -1.54971800 -1.43181900

H -2.21410600 1.68851800 2.29301800

H 2.26371500 -1.05660500 2.96852800

H -0.14482300 -0.94900300 1.26266000

c-2M1

C -1.41437900 -2.11401800 0.05938100

O -2.41121500 -2.73924000 0.27055500

C -0.30561200 -2.46121700 -0.91471000

C -0.84542600 -3.52149000 -1.87655800

C 0.14772800 -1.20793200 -1.67356600

C 0.86694200 -3.04003100 -0.09394600

H -1.16446800 -4.41629300 -1.33820100

H -1.70187600 -3.13983100 -2.44038200

H -0.05878800 -3.79574100 -2.58422100

H 0.43863100 -0.39446300 -0.99811800

H 1.01882300 -1.46181400 -2.28444000

H -0.63596400 -0.84582000 -2.34893700

H 1.68147600 -3.28837200 -0.78031400

H 1.26364000 -2.32254000 0.62931200

H 0.55934500 -3.94903600 0.43077400

N -1.16907300 -0.89307200 0.86021500

C -2.06122300 -0.00674000 1.20383600

C -1.67080700 1.01843400 2.20400900

H -1.53551800 0.56600000 3.19264500

H -0.68065500 1.40151000 1.87332900

C -3.38843600 0.04383200 0.56870200

C -3.52944100 -0.13381500 -0.81244400

C -4.50780800 0.34966400 1.35085100

C -4.78136200 -0.02447000 -1.39989600

H -2.65290300 -0.32029600 -1.42622200

C -5.76400300 0.42482400 0.76237000

H -4.39626900 0.50093700 2.42003100

C -5.89951700 0.24250200 -0.61146000

H -4.88527700 -0.14278600 -2.47277200

H -6.63399700 0.63927900 1.37316000

H -6.87870400 0.32060400 -1.07229300

C 1.66295300 2.21968900 0.15057300

O 2.84060900 2.56735000 0.24469400

C 0.75002800 3.01783500 -0.81790700

C 1.20326500 2.63710100 -2.23549800

C -0.73963300 2.71023300 -0.66555000

C 1.00138200 4.51135500 -0.59525800

H 2.28227400 2.78543600 -2.33258100

H 0.97504200 1.58490000 -2.45019600

H 0.68940400 3.25003500 -2.98491100

H -1.10829000 3.01861400 0.31812200

H -1.31716300 3.25125700 -1.42468300

H -0.93166800 1.63743200 -0.78443900

H 0.45763200 5.11129600 -1.33368000

H 0.67275300 4.81659100 0.40391300

H 2.07010200 4.71792400 -0.67397300

N 1.07203500 1.18000500 0.78477000

C 1.86711400 0.23448600 1.39855900

C 1.44019600 -0.40421900 2.52489400

H 0.59884300 -0.02493800 3.09158400

C 3.10926000 -0.29202400 0.72449600

C 3.27200400 -0.22932400 -0.66455000

C 4.10851900 -0.92527700 1.47243400

C 4.36634700 -0.82078400 -1.28466800

H 2.54920900 0.30884900 -1.26845400

C 5.20726800 -1.51567100 0.85496200

H 4.03660600 -0.92887800 2.55480600

C 5.33532900 -1.47736100 -0.52943600

H 4.47033900 -0.75484000 -2.36366900

H 5.96932400 -1.99697000 1.46018700

H 6.19272400 -1.93385300 -1.01423900

H -2.41160000 1.81355400 2.25535100

H 1.92953400 -1.28950000 2.91000800

H -0.20257200 -0.76515300 1.22890000

c-2TS2

C -1.42942400 -2.09141200 0.11162900

O -2.40910300 -2.73994900 0.35039900

C -0.37268300 -2.42583900 -0.92781200

C -0.98049100 -3.42581200 -1.91243000

C 0.09505800 -1.15856000 -1.65459800

C 0.81038900 -3.07710100 -0.18196300

H -1.30307300 -4.33450600 -1.40044800

H -1.85024100 -2.99702500 -2.41958500

H -0.23362000 -3.68913200 -2.66627700

H 0.40579700 -0.37247800 -0.95710900

H 0.95307600 -1.40825000 -2.28567300

H -0.68821500 -0.75929500 -2.30878600

H 1.59017500 -3.32197200 -0.90923900

H 1.25358500 -2.40631600 0.55729500

H 0.49147300 -3.99759300 0.31553900

N -1.15088200 -0.90834500 0.93276700

C -2.03342100 -0.00487300 1.22684400

C -1.68916600 1.02569900 2.25034400

H -1.60448900 0.56261800 3.23821800

H -0.70131300 1.43256000 1.97050200

C -3.34261800 0.07623200 0.54455000

C -3.45065800 -0.09972400 -0.83848400

C -4.48094600 0.38877900 1.29460500

C -4.68480100 0.02254900 -1.46121800

H -2.56033400 -0.30158400 -1.42627800

C -5.71997000 0.47898100 0.67211200

H -4.39944700 0.53513100 2.36727000

C -5.82178900 0.30091600 -0.70498000

H -4.76004600 -0.09703200 -2.53649400

H -6.60387300 0.69941400 1.26055800

H -6.78742400 0.38917900 -1.19187700

C 1.76078000 2.25125800 0.19417500

O 2.94510600 2.55439900 0.29882200

C 0.87024700 3.07732700 -0.76511700

C 1.29888500 2.68590400 -2.18828400

C -0.62542100 2.81203800 -0.59620300

C 1.17091300 4.56216100 -0.54047500

H 2.38099300 2.79933200 -2.29829800

H 1.03158000 1.64410200 -2.40727400

H 0.79892100 3.32045900 -2.92858300

H -0.97363800 3.12447600 0.39334000

H -1.19354500 3.37319500 -1.34729600

H -0.85173600 1.74624500 -0.71634400

H 0.63512600 5.17848300 -1.27084800

H 0.86249700 4.87389000 0.46286300

H 2.24357700 4.74024100 -0.63384400

N 1.12473200 1.23848300 0.85144500

C 1.79714900 0.19076200 1.36374000

C 1.24050400 -0.54282600 2.40727900

H 0.60263500 -0.03190400 3.12063700

C 3.02163700 -0.37774500 0.69643000

C 3.22384500 -0.26798500 -0.68411800

C 3.96252800 -1.08737200 1.44812700

C 4.31084100 -0.87761000 -1.29673200

H 2.52434100 0.30678400 -1.28061600

C 5.05892600 -1.69203800 0.83891600

H 3.84688200 -1.14279500 2.52541400

C 5.23127000 -1.59855200 -0.53761000

H 4.44719900 -0.78117900 -2.36959900

H 5.78103300 -2.23163600 1.44367700

H 6.08318100 -2.07145900 -1.01607400

H -2.43765600 1.81528900 2.27462300

H 1.72383900 -1.44669500 2.75834400

H -0.14174400 -0.84805600 1.44666300

c-TS1-HCO_3_^-^

C -0.13020200 1.69238700 -0.72010700

O -1.07334800 2.30590000 -1.19776800

C 0.65789000 2.25106100 0.47675300

C -0.05203600 3.50012500 0.99665700

C 0.71176700 1.17506200 1.57073800

C 2.08377900 2.60818800 0.03015600

H -0.10874400 4.26622100 0.21913900

H -1.07561500 3.26634800 1.30220800

H 0.49021900 3.90595200 1.85801000

H 1.20389300 0.26539200 1.21554900

H 1.26839100 1.55284900 2.43576800

H -0.29744000 0.90553100 1.90265200

H 2.61600500 3.08232900 0.86314600

H 2.64408500 1.71979100 -0.26737000

H 2.06319900 3.31544800 -0.80615100

N 0.36406200 0.51345600 -1.24222800

H 2.17359400 -0.32814800 -0.85090800

C -0.28674100 -0.61875200 -1.24356500

C 0.31252400 -1.81663100 -1.74770700

H 1.05518200 -1.65521700 -2.52926100

H 1.07567300 -2.10049000 -0.63078400

C -1.56974100 -0.79165700 -0.48153500

C -1.57520800 -1.66816700 0.61057300

C -2.74096400 -0.11166400 -0.82584200

C -2.74138600 -1.85898000 1.34571000

H -0.65077100 -2.17143700 0.88537400

C -3.90695500 -0.31840500 -0.09327600

H -2.72012300 0.58810900 -1.65299000

C -3.91140500 -1.19046300 0.99208700

H -2.73456000 -2.53042300 2.19937700

H -4.81414600 0.21168500 -0.36868900

H -4.82205600 -1.34406200 1.56438400

H -0.37420800 -2.63702300 -1.94185300

C 2.95478600 -1.69857900 0.29270000

O 3.95748500 -2.07252800 0.86630400

O 1.76365200 -2.21566500 0.39481000

O 3.05500500 -0.62123100 -0.55360400

TS1R

C 3.77179400 -2.46429400 1.48974900

C 4.73954500 -1.91809700 0.40402000

C 4.04760900 -0.66952800 -0.19804200

N 2.63360700 -1.03301900 0.00436700

C 2.45497600 -1.98361500 0.96860200

C 1.43657700 -0.60809200 -0.44171800

N 0.58222800 -1.34099800 0.29533400

N 1.19384400 -2.20861100 1.18225200

C -0.85218100 -1.23086100 0.27383100

C -1.46978700 -0.40609800 1.22474400

C -2.85764500 -0.29450300 1.16895300

C -3.61422500 -0.96734700 0.20878600

C -2.95439700 -1.76429700 -0.72509900

C -1.56675800 -1.91310100 -0.71764600

C 4.35260600 0.62929400 0.54734200

C 5.74795300 1.20106500 0.27102000

C 5.90875800 2.51604600 1.03477500

C 5.96989600 1.42035700 -1.22872700

C -0.85120100 -2.79821400 -1.72456800

C -0.53826500 -4.16228500 -1.09181600

C -1.62340300 -2.98408300 -3.03107300

C -0.66685100 0.30680600 2.29974700

C -1.29853200 1.62684500 2.74759600

C -0.47794200 -0.61657200 3.51401000

C -5.12693000 -0.85233700 0.19902400

C -5.72543200 -1.49003100 1.45872600

C -5.59242500 0.59983500 0.05565500

H 3.81095900 -3.54908300 1.59395100

H 3.96720300 -2.02267600 2.47180900

H 5.72258800 -1.67319800 0.81047300

H 4.87356900 -2.67618200 -0.37225500

H 4.22152200 -0.54171000 -1.26533100

H -3.36339800 0.34250200 1.88897100

H -3.54239800 -2.27741200 -1.48050800

H 3.60617500 1.36084100 0.22035700

H 4.22255700 0.45981200 1.62668200

H 6.50849400 0.49887100 0.64370000

H 6.91414000 2.92638000 0.90474000

H 5.73101300 2.38302500 2.10661000

H 5.19245700 3.25659900 0.66259400

H 6.86353800 2.02739400 -1.40175700

H 6.10949300 0.47622900 -1.76373500

H 5.10346700 1.92370600 -1.67062700

H 0.10028900 -2.31109300 -1.97220800

H 0.04492400 -4.05264500 -0.17420900

H 0.02106300 -4.79079200 -1.79168000

H -1.47157400 -4.67722300 -0.84098300

H -1.92673700 -2.02727600 -3.46683700

H -0.99703900 -3.50734000 -3.75833400

H -2.52274300 -3.58988800 -2.88098900

H 0.32005100 0.53610400 1.87696400

H -1.56306800 2.25903700 1.89688500

H -0.59512300 2.17408800 3.38150400

H -2.20079500 1.45321500 3.34319200

H 0.00651900 -1.55415600 3.23555000

H 0.12679100 -0.11862200 4.27864900

H -1.45388300 -0.84999400 3.95338700

H -5.49085800 -1.41453400 -0.67029000

H -5.40916700 -2.53113300 1.56388300

H -6.81848500 -1.45994300 1.42293800

H -5.40328100 -0.94801200 2.35392000

H -5.21057300 1.05633300 -0.86108800

H -6.68517900 0.65011600 0.03313100

H -5.24565600 1.21069000 0.89507400

C 1.48417500 0.70977200 -1.87719200

O 2.61384100 1.24606100 -1.83641200

C 0.24081500 1.50007900 -1.52080300

C 0.35633900 2.64941500 -0.74452400

C -1.01456600 1.08610500 -1.95641100

C -0.77869200 3.35924200 -0.36676200

H 1.35087200 2.97414100 -0.45305800

C -2.16188600 1.77651700 -1.58235400

H -1.09845200 0.19777400 -2.57991400

C -2.02796200 2.90258800 -0.77863700

H -0.70904600 4.25207800 0.24570700

H -3.14312500 1.44777000 -1.90563600

Cl -3.46297200 3.75868500 -0.25920300

H 1.25315800 -0.04519700 -2.66442400

M1R

C 3.63088100 -2.55900400 1.62781600

C 4.60589300 -2.06333400 0.52486900

C 3.93387000 -0.83548300 -0.14342800

N 2.51293800 -1.13431000 0.12677100

C 2.32115700 -2.07321600 1.09530800

C 1.31600700 -0.72119000 -0.31086200

N 0.44646900 -1.44496200 0.41184400

N 1.05591900 -2.29833500 1.30071900

C -0.98407200 -1.38296000 0.31285400

C -1.66923900 -0.52388100 1.18088500

C -3.04776000 -0.42385800 1.01451900

C -3.72296500 -1.14011100 0.02430600

C -2.99647400 -1.99317800 -0.80534000

C -1.61327900 -2.13530200 -0.68407900

C 4.30653800 0.50793100 0.47511400

C 5.67062500 1.03876900 0.02106200

C 5.92114700 2.39592500 0.67965700

C 5.72749700 1.16261500 -1.50611400

C -0.82932000 -3.09683000 -1.56010500

C -0.71646500 -4.45661400 -0.85560400

C -1.41939500 -3.25133900 -2.96213200

C -0.93629000 0.25266100 2.26030900

C -1.57065600 1.61133900 2.56045000

C -0.84455600 -0.59617900 3.53711800

C -5.21412400 -0.95513800 -0.17860000

C -6.00850800 -1.20234700 1.10718900

C -5.50205600 0.44271100 -0.74007100

H 3.64898200 -3.64027200 1.76683800

H 3.83249300 -2.08922600 2.59542600

H 5.58646400 -1.80462900 0.92926400

H 4.74486300 -2.85562500 -0.21522000

H 4.04687300 -0.78934900 -1.22384400

H -3.60694800 0.25260800 1.65451000

H -3.52484400 -2.54916400 -1.57436200

H 3.53901700 1.21386600 0.14322200

H 4.27146700 0.43043700 1.57222400

H 6.46041500 0.34886300 0.35496600

H 6.91213600 2.78026600 0.42257200

H 5.85115400 2.33390900 1.77033700

H 5.17869300 3.12378400 0.33385200

H 6.58069300 1.77543700 -1.81190300

H 5.83602000 0.18878600 -1.99254900

H 4.80274100 1.61167200 -1.88497900

H 0.18339400 -2.69471700 -1.67909600

H -0.24628900 -4.35283100 0.12607200

H -0.12321300 -5.15109200 -1.45811200

H -1.71180100 -4.89028000 -0.71358800

H -1.56745000 -2.27933800 -3.44137800

H -0.74233200 -3.84142600 -3.58511800

H -2.37971700 -3.77604600 -2.93958800

H 0.08079000 0.44498100 1.89689100

H -1.72080700 2.19125700 1.64668500

H -0.91760700 2.18188200 3.22632000

H -2.53410800 1.50113600 3.06885900

H -0.34799100 -1.55088000 3.34836400

H -0.29066400 -0.05770400 4.31212100

H -1.85027800 -0.80140400 3.91946000

H -5.53680800 -1.69354500 -0.92316600

H -5.79986200 -2.19332300 1.51850700

H -7.08193800 -1.12822300 0.91080400

H -5.76122400 -0.45952000 1.87215800

H -4.97160900 0.60432000 -1.68322100

H -6.57292900 0.57692400 -0.91903800

H -5.17754700 1.21382700 -0.03280300

C 1.18673200 0.28439200 -1.52918000

O 2.39119100 0.67176700 -1.86497500

C 0.20806900 1.42147200 -1.14566600

C 0.75743800 2.61407200 -0.67771000

C -1.16882100 1.34992300 -1.35176000

C -0.04977300 3.70539500 -0.37333600

H 1.83947600 2.67550300 -0.60899400

C -1.99419900 2.43509800 -1.06744700

H -1.61272300 0.43861300 -1.74893500

C -1.42280000 3.60144200 -0.57259200

H 0.37191700 4.63447600 -0.00531900

H -3.06573300 2.38056400 -1.22816900

Cl -2.45466700 4.96085500 -0.18533000

H 0.61874400 -0.35262900 -2.26957400

TS2R

C 3.55662500 -2.03419100 2.05174500

C 4.45568600 -1.51890100 0.90231300

C 3.80159800 -0.21849400 0.37778700

N 2.38691200 -0.47428400 0.73095000

C 2.23359400 -1.51170100 1.60255100

C 1.16418600 -0.11333000 0.28885100

N 0.32442100 -0.93895100 0.95991100

N 0.98981600 -1.82341300 1.78246000

C -1.06334500 -1.17203100 0.68742300

C -2.02175500 -0.37209700 1.32477700

C -3.35627200 -0.58870900 0.99780800

C -3.72975100 -1.57026300 0.07544600

C -2.74366200 -2.36348800 -0.50358100

C -1.38535100 -2.19433300 -0.21406500

C 4.30033500 1.07856800 1.00416500

C 5.59772400 1.56806300 0.34994900

C 6.18067100 2.73287900 1.15017300

C 5.35730400 1.98018000 -1.10574900

C -0.33563800 -3.11850900 -0.80513900

C -0.22593300 -4.37785600 0.06780100

C -0.61485600 -3.49799400 -2.25921400

C -1.59640200 0.67828900 2.33601500

C -2.62715900 1.78838000 2.53576000

C -1.26456200 0.00284200 3.67479500

C -5.18541100 -1.74542500 -0.31435300

C -6.08129300 -2.00639400 0.90015300

C -5.68066600 -0.52977500 -1.10627600

H 3.55026500 -3.12067100 2.14400200

H 3.82418700 -1.60231800 3.02287700

H 5.48889900 -1.35394700 1.21847400

H 4.41785700 -2.23064000 0.07246000

H 3.85686800 -0.19927200 -0.71675300

H -4.12054300 0.03986000 1.44604200

H -3.03310100 -3.13405300 -1.21374500

H 3.52805000 1.84132800 0.85189500

H 4.44544600 0.94686500 2.08825800

H 6.32874200 0.74489700 0.36474100

H 7.11059100 3.09729900 0.70168600

H 6.39036800 2.44763600 2.18684700

H 5.47052700 3.56797900 1.16923700

H 4.60395000 2.77527700 -1.14626200

H 6.28366700 2.35985600 -1.55125600

H 4.98627200 1.15638900 -1.72333000

H 0.63794600 -2.61788700 -0.82559000

H 0.01555500 -4.12572300 1.10358800

H 0.56504900 -5.02235600 -0.32778200

H -1.16784900 -4.94128000 0.05817200

H -0.78179400 -2.60553800 -2.86284800

H 0.28388100 -3.97553700 -2.65751400

H -1.45900400 -4.19508600 -2.35161700

H -0.68293100 1.14818400 1.95329900

H -2.92909900 2.23041800 1.58232100

H -2.19953200 2.57948400 3.15862900

H -3.52088400 1.41501200 3.04874800

H -0.49780800 -0.76649900 3.55374300

H -0.90995700 0.74322200 4.39955700

H -2.16289600 -0.47284600 4.08384300

H -5.24226900 -2.62269300 -0.97096000

H -5.73124500 -2.87173200 1.46926500

H -7.11332000 -2.19185100 0.58505600

H -6.08976700 -1.14242200 1.57239700

H -5.06690700 -0.37120600 -1.99775200

H -6.72106600 -0.66349100 -1.42076600

H -5.62510800 0.37695500 -0.49372300

C 0.91144500 0.79481900 -0.85671100

O 2.06463600 1.53909100 -1.13948500

C -0.30625800 1.68259400 -0.78176200

C -0.16318700 3.00283700 -0.34037900

C -1.55626900 1.26847200 -1.24807600

C -1.24213400 3.87934100 -0.32308000

H 0.82505800 3.33499100 -0.03843600

C -2.64915900 2.13212100 -1.23967800

H -1.67416100 0.25668000 -1.62878100

C -2.47855800 3.42885800 -0.77418100

H -1.13239700 4.90052900 0.02588800

H -3.61922300 1.80322000 -1.59675400

Cl -3.85409700 4.52234900 -0.74551000

H 2.56153200 0.91418900 -1.84110500

C 2.38610800 -1.28429900 -2.27925100

O 1.12020000 -1.14237700 -2.59939400

O 2.81167500 -2.33737100 -1.73560600

O 3.16472000 -0.23780100 -2.45431000

H 0.80197600 -0.05166000 -1.76136300

M2

C -4.07269800 2.68131700 0.01753100

C -4.95628100 1.62734400 -0.69933300

C -4.14087700 0.30874400 -0.69060500

N -2.78322100 0.84688600 -0.69906900

C -2.70193500 2.10504900 -0.16236600

C -1.50619500 0.29753400 -0.70331300

N -0.73260300 1.33232600 -0.17303200

N -1.50492300 2.45901900 0.14515800

C 0.62478800 1.27953900 0.25345400

C 0.99666300 0.40379600 1.29253400

C 2.34947300 0.30879600 1.60478800

C 3.31572700 1.08489600 0.96029400

C 2.90336600 1.97562500 -0.02453600

C 1.56545800 2.07781300 -0.41066200

C -4.37920000 -0.55200900 0.55291600

C -5.64854300 -1.40835200 0.48379200

C -5.96705100 -1.98292400 1.86415000

C -5.50034800 -2.54033100 -0.53615000

C 1.15194400 3.02676600 -1.52092900

C 0.95380700 4.44176400 -0.96064800

C 2.14421000 3.04374600 -2.68785800

C -0.05205400 -0.37154700 2.07568400

C 0.52067700 -1.53982100 2.87726400

C -0.82247900 0.58479000 2.99869900

C 4.78486600 0.92907100 1.29973600

C 5.05629000 1.09979900 2.79753800

C 5.30173300 -0.42504700 0.80071300

H -4.16958400 3.67958800 -0.41161000

H -4.30075300 2.75286800 1.08511600

H -5.92765900 1.50000500 -0.21686000

H -5.13069200 1.93972400 -1.73203500

H -4.30844000 -0.27502500 -1.59675700

H 2.66745900 -0.38706500 2.37498800

H 3.65135700 2.58657400 -0.52395300

H -3.51498600 -1.21470000 0.68542200

H -4.41696000 0.11135200 1.42965200

H -6.49098000 -0.77011100 0.17712700

H -6.86651400 -2.60472200 1.83621500

H -6.12285800 -1.19026100 2.60192200

H -5.13780000 -2.60845000 2.21331600

H -6.40178700 -3.15977900 -0.56051800

H -5.32376900 -2.16924800 -1.54946100

H -4.65031700 -3.17637900 -0.26788100

H 0.18595300 2.67973500 -1.90485300

H 0.20204900 4.43854900 -0.16873500

H 0.63071000 5.12665300 -1.75137600

H 1.89655300 4.81821000 -0.54853700

H 2.36875200 2.03384900 -3.04553400

H 1.72961600 3.61784100 -3.52157300

H 3.08954000 3.51818700 -2.40651600

H -0.76394800 -0.79629800 1.35998100

H 1.11334900 -2.20493300 2.24197000

H -0.29694300 -2.12025100 3.31412600

H 1.15106900 -1.19338400 3.70322600

H -1.26743200 1.41254800 2.43948200

H -1.61869300 0.04781700 3.52482500

H -0.14565800 1.01009200 3.74747100

H 5.32801700 1.71966000 0.76642700

H 4.67486600 2.05648900 3.16395100

H 6.13085600 1.05495600 2.99833000

H 4.57962100 0.30216200 3.37628000

H 5.15763300 -0.52913500 -0.27817300

H 6.36718200 -0.54368900 1.02127800

H 4.76030300 -1.24616000 1.28294900

C -1.17701800 -0.95907200 -1.11426700

O -2.25499100 -1.83584800 -1.31416300

C 0.16751200 -1.53076000 -1.17092000

C 0.40669300 -2.81191200 -0.64931000

C 1.24235700 -0.84873400 -1.76173600

C 1.67895300 -3.37003200 -0.66698500

H -0.42143700 -3.36012200 -0.20948500

C 2.52399700 -1.38279200 -1.76343000

H 1.06367900 0.11774100 -2.22283900

C 2.73276500 -2.64296800 -1.21178700

H 1.86003600 -4.35622100 -0.25320200

H 3.35046500 -0.83938500 -2.20965500

Cl 4.33537000 -3.34277100 -1.23650700

H -2.17757000 -2.19352700 -2.20753100

TS1S

C -3.97662400 -2.48012400 1.30408600

C -4.81343000 -1.17334800 1.42251200

C -4.12517200 -0.12216000 0.50891000

N -2.76269100 -0.65507200 0.47174400

C -2.63537500 -1.93053200 0.93341100

C -1.55717400 -0.15452700 0.14732900

N -0.74158300 -1.17707200 0.45695000

N -1.39054400 -2.30375800 0.93711900

C 0.69213000 -1.14094900 0.35806300

C 1.29544200 -1.72947700 -0.75590700

C 2.68163200 -1.61599300 -0.87555300

C 3.43886200 -0.91063900 0.05604500

C 2.78940700 -0.33600700 1.15176400

C 1.41380800 -0.44353100 1.34169800

C -4.73352100 -0.01492900 -0.89243900

C -5.81745300 1.06824900 -0.95661300

C -6.71802000 0.86484400 -2.17320600

C -5.17394800 2.45826300 -0.98289200

C 0.71880600 0.13708300 2.56565200

C 0.28923200 -0.99385200 3.51538900

C 1.57967800 1.13286600 3.34290500

C 0.47675700 -2.47667200 -1.79361600

C 0.99727800 -2.27469500 -3.21895900

C 0.41802500 -3.96954000 -1.44223100

C 4.94726400 -0.79487800 -0.06085100

C 5.61602000 -1.99225400 0.62815300

C 5.43756200 -0.66101200 -1.50333200

H -3.95440800 -3.05664000 2.22908200

H -4.34601000 -3.13253400 0.50653700

H -5.85850400 -1.32156000 1.14186000

H -4.78762300 -0.82690600 2.45840700

H -4.06463500 0.86494400 0.97439600

H 3.17320800 -2.06751000 -1.73185900

H 3.38495600 0.22449600 1.86683500

H -3.94437900 0.23396400 -1.61399800

H -5.13580600 -0.99369900 -1.18938800

H -6.43801200 0.98369400 -0.05132800

H -7.46899400 1.65677200 -2.24402400

H -7.23864700 -0.09692500 -2.13240300

H -6.12317400 0.88725400 -3.09343000

H -5.93030000 3.24103100 -0.87549600

H -4.41985700 2.59524700 -0.19907100

H -4.67498600 2.60978900 -1.94782000

H -0.17920200 0.67028700 2.22387100

H -0.35763700 -1.72516300 3.02789300

H -0.24076700 -0.57809400 4.37787700

H 1.17518700 -1.52195100 3.88370300

H 1.98018200 1.91780700 2.69836800

H 0.97511600 1.60391900 4.12255800

H 2.41768400 0.62849900 3.83642600

H -0.54575100 -2.08189300 -1.75697100

H 1.13426500 -1.21405700 -3.45082800

H 0.28919000 -2.69620800 -3.93776600

H 1.95533400 -2.78048400 -3.37345300

H -0.01672800 -4.11798400 -0.45107100

H -0.18347200 -4.51433500 -2.17681100

H 1.42745100 -4.39478500 -1.44487700

H 5.23836300 0.11597900 0.47692000

H 5.30951200 -2.06782500 1.67498800

H 6.70604800 -1.90272200 0.59068800

H 5.33145200 -2.92319900 0.12610100

H 4.91739000 0.14602800 -2.02629000

H 6.50862600 -0.44022100 -1.51623600

H 5.29073900 -1.59016800 -2.06396500

C 1.94782500 1.75928700 -1.53826400

C 0.59241100 1.51398700 -1.37449300

C -0.11603400 2.08936800 -0.31999800

C 0.53078400 2.95586200 0.55137500

C 1.88958900 3.22757800 0.39478500

C 2.58405100 2.60884300 -0.63580000

H 2.51392200 1.28765100 -2.33451600

H 0.07812000 0.83024400 -2.04911300

H -0.04927700 3.39862400 1.35596100

H 2.41072300 3.89756900 1.07065700

C -1.58828500 1.79942400 -0.11217700

O -2.19472200 2.26304000 0.87761800

H -2.11670000 1.70131600 -1.08853500

Cl 4.30527900 2.88197300 -0.79352800

M1S

C 4.00270300 -2.38698800 1.21490700

C 4.80810900 -1.67816600 0.08750600

C 4.09651700 -0.32119400 -0.19138600

N 2.76372300 -0.60911500 0.33594700

C 2.64902700 -1.78340700 1.01768100

C 1.53344200 -0.14814000 0.06347000

N 0.71898900 -1.03472800 0.65381600

N 1.40655000 -2.08522000 1.23885200

C -0.70286100 -1.12322500 0.44863700

C -1.56528100 -0.44643700 1.32404600

C -2.92571900 -0.50841600 1.04380200

C -3.42056700 -1.22677400 -0.04956800

C -2.52392200 -1.89967400 -0.87221400

C -1.14383900 -1.85947400 -0.65343500

C 4.73586500 0.87445600 0.51203500

C 5.97977900 1.40676000 -0.21009100

C 6.75045600 2.35812400 0.70457500

C 5.60458400 2.11036400 -1.51610900

C -0.19176900 -2.65072200 -1.53631500

C -0.27339100 -4.13329600 -1.14988400

C -0.44279500 -2.43535200 -3.02956000

C -1.02731800 0.27122800 2.55010500

C -2.00538700 1.28758400 3.13810200

C -0.62934400 -0.75208800 3.62554500

C -4.91323600 -1.28783300 -0.31098800

C -5.62929200 -2.06542900 0.79964400

C -5.51641000 0.11089900 -0.47220900

H 3.99116800 -3.47195000 1.11312600

H 4.38636900 -2.13983500 2.20965200

H 5.85444200 -1.52863400 0.36145900

H 4.77819700 -2.29343700 -0.81432800

H 3.96279500 -0.14060800 -1.26308100

H -3.62371400 0.01703200 1.68837100

H -2.90859500 -2.47042700 -1.71387100

H 3.99595500 1.68257300 0.58572000

H 4.98891100 0.58062600 1.54047300

H 6.63486300 0.55548600 -0.44842300

H 7.63882800 2.75195600 0.20319400

H 7.07090200 1.85914100 1.62402800

H 6.12039200 3.20962400 0.98507600

H 6.50000300 2.46193000 -2.03632300

H 5.05365300 1.46279100 -2.20351400

H 4.97475900 2.98218300 -1.30606600

H 0.82457200 -2.28986200 -1.35937800

H -0.04114700 -4.27467300 -0.09031100

H 0.43257800 -4.72139500 -1.74450400

H -1.27913100 -4.52644000 -1.33403800

H -0.20806100 -1.40085800 -3.28752300

H 0.22442500 -3.08340200 -3.60634400

H -1.46923600 -2.67811400 -3.32266900

H -0.12449200 0.81352200 2.24147100

H -2.38814400 1.97001500 2.37497500

H -1.50522800 1.87538000 3.91285200

H -2.85690800 0.78619600 3.61009900

H 0.10600500 -1.46896000 3.25372000

H -0.21243800 -0.24261300 4.50004000

H -1.51323500 -1.31235600 3.94767700

H -5.05706000 -1.83144500 -1.25310200

H -5.22009700 -3.07386200 0.90310200

H -6.69970200 -2.14430100 0.58716700

H -5.51380300 -1.55548400 1.76178000

H -5.01299500 0.67040300 -1.26424600

H -6.58080600 0.04417000 -0.71663700

H -5.42130800 0.69123300 0.45094700

C -1.43305200 3.18488700 0.20385800

C -0.21269800 2.53520600 0.04774500

C -0.03729600 1.57628400 -0.94757600

C -1.08202600 1.29768200 -1.82083500

C -2.31123500 1.93400100 -1.68005600

C -2.47706800 2.86073800 -0.65672300

H -1.57973600 3.93336800 0.97522200

H 0.61545500 2.78078100 0.71194700

H -0.90374600 0.56515800 -2.60110300

H -3.13400500 1.71901000 -2.35363900

C 1.28778900 0.82798100 -1.11689400

O 1.41771200 0.02251300 -2.15522600

H 2.09184700 1.60487300 -0.98264600

Cl -4.02715600 3.64571000 -0.44739700

TS2S

C 2.59985200 -2.56472100 2.45405400

C 3.70249600 -2.37859400 1.38245100

C 3.36016600 -1.07891400 0.61968600

N 1.88195300 -1.06849300 0.78055100

C 1.44866100 -1.90171900 1.77211800

C 0.79458100 -0.59682200 0.13471800

N -0.23910200 -1.13562400 0.80633200

N 0.15195400 -1.96680900 1.82455600

C -1.63909700 -0.96316900 0.53586700

C -2.30128500 0.12886700 1.10463900

C -3.67281500 0.23611400 0.87671100

C -4.36337000 -0.70310100 0.11104100

C -3.65494300 -1.76777300 -0.44100000

C -2.28182500 -1.92193600 -0.25111000

C 4.01196900 0.18477500 1.17097200

C 5.44969900 0.32823800 0.65108900

C 6.21968500 1.35380300 1.48198200

C 5.44928800 0.71761900 -0.83090600

C -1.53409800 -3.08734500 -0.86201400

C -1.83489900 -4.37283000 -0.08297600

C -1.84516900 -3.24516100 -2.35192100

C -1.58448300 1.12992600 1.99138000

C -1.99778900 2.57305100 1.69650000

C -1.80059900 0.76606100 3.46553500

C -5.85687500 -0.56602000 -0.11848600

C -6.63472000 -0.65225200 1.19944000

C -6.19249000 0.72989000 -0.86370700

H 3.58073200 -1.19673400 -0.44214200

H -4.21254600 1.07369000 1.31225800

H -4.18383000 -2.49784100 -1.04992500

H 3.44970000 1.06419500 0.83670100

H 3.98946000 0.18001600 2.27162300

H 5.95117400 -0.64503700 0.75509000

H 7.23947500 1.48000200 1.10500300

H 6.27772600 1.06080300 2.53577500

H 5.72369400 2.33040200 1.43087900

H 6.47102500 0.72450100 -1.22463200

H 4.83759700 0.04943300 -1.44755900

H 5.04198600 1.73041800 -0.94275000

H -0.45965400 -2.89386200 -0.81248000

H -1.54673800 -4.26951100 0.96775000

H -1.27689100 -5.20915600 -0.51491300

H -2.90393200 -4.61666700 -0.12265600

H -1.74790400 -2.28131100 -2.85675700

H -1.11421700 -3.92674000 -2.79317300

H -2.85430000 -3.64179300 -2.52277500

H -0.51266100 1.05820600 1.78384800

H -1.88841100 2.79884500 0.63158300

H -1.36220900 3.26635200 2.25616500

H -3.03522200 2.76371300 1.99212200

H -1.44265900 -0.24762700 3.66827700

H -1.26666000 1.46440000 4.11861600

H -2.86618300 0.80835200 3.71736700

H -6.16648500 -1.40906000 -0.74899100

H -6.40801300 -1.58137900 1.72913100

H -7.71384700 -0.60871700 1.01844200

H -6.37148400 0.18204300 1.85811000

H -5.64956100 0.78839300 -1.81027000

H -7.26600300 0.79173800 -1.07088900

H -5.91475300 1.60347100 -0.26503600

C 0.85583700 3.76013400 0.12718700

C 1.04798800 2.38575500 0.04979900

C 0.48611400 1.61810300 -0.98292000

C -0.30716300 2.28469200 -1.92892000

C -0.51136200 3.65829900 -1.86178000

C 0.07633400 4.38910500 -0.83565500

H 1.29452200 4.33828100 0.93383300

H 1.63085500 1.89905400 0.82762800

H -0.76073700 1.68638700 -2.71041600

H -1.12786600 4.16396500 -2.59787900

C 0.72094300 0.16054800 -1.15484000

O -0.22991900 -0.45300100 -1.97752900

H 1.83811700 -0.16282900 -1.71489500

Cl -0.18977900 6.12408700 -0.73768000

C 2.41837300 -2.19023200 -2.05888900

O 3.24658000 -3.03920400 -1.66479000

O 2.79721600 -0.92788600 -2.25785500

O 1.15104200 -2.45619700 -2.21251100

H 0.32039200 -1.32764900 -2.26967700

H 2.83324400 -2.04873700 3.39227800

H 2.38764300 -3.61033000 2.67942000

H 4.70459800 -2.33865200 1.81650400

H 3.65006200 -3.18208700 0.64110600

M2′

C 1.05455800 -3.63280800 1.89870700

C 2.49233000 -3.25205900 1.48524900

C 2.34582100 -2.18668800 0.37776800

N 1.11085800 -1.49167800 0.83094800

C 0.26987300 -2.48377100 1.35004800

C 0.25717100 -0.64726300 0.08550900

N -1.02331200 -1.18602300 0.29408900

N -0.97203400 -2.33988100 1.08237600

C -2.26647700 -0.51203900 0.13168900

C -2.54044000 0.65825000 0.86071200

C -3.75358300 1.30266100 0.62814500

C -4.69891000 0.80554300 -0.26956400

C -4.40687100 -0.37241400 -0.95252400

C -3.19610400 -1.04465700 -0.77860700

C 3.58559000 -1.32143000 0.22330200

C 4.87424200 -2.10865000 -0.04986800

C 6.02807900 -1.11861500 -0.21670800

C 4.75714600 -3.00364100 -1.28512900

C -2.91438000 -2.33815500 -1.52263700

C -3.57806700 -3.51487300 -0.79469900

C -3.35115600 -2.28827600 -2.98938900

C -1.57694200 1.22585000 1.88816700

C -1.04988200 2.59617700 1.44461900

C -2.23389300 1.30751000 3.27107000

C -6.01610600 1.52529900 -0.48654800

C -6.84954800 1.55484200 0.79947600

C -5.79771100 2.94232800 -1.02751600

H 0.71364500 -4.55840800 1.42926900

H 0.93909400 -3.74176600 2.97909600

H 3.02086600 -2.79805700 2.32977600

H 3.07709100 -4.10840900 1.14407100

H 2.11442000 -2.67369900 -0.58316100

H -3.97163600 2.21774300 1.17524400

H -5.14309600 -0.77132900 -1.64606200

H 3.44141900 -0.61160900 -0.59791700

H 3.71171600 -0.72959200 1.13766200

H 5.09290400 -2.74447400 0.81958400

H 6.97920000 -1.63828700 -0.36366900

H 6.12385400 -0.46831900 0.65818200

H 5.85403100 -0.47620000 -1.08745200

H 5.71492400 -3.48323900 -1.50719000

H 4.01331300 -3.79650100 -1.15792500

H 4.46877800 -2.40993600 -2.16025200

H -1.83150300 -2.50128600 -1.49726100

H -3.21335100 -3.58396300 0.23244400

H -3.36127700 -4.45630700 -1.30956500

H -4.66518700 -3.38124000 -0.77236100

H -2.93290600 -1.41921100 -3.50621400

H -3.01525100 -3.19000700 -3.50933400

H -4.44044500 -2.24583700 -3.08597100

H -0.72628100 0.54323100 1.97348400

H -0.63197900 2.55056400 0.43610700

H -0.27440700 2.95036100 2.13127800

H -1.85857100 3.33572100 1.44450800

H -2.59197500 0.32700700 3.59584200

H -1.51435300 1.67610200 4.00845500

H -3.08590500 1.99486000 3.26673200

H -6.57680700 0.95799700 -1.24018200

H -7.02319600 0.54503600 1.18030600

H -7.81864500 2.03017400 0.62064200

H -6.33602400 2.12436000 1.58103500

H -5.21749800 2.92711100 -1.95364000

H -6.75680600 3.42992100 -1.22651400

H -5.25468400 3.55648700 -0.30180900

C 3.52971100 2.25736100 0.72891900

C 2.36543500 1.50620400 0.64288900

C 1.76629100 1.23718200 -0.59556000

C 2.31672800 1.82279500 -1.74363500

C 3.47759500 2.58248500 -1.67079900

C 4.08632400 2.77870100 -0.43482000

H 3.99979600 2.45159300 1.68693000

H 1.91857600 1.09665900 1.54535300

H 1.82889500 1.66292000 -2.69924500

H 3.91517400 3.01819400 -2.56224600

C 0.54926400 0.43002600 -0.68602800

O -0.38712900 0.98012100 -1.55216200

Cl 5.56010500 3.71150300 -0.33959700

H -1.07576200 0.33092600 -1.74290500

t-TS3S

C -3.67942300 1.32772600 -2.41959000

C -4.49178700 1.28704000 -1.10194500

C -3.47185700 1.53190200 0.04085100

N -2.25325500 0.98355600 -0.57991500

C -2.31901700 0.93825700 -1.94043200

C -0.95438800 0.76464200 -0.20257500

N -0.31515900 0.56032200 -1.40444400

N -1.18031900 0.66524900 -2.48891700

C 1.08815500 0.52986400 -1.68908200

C 1.86295200 1.68141500 -1.46879400

C 3.23011300 1.58591400 -1.71079400

C 3.82119600 0.41140100 -2.18205800

C 3.01057000 -0.69546800 -2.41525800

C 1.63947300 -0.66644800 -2.15912700

C -3.27927700 3.00715400 0.41827100

C -4.09163400 3.40779700 1.65298100

C -5.59275600 3.17933000 1.46956800

C -3.81043100 4.86412500 2.01751300

C 0.78648800 -1.91005400 -2.33694600

C 0.35897100 -2.07734400 -3.80027200

C 1.48077200 -3.17350700 -1.81702700

C 1.23050400 2.98655700 -1.01123400

C 2.21498700 3.93026100 -0.32106500

C 0.55096000 3.67999200 -2.20131600

C 5.32163700 0.32823400 -2.38078400

C 5.84584000 1.43226600 -3.30408200

C 6.03868500 0.35909000 -1.02558000

H -4.06310400 0.65836700 -3.19069900

H -3.62876700 2.33618900 -2.84305000

H -5.28742700 2.03336400 -1.08663500

H -4.93149500 0.29632800 -0.96434800

H -3.71867000 0.93368900 0.91725100

H 3.85981300 2.44805100 -1.51483100

H 3.46730500 -1.61249500 -2.77882800

H -2.22221800 3.17265600 0.64213900

H -3.54911400 3.64706800 -0.43617200

H -3.73515700 2.76950900 2.47317400

H -6.14330500 3.48694800 2.36342600

H -5.83200600 2.12767100 1.28154600

H -5.97216900 3.77028800 0.62631600

H -4.34660200 5.15290200 2.92632200

H -2.74236900 5.02909100 2.18571400

H -4.13489100 5.53327000 1.21183800

H -0.12714300 -1.75466500 -1.74532500

H -0.17986800 -1.19188900 -4.14580400

H -0.29068900 -2.95279400 -3.91385700

H 1.23855000 -2.22215900 -4.43679000

H 1.81921000 -3.05578400 -0.78187700

H 0.79608900 -4.02749700 -1.85746600

H 2.34777000 -3.43456700 -2.43094500

H 0.45876600 2.74906300 -0.27218300

H 2.74328400 3.42318300 0.49193000

H 1.67098100 4.77985000 0.10071600

H 2.95232700 4.33291600 -1.02352400

H -0.18024900 3.02363100 -2.68169200

H 0.04190700 4.59118300 -1.87182600

H 1.29844400 3.95792000 -2.95192100

H 5.53344100 -0.63787400 -2.85642900

H 5.32977300 1.42195600 -4.26772400

H 6.91704700 1.30183300 -3.48327600

H 5.70451200 2.42067100 -2.85545500

H 5.69512000 -0.45103500 -0.37660200

H 7.12093500 0.26341000 -1.15664600

H 5.84169600 1.30208300 -0.50469400

C -0.50006800 0.83321300 1.14128600

O -1.31172800 1.40353800 1.99554100

H -2.03982500 0.36649100 2.48606700

C -3.28813500 -2.13177000 -0.05243000

O -4.24596700 -1.48799100 0.35633300

C -3.44883700 -3.06168100 -1.27504300

C -2.31063300 -4.07052000 -1.45449200

C -4.76727900 -3.82066900 -1.07217300

C -3.55281300 -2.18002800 -2.52589800

H -1.36430800 -3.58509000 -1.71969100

H -2.16157600 -4.67953200 -0.55636200

H -2.55494900 -4.74636200 -2.27933200

H -5.58653100 -3.11853400 -0.91224300

H -4.98246000 -4.43130000 -1.95435800

H -4.70923300 -4.48128800 -0.20162900

H -3.74645000 -2.80170400 -3.40622200

H -4.38049600 -1.47302900 -2.41651200

H -2.62703600 -1.61892400 -2.70195900

N -2.04522100 -2.10005000 0.52143800

H -1.32393800 -2.58702200 0.01166200

C -1.63024300 -1.57151700 1.75490500

C -2.49019400 -0.79942700 2.59716200

H -2.37067600 -1.02890400 3.65520200

H -3.52884600 -0.76471600 2.28524700

C 0.91277800 0.62126800 1.50657500

C 1.48072700 1.45730200 2.47536700

C 1.69809400 -0.41725800 0.98991500

C 2.80237400 1.29403700 2.87897800

H 0.86212100 2.23761000 2.90764300

C 3.02103300 -0.58581300 1.36553300

H 1.25098200 -1.13295800 0.31055100

C 3.56490900 0.27963600 2.31002800

H 3.24277100 1.94752600 3.62427300

H 3.61092100 -1.40355600 0.96453200

C -0.40292200 -2.16021300 2.29979200

C 0.22969700 -3.28227300 1.73495400

C 0.20338600 -1.57799200 3.43185000

C 1.41512900 -3.78484300 2.26430800

H -0.21084900 -3.81392700 0.89485400

C 1.37902700 -2.08623900 3.96110200

H -0.22916600 -0.67922400 3.85975100

C 1.99993400 -3.19093800 3.37793900

H 1.87363700 -4.65595500 1.80568700

H 1.83194800 -1.59802500 4.81826700

H 2.92804300 -3.57840500 3.78468700

Cl 5.22607500 0.06294400 2.81592400

t-M3S

C -3.10216400 2.52393200 -2.65440500

C -4.18130000 2.10741500 -1.62264300

C -3.41587500 1.83149500 -0.30708200

N -2.14723100 1.31987100 -0.86920800

C -1.90322700 1.81806000 -2.11121200

C -1.03520200 0.70594500 -0.43655900

N -0.12455000 0.95705000 -1.40064400

N -0.67083600 1.63520200 -2.47026200

C 1.31496400 1.01427600 -1.27282600

C 1.85513000 1.92501500 -0.34156000

C 3.24243200 1.96933100 -0.24374700

C 4.07384500 1.19142700 -1.05366300

C 3.48951800 0.33946800 -1.98217400

C 2.10354200 0.22266300 -2.10743500

C -3.12927800 3.07375300 0.54814900

C -4.11269200 3.22065700 1.71175800

C -5.56602200 3.33194100 1.24966600

C -3.72644500 4.42484000 2.56852900

C 1.49871500 -0.72788100 -3.12318000

C 1.35690500 -0.03838800 -4.48800900

C 2.32200800 -2.01173600 -3.27156700

C 0.98756000 2.85455900 0.50178500

C 1.72376900 3.44532100 1.70530600

C 0.43104100 4.00160600 -0.35773100

C 5.57942500 1.24161500 -0.89109100

C 6.12849100 2.66950800 -0.96473400

C 5.98310000 0.56315600 0.42207800

H -3.33850700 2.21318400 -3.67252900

H -2.92464300 3.60404800 -2.65664300

H -4.94807900 2.87141000 -1.48772000

H -4.65837000 1.18285200 -1.96087100

H -3.88390400 1.06863700 0.30550300

H 3.69520500 2.63280300 0.48513400

H 4.13396100 -0.26385800 -2.61505000

H -2.13122300 2.95488400 0.97606100

H -3.14230400 3.97705900 -0.08221700

H -3.99580900 2.31127700 2.31700000

H -6.23790400 3.45212300 2.10464700

H -5.89495900 2.44481300 0.69790700

H -5.69853400 4.20493700 0.59784600

H -4.38426500 4.51674300 3.43780000

H -2.69740500 4.33504900 2.92860200

H -3.80523800 5.35290400 1.98981200

H 0.49182000 -0.99125500 -2.76995000

H 0.72873500 0.85063800 -4.41474000

H 0.91543800 -0.72417600 -5.21874000

H 2.34482400 0.25923100 -4.85618700

H 2.54988400 -2.46593000 -2.30127300

H 1.78088400 -2.74316700 -3.87920500

H 3.27063200 -1.81639300 -3.78039400

H 0.14432900 2.28372900 0.90907000

H 2.21458800 2.66939100 2.29773300

H 1.00516200 3.96412700 2.34602100

H 2.47621500 4.17840100 1.39423400

H -0.13827500 3.64354700 -1.21815600

H -0.22027400 4.64003100 0.24783800

H 1.25418700 4.61578500 -0.73806200

H 6.01560300 0.66985900 -1.72008200

H 5.82396700 3.16578800 -1.88994000

H 7.22140300 2.65770700 -0.92122100

H 5.77311400 3.27318100 -0.12361300

H 5.62446800 -0.46905500 0.45800800

H 7.07065600 0.55912800 0.54355800

H 5.54559300 1.08841600 1.27775200

C -1.02719900 -0.03732000 0.93505400

O -1.69766900 0.76133100 1.75879100

H -3.61894400 -0.69163800 1.46204500

C -1.71808300 -2.14191000 -1.73473500

O -2.33817100 -1.18564500 -2.18842800

C -1.49585100 -3.39770800 -2.60025600

C -0.30565800 -4.25588000 -2.15707500

C -2.79516600 -4.21118000 -2.45527900

C -1.33030500 -2.97104200 -4.06008800

H 0.61020200 -3.66489000 -2.05947500

H -0.49250400 -4.77071900 -1.20782300

H -0.11989900 -5.03377900 -2.90373800

H -3.65102700 -3.62606400 -2.80215100

H -2.73167200 -5.12760300 -3.05079100

H -2.96938700 -4.49113500 -1.41070600

H -1.32803700 -3.85309900 -4.70786200

H -2.14753000 -2.31083400 -4.35339500

H -0.39108000 -2.43227000 -4.21335100

N -1.27990400 -2.21982700 -0.44840600

H -0.67428200 -2.99508700 -0.23193200

C -1.77546200 -1.47455500 0.72213400

C -3.29514500 -1.29454200 0.61240300

H -3.76968500 -2.27895200 0.66544000

H -3.58380200 -0.82891900 -0.32960200

C 0.43476500 -0.33122100 1.39171600

C 0.83221400 0.26581500 2.58550100

C 1.34064500 -1.17736900 0.74315500

C 2.08861700 0.02801400 3.13536200

H 0.11148500 0.91788800 3.07050500

C 2.60594400 -1.42072300 1.26942100

H 1.08191200 -1.63532500 -0.20686000

C 2.96419700 -0.82049600 2.47157900

H 2.38538900 0.48531100 4.07363900

H 3.30586400 -2.07304000 0.75748200

C -1.56499300 -2.35948700 1.96489000

C -1.47602400 -3.75192600 1.87419200

C -1.58443900 -1.78570000 3.24369600

C -1.35020800 -4.54496200 3.01398600

H -1.54382200 -4.25444200 0.91324800

C -1.45665200 -2.57744200 4.37831400

H -1.70158500 -0.70949700 3.31902500

C -1.32806100 -3.96038200 4.27286000

H -1.27843900 -5.62315300 2.90861400

H -1.46111600 -2.10653700 5.35674700

H -1.22753900 -4.57421700 5.16232200

Cl 4.52768500 -1.16524500 3.17826700

t-TS4S

C -2.87730200 -3.64518700 0.99004900

C -4.00535100 -2.78099400 0.36970400

C -3.30697100 -1.76811600 -0.56863200

N -2.01222600 -1.62669600 0.12085000

C -1.71096100 -2.71765400 0.88490500

C -0.92059000 -0.84378700 -0.01499000

N 0.01479300 -1.55236400 0.65904200

N -0.46553700 -2.71352200 1.24532900

C 1.44874500 -1.42467900 0.57754500

C 2.07163900 -1.71370700 -0.64743800

C 3.46462800 -1.61838600 -0.68811200

C 4.22189100 -1.27260600 0.43025700

C 3.55273200 -0.99825700 1.62307800

C 2.16561300 -1.05923600 1.72156700

C -3.07246600 -2.24031800 -2.01022700

C -4.11459600 -1.69361000 -2.98941700

C -5.53925000 -2.11244400 -2.62681000

C -3.76616500 -2.12508000 -4.41228100

C 1.45321200 -0.72512200 3.01791600

C 1.39995300 -1.94488300 3.94616900

C 2.06951900 0.47624400 3.73887600

C 1.27886600 -2.12580400 -1.88566400

C 2.08175000 -2.02729400 -3.18403300

C 0.73643200 -3.55749400 -1.74721400

C 5.74057500 -1.20229200 0.40512300

C 6.33388400 -2.32125300 1.27352700

C 6.34224100 -1.25450900 -0.99731500

H -3.08349400 -3.94374900 2.01917700

H -2.67671600 -4.55055900 0.40830500

H -4.74256000 -3.38120100 -0.16582600

H -4.50973900 -2.21690500 1.15883100

H -3.82783200 -0.81764000 -0.54061400

H 3.96950500 -1.81456300 -1.62601400

H 4.13555800 -0.71030600 2.49505200

H -2.09368800 -1.87336700 -2.33544500

H -3.05415700 -3.34108400 -2.04675300

H -4.03579200 -0.59915700 -2.93641200

H -6.25731300 -1.71757200 -3.35157000

H -5.83855900 -1.75004100 -1.63757200

H -5.63157200 -3.20598700 -2.62966600

H -4.47418400 -1.70870000 -5.13484200

H -2.76213200 -1.78748800 -4.68494200

H -3.79799500 -3.21732200 -4.50466900

H 0.42259200 -0.46161400 2.75159200

H 0.91984200 -2.78967700 3.44812700

H 0.84053800 -1.70684900 4.85713000

H 2.41399400 -2.24153700 4.23520300

H 2.13203400 1.35289700 3.08491900

H 1.46440500 0.74168800 4.61117900

H 3.07699000 0.25454500 4.10271600

H 0.42679100 -1.44083800 -1.98769700

H 2.54362200 -1.04441600 -3.30428100

H 1.41417200 -2.19827000 -4.03302000

H 2.86851200 -2.78850600 -3.22657300

H 1.56767100 -4.26718200 -1.67766100

H 0.11615600 -3.68765500 -0.85893700

H 0.13991000 -3.81682600 -2.62752300

H 6.01852900 -0.23891400 0.85442300

H 5.95732000 -2.28234500 2.29900700

H 7.42494300 -2.24623900 1.30638600

H 6.07005900 -3.29887800 0.85689300

H 5.91888100 -0.48415700 -1.64708600

H 7.42314000 -1.09917100 -0.94357300

H 6.17364200 -2.23304400 -1.46039900

C -1.00400500 0.79609400 -1.17399400

O -1.48518900 0.42339500 -2.26637700

H -3.27036000 2.23039000 -1.94193000

C -2.84821500 0.88131200 1.85300900

O -3.82568400 0.30619800 1.38872600

C -2.61729600 0.83298000 3.37697800

C -1.43440700 1.66572400 3.87540100

C -3.90795500 1.34268100 4.03302600

C -2.41515700 -0.64727800 3.73582400

H -0.48192300 1.34247700 3.44142500

H -1.57264800 2.73284700 3.66791400

H -1.34179300 1.55557000 4.96029800

H -4.76359500 0.77021200 3.67104600

H -3.84089100 1.24072100 5.12077800

H -4.07649800 2.39865700 3.79793100

H -2.30895600 -0.76256100 4.81953400

H -3.28019000 -1.22815000 3.40392900

H -1.51987600 -1.06413000 3.26016800

N -1.93171400 1.55649000 1.11359200

H -1.16924000 1.97312200 1.62364700

C -1.92557600 1.82224500 -0.32750400

C -3.34167300 1.85123900 -0.92324600

H -3.96347800 2.52480600 -0.32836800

H -3.81542700 0.87901800 -0.95941500

C 0.48766200 1.15703700 -1.14647800

C 1.16057900 1.17391100 -2.36413200

C 1.18648200 1.50931300 0.00908400

C 2.50877900 1.51548000 -2.43738700

H 0.59978300 0.90154500 -3.25370400

C 2.53189400 1.84835400 -0.03893400

H 0.69732200 1.46583400 0.98046200

C 3.18175500 1.84149500 -1.26791700

H 3.03572800 1.52344800 -3.38582200

H 3.08276000 2.09403000 0.86253300

C -1.38617200 3.24780800 -0.54847000

C -1.31497300 4.19843600 0.46898400

C -1.03319800 3.64009000 -1.84681400

C -0.86530100 5.49391900 0.20925900

H -1.64044900 3.95290300 1.47501300

C -0.58326200 4.92634000 -2.10594200

H -1.11107800 2.91610300 -2.65355900

C -0.48876200 5.86089400 -1.07495200

H -0.81853800 6.21532400 1.01918600

H -0.30478700 5.20199700 -3.11816300

H -0.13425400 6.86650600 -1.27659300

Cl 4.88514900 2.23105900 -1.32821600

t-TS3R

C -4.23710200 -1.72827700 1.63127200

C -4.96128400 -0.89368200 0.53782200

C -3.90072000 -0.53515700 -0.54050300

N -2.66860400 -0.66197900 0.24225900

C -2.79665200 -1.44683800 1.34842300

C -1.33418000 -0.49532200 -0.01174900

N -0.72680800 -1.26127300 0.96202900

N -1.66076400 -1.83478100 1.82748800

C 0.58996900 -1.83190100 0.93140900

C 1.01883600 -2.55785600 -0.20136800

C 2.34079900 -2.99415400 -0.21398500

C 3.21402900 -2.78019600 0.85536800

C 2.72942900 -2.12267700 1.97825500

C 1.42542700 -1.62901800 2.03550800

C -3.86641300 -1.49346500 -1.73917000

C -4.69445400 -1.00783400 -2.93118500

C -6.16643500 -0.78373700 -2.58290600

C -4.56213900 -1.99335300 -4.09108600

C 0.95296600 -0.88744800 3.26887700

C 0.70076800 -1.86538700 4.42160200

C 1.92277200 0.22259600 3.68878700

C 0.08138400 -2.90518100 -1.35207800

C 0.80039600 -3.41028100 -2.60262000

C -0.96656300 -3.92900700 -0.89082500

C 4.66316400 -3.21610600 0.77331500

C 4.81095700 -4.69002900 0.38525100

C 5.42630500 -2.31166700 -0.20172800

H -4.51354800 -1.41938200 2.64159100

H -4.43162300 -2.80007700 1.53740800

H -5.79224500 -1.44374000 0.09386800

H -5.36051600 0.02118800 0.97903300

H -3.99808800 0.49391500 -0.89382500

H 2.71016500 -3.52075900 -1.08727100

H 3.39173200 -1.96808900 2.82744000

H -2.82716000 -1.58250800 -2.06926800

H -4.20306900 -2.49138400 -1.41739600

H -4.25480800 -0.04916400 -3.23925100

H -6.72532200 -0.45092400 -3.46243600

H -6.29736800 -0.02552700 -1.80374900

H -6.62695500 -1.71527700 -2.23049100

H -5.09411300 -1.63454500 -4.97698600

H -3.51298900 -2.14395700 -4.36190500

H -4.98510800 -2.96745200 -3.81835700

H -0.00329500 -0.42619800 3.02344900

H -0.04572500 -2.60786600 4.12990000

H 0.33429500 -1.33058500 5.30456600

H 1.62312500 -2.38664900 4.70006300

H 2.16528100 0.88819700 2.85235300

H 1.48311700 0.82719800 4.48979400

H 2.86475100 -0.18398800 4.06982900

H -0.44617100 -1.99781500 -1.65665100

H 1.56035600 -2.69806600 -2.93812300

H 0.07317300 -3.53614000 -3.40953500

H 1.27695000 -4.38218400 -2.43579100

H -1.53344500 -3.57320900 -0.02554700

H -1.67002200 -4.14225400 -1.70233800

H -0.47809800 -4.86670900 -0.60549700

H 5.09960200 -3.08530700 1.77176800

H 4.24804300 -5.33774300 1.06249700

H 5.86320600 -4.98751600 0.41555900

H 4.44893800 -4.86825600 -0.63216800

H 5.36454400 -1.26301700 0.10166600

H 6.48175800 -2.59644700 -0.25471600

H 5.00155600 -2.38501600 -1.20867800

C -0.83004800 0.28064800 -1.08231400

O -1.66884000 0.57880500 -2.03651000

H -2.00166700 1.86146500 -1.75383400

C 0.69162100 3.70190500 -0.74900000

O 0.44682300 3.56830200 -1.93198300

C 1.90782500 4.50545000 -0.25447400

C 2.41927300 4.06155800 1.12231500

C 3.02389200 4.36498200 -1.29210200

C 1.44957000 5.97319000 -0.18717700

H 1.73226800 4.32409400 1.93663600

H 2.60695700 2.98360400 1.14775700

H 3.36208100 4.57205000 1.33935400

H 2.65109500 4.62453700 -2.28393400

H 3.85485300 5.02854100 -1.03345500

H 3.39619600 3.33801900 -1.33020300

H 2.28477500 6.60994300 0.12179800

H 1.10142500 6.30753800 -1.16807400

H 0.63432900 6.10322900 0.53225800

N -0.14239400 3.23521500 0.24794400

H 0.17767800 3.38093700 1.19212200

C -1.42108100 2.68683500 0.11776300

C -2.18935400 2.89300000 -1.06812500

H -1.84251300 3.69819000 -1.70775500

H -3.26696100 2.90197200 -0.91399800

C 0.61596000 0.46899200 -1.29235800

C 1.10048800 0.44209800 -2.60699000

C 1.52838000 0.66946100 -0.24957400

C 2.45829800 0.54703100 -2.87203200

H 0.38171800 0.33779800 -3.41291900

C 2.89516200 0.75245100 -0.49609300

H 1.16205900 0.73793000 0.77087000

C 3.34752100 0.68474200 -1.80909500

H 2.83378400 0.52062500 -3.88896900

H 3.60348600 0.87808900 0.31756500

C -1.98364500 2.17405900 1.37124100

C -1.16325300 1.82427400 2.46660000

C -3.37297600 2.02406600 1.55058000

C -1.70009200 1.35222800 3.65624700

H -0.07972300 1.87689600 2.38273100

C -3.90578900 1.55544400 2.74519900

H -4.05449700 2.30869900 0.75669700

C -3.07708000 1.20430600 3.80789300

H -1.03247400 1.08121000 4.46963700

H -4.98478500 1.47728600 2.85079400

H -3.49352700 0.83701300 4.73964500

Cl 5.06076800 0.82623600 -2.13510400

t-M3R

C -3.86946500 -2.85223700 1.14286100

C -4.71671900 -1.67690500 0.58720600

C -3.80340900 -0.95825100 -0.43343800

N -2.49521600 -1.18897800 0.20693000

C -2.48620700 -2.30879600 0.97701400

C -1.23099100 -0.74446100 0.07030500

N -0.50130000 -1.67717800 0.71416400

N -1.27883400 -2.65385900 1.30675500

C 0.92074400 -1.90313600 0.68512300

C 1.50440500 -2.28494600 -0.53759300

C 2.88195300 -2.47623500 -0.53516500

C 3.65404600 -2.33324900 0.62277000

C 3.01831300 -1.99676000 1.81194500

C 1.64097800 -1.77313300 1.87104000

C -3.82445000 -1.57889400 -1.84014600

C -4.63440300 -0.73528500 -2.82659900

C -6.08962800 -0.56336900 -2.38919300

C -4.55892800 -1.34879000 -4.22278300

C 0.97843700 -1.42616100 3.19211000

C 0.94700200 -2.65846900 4.10432100

C 1.65915500 -0.24257000 3.88801900

C 0.66826200 -2.52405500 -1.78917100

C 1.49842200 -2.54950400 -3.07260400

C -0.11573400 -3.84035400 -1.65796800

C 5.15647400 -2.52736200 0.57449200

C 5.53739700 -3.91039200 0.03699800

C 5.81264800 -1.41687900 -0.25260700

H -4.09876800 -3.09636400 2.18066800

H -3.98011400 -3.76178900 0.54435800

H -5.64353400 -2.01368800 0.12039900

H -4.95815700 -0.99075600 1.40397800

H -3.99217100 0.11266200 -0.48449300

H 3.37647900 -2.74111100 -1.46392700

H 3.61020300 -1.90390300 2.71987700

H -2.79673400 -1.62147300 -2.20571600

H -4.23580700 -2.59905200 -1.78691100

H -4.13900000 0.24453100 -2.85342300

H -6.64573600 0.04461000 -3.10876100

H -6.17615200 -0.07420500 -1.41219400

H -6.58899400 -1.53837600 -2.32193600

H -5.09978500 -0.73737700 -4.95109300

H -3.52024500 -1.43346800 -4.55381400

H -5.00446700 -2.35086700 -4.23106600

H -0.05987100 -1.15026700 2.98932900

H 0.41182200 -3.47772900 3.61831000

H 0.44595400 -2.42380800 5.04885900

H 1.96312300 -2.99632500 4.33350800

H 1.71545000 0.63727300 3.23788200

H 1.10504700 0.03575800 4.79016100

H 2.67945300 -0.49254200 4.19434400

H -0.05335900 -1.70489800 -1.90021300

H 2.12736200 -1.65988400 -3.15950700

H 0.82803900 -2.57973500 -3.93552000

H 2.13708100 -3.43852300 -3.11983500

H -0.75306100 -3.85767000 -0.77051200

H -0.74657700 -3.98887900 -2.53986600

H 0.57663800 -4.68585300 -1.58643100

H 5.52750200 -2.45119200 1.60441700

H 5.06079200 -4.70681300 0.61459500

H 6.62132800 -4.05055500 0.08150400

H 5.23243300 -4.02185400 -1.00833300

H 5.57684800 -0.42915900 0.15231600

H 6.90037600 -1.53515300 -0.26788700

H 5.45473200 -1.43873300 -1.28722100

C -0.95038200 0.52629800 -0.81398500

O -1.62852600 0.27628500 -1.91412300

H -1.91360400 2.79286100 -1.92833900

C 0.24569200 3.65759000 -0.36559800

O 0.18598700 3.66556500 -1.58364700

C 1.16766700 4.64511100 0.38167000

C 1.55000100 4.19067800 1.79517000

C 2.43765000 4.82373500 -0.45527200

C 0.40201900 5.97624200 0.44838700

H 0.70082200 4.20153200 2.48937800

H 1.98711700 3.18591600 1.78392600

H 2.29669000 4.87644300 2.20671900

H 2.17613800 5.07177000 -1.48464900

H 3.05243400 5.62518200 -0.03289600

H 3.02848000 3.90289500 -0.47090900

H 1.01507600 6.74153300 0.93571800

H 0.15342600 6.31654000 -0.56020800

H -0.52888700 5.86862700 1.01546100

N -0.51549600 2.84818800 0.42714300

H -0.40476000 2.96168900 1.42119400

C -1.55827600 1.90752000 -0.00347700

C -2.47227300 2.59448200 -1.01787600

H -2.83563400 3.53668300 -0.59437000

H -3.31341900 1.95507000 -1.28573900

C 0.56341400 0.73028500 -1.03407000

C 0.98738600 0.82093700 -2.35531500

C 1.50492000 0.89554400 -0.01783900

C 2.31956400 1.08073300 -2.66713100

H 0.22952400 0.70301100 -3.12316400

C 2.84371800 1.13900800 -0.30200200

H 1.20278600 0.83154500 1.02410200

C 3.23404300 1.24048400 -1.63491700

H 2.64592700 1.17494400 -3.69726600

H 3.57243000 1.26307200 0.49325400

C -2.28938000 1.55057700 1.29139700

C -1.56448600 1.01884300 2.36946300

C -3.66239800 1.73457000 1.46838700

C -2.18627900 0.63630700 3.55173800

H -0.48900800 0.88180600 2.27281900

C -4.29176900 1.36381900 2.65780700

H -4.25426400 2.17717500 0.67444100

C -3.56270800 0.80058400 3.69927600

H -1.59415700 0.21305800 4.35884900

H -5.36041200 1.52411900 2.76770500

H -4.05573500 0.50822200 4.62065200

Cl 4.90050500 1.61838400 -2.01128400

t-TS4R

C -1.31180000 -4.99974600 0.12058400

C -2.73391000 -4.38827700 0.22393300

C -2.60222400 -2.94050200 -0.30784500

N -1.22099100 -2.63954200 0.12315700

C -0.46195700 -3.77393700 0.22375200

C -0.42102200 -1.54645200 0.15968000

N 0.80295600 -2.10746100 0.23879800

N 0.80160400 -3.49337800 0.29754200

C 2.05391900 -1.44203200 -0.01386100

C 2.28842100 -0.95447800 -1.31573100

C 3.50309000 -0.31371700 -1.53911500

C 4.46541600 -0.16752900 -0.53557400

C 4.19433500 -0.68354100 0.72590400

C 2.98936400 -1.33010600 1.01568300

C -2.70904500 -2.82211600 -1.83627500

C -4.09424600 -2.36263300 -2.30018800

C -5.20702600 -3.31762200 -1.86676900

C -4.09811900 -2.18497000 -3.81713600

C 2.74255200 -1.92709600 2.38767700

C 3.41256200 -3.30437400 2.48653100

C 3.21412500 -1.01094200 3.51902800

C 1.27567400 -1.13068200 -2.44307900

C 1.54470200 -0.22504400 -3.64518200

C 1.22394000 -2.59323200 -2.91251900

C 5.77639200 0.53884200 -0.82118400

C 6.56506300 -0.16956100 -1.92814800

C 5.54533300 2.01165800 -1.17431700

H -1.09887400 -5.72990500 0.90237500

H -1.14290200 -5.48188400 -0.84734100

H -3.47348000 -4.95653800 -0.34170600

H -3.04462800 -4.36698200 1.27311700

H -3.29682800 -2.24817600 0.16215900

H 3.71023800 0.09342200 -2.52342400

H 4.94355100 -0.58145200 1.50653200

H -1.97968900 -2.07992200 -2.17174000

H -2.45261600 -3.78674600 -2.30255900

H -4.25989800 -1.38183100 -1.83622300

H -6.17830000 -2.96851800 -2.22893600

H -5.27859000 -3.40511300 -0.77729300

H -5.04012000 -4.32120600 -2.27861200

H -5.06486000 -1.81136500 -4.16660700

H -3.32738100 -1.47396100 -4.12869200

H -3.90621300 -3.14005500 -4.32086100

H 1.66216700 -2.06517400 2.49972400

H 3.02940900 -3.97795900 1.71687300

H 3.22635900 -3.74990600 3.46907500

H 4.49617100 -3.20865700 2.35706000

H 2.78371900 -0.00914700 3.43061100

H 2.90701800 -1.42480000 4.48442200

H 4.30491400 -0.92215200 3.53677600

H 0.28585600 -0.85908600 -2.05559700

H 1.66909800 0.81870900 -3.34671100

H 0.70090100 -0.28284700 -4.33840500

H 2.44053500 -0.54138400 -4.19082300

H 0.99163500 -3.28507700 -2.10046600

H 0.46606100 -2.70860900 -3.69384500

H 2.19257000 -2.88611400 -3.33134300

H 6.37441900 0.49903900 0.09799200

H 6.73961000 -1.21976900 -1.68016500

H 7.53330200 0.31670200 -2.07944600

H 6.02235800 -0.13291200 -2.87828500

H 5.02126000 2.53681200 -0.37111800

H 6.49800000 2.51982700 -1.35234000

H 4.93819600 2.10740400 -2.08047700

C -1.45062700 0.23319900 -0.07738600

O -2.27782600 -0.06268900 -0.95442200

H -3.96798900 -0.21373700 0.71811700

C -3.43777200 2.57114300 0.48665800

O -4.45224400 1.96879900 0.18691500

C -3.32080300 4.07634300 0.15317600

C -1.99242700 4.72682100 0.55010200

C -3.52319100 4.20901300 -1.36176900

C -4.47690100 4.76874500 0.88865500

H -1.82344600 4.68522500 1.63232200

H -1.13886000 4.26846900 0.03405800

H -2.00836200 5.78335000 0.26597800

H -4.44287700 3.70304200 -1.66138000

H -3.58518300 5.26595800 -1.64072000

H -2.68554900 3.76000200 -1.90538200

H -4.50847600 5.83096300 0.62555500

H -5.42507900 4.30301600 0.61264600

H -4.35443200 4.68708400 1.97381400

N -2.38043000 2.03287000 1.17580000

H -1.54966100 2.60582500 1.15204700

C -2.07171500 0.60652900 1.36099900

C -3.35580600 -0.18616300 1.61617100

H -3.91879300 0.29608900 2.41855800

H -3.10098900 -1.19694100 1.94326600

C -0.30011200 1.16491700 -0.46234900

C -0.46712400 1.88183200 -1.64710500

C 0.82332800 1.41379800 0.32992700

C 0.45827900 2.84473900 -2.03953400

H -1.34456200 1.66201200 -2.24858300

C 1.76432400 2.36585800 -0.05296100

H 0.98382400 0.84748600 1.24210700

C 1.56684000 3.07770900 -1.23265600

H 0.32718100 3.40876800 -2.95705600

H 2.64464400 2.54940500 0.55369600

C -1.17777500 0.47258700 2.60203300

C -0.78284800 1.57949200 3.36186700

C -0.81181900 -0.79412500 3.08096900

C -0.03812400 1.43290900 4.53085800

H -1.08000700 2.57949800 3.06992900

C -0.07984900 -0.94496000 4.25306900

H -1.12002100 -1.68706100 2.54766300

C 0.31746000 0.17019900 4.98606900

H 0.24679400 2.31630000 5.09362100

H 0.17894500 -1.94321100 4.59522000

H 0.88992900 0.05279800 5.90074700

Cl 2.73031300 4.29174800 -1.71075400

c-TS3S

C -3.64657900 -2.29225400 1.84544200

C -4.49904400 -1.49729600 0.82384900

C -3.57032200 -1.23522300 -0.39118100

N -2.27208100 -1.19368800 0.28948100

C -2.26810500 -1.82834600 1.49733000

C -0.98896100 -0.86300200 -0.02360200

N -0.27192200 -1.36750600 1.02428800

N -1.07787600 -1.94103600 1.98971300

C 1.15167100 -1.47316600 1.16370800

C 1.86599900 -2.24551600 0.22745800

C 3.25297200 -2.24339600 0.33056700

C 3.92297300 -1.54921100 1.34236300

C 3.16994500 -0.85784200 2.28284100

C 1.77680700 -0.79028700 2.21114900

C -3.61773300 -2.33910700 -1.45977400

C -4.49831000 -1.95668000 -2.65302300

C -5.94231000 -1.65818200 -2.24658900

C -4.45421800 -3.05654400 -3.71145000

C 1.00275100 -0.00593600 3.25483700

C 1.03088200 -0.75123900 4.59427100

C 1.51391700 1.43012600 3.40258600

C 1.15508200 -3.09718500 -0.81611400

C 2.07146300 -3.57436700 -1.94212500

C 0.48368800 -4.29909200 -0.13262400

C 5.43735300 -1.50937100 1.38273700

C 6.06981700 -2.90055200 1.28722000

C 5.95858300 -0.59054300 0.27136500

H -3.91383300 -2.09125500 2.88390200

H -3.71981700 -3.37197100 1.68108900

H -5.40106500 -2.03396900 0.52688300

H -4.80052500 -0.54490200 1.27007400

H -3.75612400 -0.26082000 -0.84783400

H 3.83502100 -2.79635800 -0.39877200

H 3.68208800 -0.33308000 3.08633900

H -2.60260600 -2.50722300 -1.82568700

H -3.98175500 -3.27474000 -1.00885900

H -4.05719300 -1.04550100 -3.08179400

H -6.54525900 -1.39612100 -3.12070000

H -6.01252100 -0.82514100 -1.53826600

H -6.40105200 -2.53821700 -1.77872900

H -5.04205400 -2.78025800 -4.59150800

H -3.42725900 -3.24926500 -4.03390800

H -4.86676700 -3.99106900 -3.31371000

H -0.03314700 0.06483000 2.92626700

H 0.60167800 -1.75058100 4.48507200

H 0.45282100 -0.20450500 5.34673500

H 2.05587600 -0.85513400 4.96682900

H 1.46125300 1.97008900 2.45253300

H 0.89090900 1.96723000 4.12553500

H 2.54579400 1.46331300 3.76747200

H 0.37224200 -2.49589700 -1.28920300

H 2.59045400 -2.73498400 -2.41431500

H 1.47560500 -4.08173300 -2.70572000

H 2.81742000 -4.28981200 -1.57968800

H -0.21242300 -3.98544800 0.65057200

H -0.06583300 -4.89630700 -0.86721600

H 1.24083800 -4.93981300 0.33129000

H 5.72522600 -1.07401100 2.34820800

H 5.67526600 -3.57133000 2.05503900

H 7.15461000 -2.83237200 1.41115400

H 5.88068200 -3.35575500 0.30979700

H 5.54512000 0.41681900 0.36862600

H 7.05076000 -0.52352300 0.29698500

H 5.66110900 -0.97043500 -0.71219100

C -0.59060300 -0.26087500 -1.26327700

O -1.33203800 -0.59473300 -2.29919000

H -2.01533800 0.44377000 -2.56036900

C -2.19103900 1.83888200 1.27495900

O -1.05404000 1.46335500 1.53338000

C -3.15971100 2.19003500 2.41862000

C -4.59893000 2.46608000 1.97514900

C -3.13097200 1.04754400 3.43816600

C -2.57722300 3.45915300 3.06565700

H -4.66718200 3.35706000 1.34161700

H -5.03744700 1.61791300 1.43691900

H -5.22179300 2.64961700 2.85541400

H -2.10147700 0.80295900 3.71016100

H -3.68003400 1.33597700 4.33976800

H -3.59942500 0.14634100 3.03052300

H -3.17202900 3.74220600 3.93984100

H -1.54612300 3.27852100 3.37995900

H -2.58514700 4.29731300 2.36066600

N -2.61918600 2.06434600 0.00342900

H -3.59591600 2.28928100 -0.11838900

C -1.85000300 2.05965800 -1.19482600

C -2.54277800 1.62843700 -2.36308600

H -2.34033300 2.18611000 -3.27492500

H -3.60959300 1.43050300 -2.22828600

C 0.78927400 0.20209000 -1.46358100

C 1.41731800 -0.03042300 -2.69183900

C 1.47412000 0.93691800 -0.48381200

C 2.70498500 0.44000600 -2.93524800

H 0.87472300 -0.57863600 -3.45636600

C 2.76164300 1.39668300 -0.70671500

H 0.95525800 1.17913600 0.43854000

C 3.36364600 1.15063700 -1.93913400

H 3.19170800 0.26867400 -3.88939700

H 3.27898300 1.98225900 0.04634300

C -0.66874400 2.92273500 -1.28271300

C -0.23599800 3.73981400 -0.22634800

C 0.08169600 2.95059100 -2.47531900

C 0.89707300 4.53627700 -0.35382900

H -0.78517100 3.76044700 0.70658900

C 1.21069800 3.74548200 -2.59871700

H -0.20137600 2.30552500 -3.30052200

C 1.63246500 4.54192700 -1.53526000

H 1.20571800 5.15611300 0.48271600

H 1.77983400 3.72504800 -3.52310600

H 2.52543600 5.15195800 -1.62582200

Cl 4.95622600 1.80251200 -2.26450700

t-M3S

C -3.53013600 -2.80914500 1.40664800

C -4.46077000 -1.84100700 0.63190600

C -3.57802000 -1.23352800 -0.48627200

N -2.27501600 -1.21263600 0.20562600

C -2.19374600 -2.18868000 1.15336700

C -1.03383800 -0.72267300 -0.01208300

N -0.25417800 -1.47860000 0.78503900

N -0.97215600 -2.38469300 1.53572000

C 1.18160500 -1.54632000 0.89313800

C 1.90069800 -2.09509700 -0.18457200

C 3.28593100 -2.11289100 -0.06451500

C 3.93881300 -1.65842200 1.08645500

C 3.17393000 -1.18972800 2.14678000

C 1.78000000 -1.12059500 2.07871100

C -3.50470800 -2.12366500 -1.73893900

C -4.38627200 -1.61830100 -2.88318000

C -5.85205000 -1.45947500 -2.47734400

C -4.25943300 -2.55291600 -4.08436300

C 0.98315600 -0.64016100 3.27746300

C 1.02141000 -1.70116300 4.38365700

C 1.46853100 0.71782700 3.79031700

C 1.19493200 -2.70170800 -1.39071200

C 2.11860300 -2.90917100 -2.59096600

C 0.55917000 -4.04701400 -1.00189400

C 5.45224600 -1.66569400 1.16719600

C 6.03937900 -3.05406000 0.89494100

C 6.03692100 -0.62350700 0.20843800

H -3.76474000 -2.87500200 2.47049900

H -3.55277800 -3.82079700 0.99074100

H -5.33295100 -2.34491100 0.21293300

H -4.81208900 -1.05521100 1.30718200

H -3.86786600 -0.21318600 -0.73585900

H 3.88087900 -2.49466900 -0.88786500

H 3.67462900 -0.86176000 3.05497100

H -2.47028900 -2.12604600 -2.08761400

H -3.80066900 -3.14894600 -1.46786000

H -3.98093400 -0.63913700 -3.17105800

H -6.45170900 -1.09226500 -3.31534300

H -5.98168000 -0.75465800 -1.64801000

H -6.27263200 -2.42452500 -2.16726300

H -4.83980800 -2.18276800 -4.93465200

H -3.21596100 -2.64552800 -4.39714600

H -4.63228300 -3.55357600 -3.83532700

H -0.05360000 -0.50664900 2.96779200

H 0.62224500 -2.65118200 4.01888700

H 0.42397600 -1.37767500 5.24243300

H 2.04684600 -1.86954800 4.73038900

H 1.44043200 1.47038300 2.99838400

H 0.81582700 1.05969400 4.60035000

H 2.48756300 0.66214800 4.18721600

H 0.39449000 -2.02509500 -1.71615400

H 2.65623400 -1.99323200 -2.84854700

H 1.52458000 -3.21287700 -3.45721300

H 2.85160500 -3.70099800 -2.40022700

H -0.14181800 -3.94923600 -0.16947600

H 0.02290700 -4.46877000 -1.85787800

H 1.33565700 -4.75840900 -0.70078800

H 5.72450000 -1.37919900 2.19094900

H 5.61738600 -3.80327300 1.57000300

H 7.12522500 -3.04050600 1.02740800

H 5.83637700 -3.37162700 -0.13269900

H 5.65087900 0.37505800 0.42948900

H 7.12896200 -0.59838700 0.27737600

H 5.76649800 -0.85550500 -0.82733900

C -0.74848300 0.28864900 -1.16442200

O -1.23140300 -0.33267800 -2.22834700

H -1.97124800 1.77729300 -3.03025500

C -2.09195800 1.54282700 1.55855200

O -0.94496600 1.23492600 1.87397200

C -3.15383400 1.73042500 2.65611700

C -4.56985800 1.97098700 2.12819800

C -3.12248600 0.47187500 3.53290000

C -2.70198200 2.93835000 3.49241500

H -4.64673800 2.91896400 1.58414100

H -4.90562900 1.16121200 1.47034700

H -5.26944900 2.02573700 2.96714600

H -2.10979400 0.29386800 3.90233700

H -3.79814900 0.59122100 4.38541900

H -3.43914800 -0.41156100 2.96756700

H -3.36499900 3.06581900 4.35389300

H -1.68050000 2.78572000 3.84937200

H -2.73152400 3.85998000 2.90150700

N -2.45997900 1.74038000 0.27504800

H -3.39669200 2.07735800 0.10748900

C -1.57126500 1.76360800 -0.90006700

C -2.51483600 1.91841400 -2.09945300

H -2.97175200 2.91371000 -2.08031400

H -3.28858600 1.15025500 -2.08601800

C 0.75794500 0.61374600 -1.25844500

C 1.36119100 0.41504400 -2.49437000

C 1.49907800 1.19684000 -0.22621800

C 2.67655600 0.81690700 -2.72297200

H 0.75807800 -0.03759900 -3.27633100

C 2.81397000 1.59201200 -0.42982800

H 1.02365700 1.37168000 0.73426200

C 3.38378500 1.41406000 -1.68944400

H 3.14303000 0.68592700 -3.69383900

H 3.38415100 2.06119800 0.36512500

C -0.66658000 3.00315300 -0.87540300

C -0.51231300 3.82492200 0.24118200

C 0.03240600 3.35538200 -2.04023400

C 0.33763300 4.93015900 0.21317300

H -1.03383100 3.60636500 1.16363100

C 0.88737600 4.44812000 -2.06892200

H -0.05783800 2.73933800 -2.92844700

C 1.05009400 5.24319100 -0.93652600

H 0.44126700 5.54252500 1.10388800

H 1.43208700 4.67627400 -2.97968400

H 1.72061100 6.09621600 -0.95444000

Cl 5.00886000 1.99516400 -1.99076200

c-TS4S

C -3.28052900 -3.44973400 0.66755700

C -4.27982000 -2.36052600 0.19402100

C -3.44780600 -1.39915900 -0.69350200

N -2.14192100 -1.50649100 -0.03315800

C -1.99059900 -2.69433500 0.62626700

C -0.95793400 -0.84911000 -0.09005800

N -0.13995400 -1.72498000 0.52579600

N -0.76269700 -2.87114300 0.99816600

C 1.29486200 -1.66975000 0.61220500

C 2.04310100 -1.90662900 -0.55697300

C 3.42909600 -1.87929100 -0.43342700

C 4.06342300 -1.65657900 0.79345700

C 3.27870600 -1.44232600 1.91955100

C 1.88325600 -1.44433000 1.85653000

C -3.34700400 -1.85972500 -2.15860500

C -4.29143700 -1.10713100 -3.09827700

C -5.75623100 -1.21205600 -2.67179000

C -4.10837900 -1.60865100 -4.52902300

C 1.05591100 -1.26370700 3.11261600

C 1.07888300 -2.54904500 3.94831400

C 1.50142600 -0.05395000 3.93634400

C 1.37073600 -2.22947400 -1.88601700

C 2.30434200 -2.08243600 -3.08808900

C 0.79675400 -3.65567900 -1.86925700

C 5.57622900 -1.64854700 0.89187600

C 6.18622200 -2.95927900 0.38458200

C 6.16160000 -0.44671000 0.14574600

H -3.50972600 -3.83838000 1.66113400

H -3.23934700 -4.29539200 -0.02586100

H -5.12682800 -2.77743400 -0.35289400

H -4.66588500 -1.82599300 1.06773700

H -3.79595200 -0.36532500 -0.63651900

H 4.04059600 -2.04080000 -1.31564000

H 3.76403100 -1.27813900 2.87891800

H -2.32343500 -1.68967200 -2.50063400

H -3.55113500 -2.94057200 -2.21213900

H -3.97762900 -0.05567500 -3.06592800

H -6.40322100 -0.66497300 -3.36382000

H -5.92870400 -0.80299200 -1.66982700

H -6.08451800 -2.25907800 -2.66909900

H -4.73680000 -1.04690300 -5.22632000

H -3.06757100 -1.50341600 -4.84745100

H -4.38504600 -2.66683100 -4.60680200

H 0.02872200 -1.07186300 2.80093500

H 0.70297400 -3.39419000 3.36604300

H 0.45596900 -2.43441600 4.84213400

H 2.09854600 -2.78089000 4.27547400

H 1.50614300 0.85285200 3.32736800

H 0.80346100 0.10299700 4.76562900

H 2.49744200 -0.19985300 4.36755000

H 0.54219300 -1.52411000 -2.02845900

H 2.81143400 -1.11492800 -3.09109500

H 1.72549100 -2.16984800 -4.01192300

H 3.06227300 -2.87355500 -3.10000300

H 0.09083700 -3.81242000 -1.05147200

H 0.28739100 -3.86589600 -2.81520900

H 1.60816800 -4.38170900 -1.75149100

H 5.83104700 -1.54770000 1.95459900

H 5.76587600 -3.82134600 0.90930400

H 7.27036700 -2.95819100 0.53171600

H 5.99729700 -3.09078800 -0.68565500

H 5.75597800 0.49246000 0.53091900

H 7.25154500 -0.42059400 0.24262800

H 5.91626400 -0.49443300 -0.92024900

C -0.92440400 0.85683100 -1.29221300

O -1.47992500 0.44861600 -2.32752100

H -2.52789000 2.75819800 -2.36645400

C -1.99283800 1.10270500 1.91578700

O -0.79473800 0.99512800 2.14455300

C -3.01111300 0.80658900 3.03636600

C -4.47746500 0.94050000 2.61722700

C -2.74281200 -0.62069000 3.52964900

C -2.70851100 1.79901000 4.16920900

H -4.72814900 1.96649600 2.32533200

H -4.73109200 0.26627800 1.79075000

H -5.12282700 0.68030500 3.46102500

H -1.71634100 -0.71192300 3.89195000

H -3.42676100 -0.86960100 4.34722600

H -2.88591500 -1.35104100 2.72519600

H -3.32107900 1.56622000 5.04598900

H -1.65331300 1.73902900 4.44558900

H -2.93081100 2.82727200 3.86353300

N -2.49439200 1.49753600 0.72144200

H -3.49360800 1.62212100 0.67849100

C -1.78279300 1.98547300 -0.46593400

C -2.92172100 2.41159900 -1.41353300

H -3.48550300 3.22516300 -0.94587400

H -3.58170300 1.56746800 -1.62595900

C 0.58147600 1.08476500 -1.32494900

C 1.17067800 1.15373100 -2.58390900

C 1.35598500 1.33262200 -0.18884600

C 2.51006900 1.50925400 -2.72514700

H 0.55310100 0.94660500 -3.45279600

C 2.69781100 1.67077500 -0.31204000

H 0.89163500 1.26914600 0.79170500

C 3.25501800 1.77537500 -1.58470600

H 2.96937600 1.59092200 -3.70447100

H 3.30275400 1.87267400 0.56552000

C -0.96995000 3.25554500 -0.18209200

C -0.89005000 3.85852500 1.07177100

C -0.34280800 3.89611100 -1.26031100

C -0.17433600 5.04165000 1.25237200

H -1.37472800 3.41008300 1.92897800

C 0.37311700 5.07143100 -1.08335500

H -0.39489600 3.45250500 -2.25043600

C 0.46575500 5.65001700 0.18085200

H -0.11912400 5.48377100 2.24235300

H 0.86088500 5.53542300 -1.93460500

H 1.02752700 6.56725000 0.32519100

Cl 4.91910400 2.28878000 -1.76266300

c-TS3R

C -3.76994800 -2.39811700 1.79124200

C -4.59426200 -1.39355900 0.94358500

C -3.73905000 -1.08012700 -0.31337900

N -2.39901000 -1.31849800 0.22535400

C -2.37795000 -2.06159000 1.36517800

C -1.12420600 -0.97184600 -0.09506200

N -0.38291000 -1.60705700 0.86781000

N -1.17639700 -2.27064100 1.79617000

C 1.03667500 -1.79268700 0.92772500

C 1.68489100 -2.45247500 -0.13537700

C 3.07280900 -2.52453900 -0.08630200

C 3.80878500 -2.01166100 0.98620700

C 3.12087300 -1.42493600 2.04098300

C 1.73003300 -1.29193300 2.03444500

C -4.02714100 -1.96729600 -1.53177400

C -4.96370100 -1.28755300 -2.53517000

C -6.28958800 -0.85410400 -1.90766900

C -5.21006100 -2.20507200 -3.73071400

C 1.03605800 -0.63166900 3.21379700

C 1.12488300 -1.54050600 4.44587800

C 1.58692500 0.76700400 3.51474700

C 0.90086000 -3.11584200 -1.25972800

C 1.75383200 -3.45333900 -2.48208400

C 0.20612300 -4.37793600 -0.72533100

C 5.32327800 -2.07433900 0.98438700

C 5.84414600 -3.49873000 0.76988700

C 5.89078500 -1.11810500 -0.07073900

H -3.91156900 -2.26412500 2.86436700

H -3.99796500 -3.43889300 1.54065900

H -5.57416400 -1.78817500 0.67029200

H -4.73412800 -0.47292000 1.51657000

H -3.81149300 -0.02752300 -0.59140800

H 3.60385900 -2.99748700 -0.90578000

H 3.68212400 -1.04277300 2.89104100

H -3.07992400 -2.17321300 -2.03614600

H -4.45722800 -2.92425300 -1.19884800

H -4.43392700 -0.39109800 -2.88786500

H -6.93570700 -0.38351400 -2.65461900

H -6.14782500 -0.13393800 -1.09415000

H -6.82758000 -1.72038200 -1.50261800

H -5.82892400 -1.71188000 -4.48609100

H -4.26832800 -2.50041200 -4.20181300

H -5.73163600 -3.11609600 -3.41449600

H -0.02373000 -0.53266900 2.97295700

H 0.67237900 -2.51347200 4.23843500

H 0.60145400 -1.08440500 5.29227200

H 2.16779200 -1.69929900 4.74031100

H 1.48209700 1.45039400 2.66592400

H 1.03540700 1.20853300 4.35102400

H 2.64290400 0.72631200 3.80111100

H 0.12721900 -2.42399700 -1.60615300

H 2.28977900 -2.57155400 -2.84600300

H 1.10882000 -3.81805000 -3.28596200

H 2.48223300 -4.24132400 -2.26296000

H -0.43578200 -4.15611100 0.13226900

H -0.40551200 -4.83629800 -1.50859700

H 0.95425500 -5.10962800 -0.40253800

H 5.66441500 -1.73518500 1.97067600

H 5.42607600 -4.19014400 1.50611700

H 6.93466100 -3.52166500 0.85315700

H 5.58243600 -3.86704300 -0.22703800

H 5.57092700 -0.08942700 0.11593800

H 6.98482800 -1.14498000 -0.07373200

H 5.54033500 -1.39333800 -1.07139300

C -0.78499800 -0.17600700 -1.23436700

O -1.61771900 -0.26134200 -2.24702400

H -2.23975100 0.92458800 -2.20152000

C 0.18777300 3.43690800 0.30840500

O 0.15273200 3.17058200 1.49940600

C 1.14734600 4.51165600 -0.23967500

C 0.44614700 5.85659000 0.02457400

C 2.44859900 4.45630000 0.56457100

C 1.45613600 4.37264600 -1.73570400

H -0.50741800 5.91522300 -0.51004300

H 0.25192000 5.97948900 1.09332400

H 1.08215500 6.68080200 -0.31389200

H 3.02032000 3.55780400 0.31893300

H 3.06660600 5.32693100 0.32504500

H 2.23369500 4.45149700 1.63430300

H 2.25118400 5.07343200 -2.00657800

H 1.79991400 3.36364000 -1.98846200

H 0.59219200 4.62524700 -2.36212500

N -0.68203000 2.91708400 -0.61737300

H -0.44161800 3.10883900 -1.57937900

C -1.83033300 2.12368500 -0.49346600

C -2.60735700 2.02211600 -1.69790500

H -2.39549800 2.78221400 -2.45271600

H -3.67973100 1.89782400 -1.54918100

C 0.59274400 0.27780700 -1.47561000

C 1.08059800 0.26387800 -2.79076400

C 1.44109800 0.75254300 -0.46361400

C 2.38809200 0.64093600 -3.07787700

H 0.41195600 -0.07012700 -3.57772500

C 2.76470200 1.08141700 -0.72663000

H 1.06647500 0.85455300 0.55034700

C 3.22882500 1.02831300 -2.03780100

H 2.76277900 0.62306200 -4.09577200

H 3.42354700 1.39136900 0.07775500

C -2.46198200 1.88890000 0.83595900

C -1.81259400 1.24439300 1.89864500

C -3.78635900 2.30365600 1.04103100

C -2.45890100 1.00739900 3.10376500

H -0.78385700 0.93425400 1.76720300

C -4.43428300 2.08389500 2.25548800

H -4.30623200 2.83750900 0.25214000

C -3.77624200 1.42780800 3.29132900

H -1.92700200 0.49914500 3.90378900

H -5.45261100 2.43547000 2.38983200

H -4.27866600 1.25238300 4.23740600

Cl 4.86935600 1.51773300 -2.39240500

c-M3R

C -3.77879600 -2.68157500 1.61795000

C -4.64287200 -1.59110900 0.93128800

C -3.78876500 -1.08517500 -0.25502100

N -2.44906400 -1.22951000 0.33715800

C -2.40357200 -2.18788100 1.29884600

C -1.19972400 -0.79373200 0.09717500

N -0.43820500 -1.57939500 0.88345700

N -1.18277600 -2.44608300 1.65776900

C 0.99223000 -1.73696700 0.90612500

C 1.60914400 -2.31138800 -0.22127600

C 2.99391300 -2.42596400 -0.18184200

C 3.74319300 -2.02704500 0.93111300

C 3.07703800 -1.50482700 2.03309500

C 1.68844900 -1.34644500 2.04948800

C -3.90511700 -1.93902800 -1.52919700

C -4.71858700 -1.23957000 -2.61981000

C -6.13436000 -0.89350300 -2.15690600

C -4.75713700 -2.10208500 -3.87940000

C 1.00048300 -0.79906700 3.28851000

C 1.12114600 -1.80876200 4.43687700

C 1.54105900 0.57600700 3.69942100

C 0.79680700 -2.82665400 -1.40309500

C 1.63588900 -3.04584700 -2.66184200

C 0.08976600 -4.13809100 -1.02321500

C 5.25268500 -2.16672200 0.93017900

C 5.68701300 -3.61816100 0.70147200

C 5.88157800 -1.23444800 -0.11008700

H -3.95294700 -2.75877300 2.69152900

H -3.93016300 -3.66983600 1.17261400

H -5.60890400 -1.96872100 0.59216900

H -4.80558300 -0.76647100 1.63219600

H -3.96917300 -0.03388800 -0.46946200

H 3.51147900 -2.83848200 -1.04195100

H 3.65196400 -1.21111400 2.90832900

H -2.89915900 -2.10807300 -1.91817500

H -4.36393000 -2.90885300 -1.28182200

H -4.16979600 -0.31735000 -2.85222900

H -6.69004700 -0.37987700 -2.94693700

H -6.13753500 -0.24075800 -1.27633700

H -6.68845300 -1.80524000 -1.90041300

H -5.29704100 -1.59779600 -4.68621900

H -3.74526700 -2.32070100 -4.23168900

H -5.26372400 -3.05431200 -3.68227400

H -0.06552400 -0.69313300 3.07058600

H 0.69186500 -2.77185000 4.14871500

H 0.59323000 -1.44168000 5.32247100

H 2.16961900 -1.96672600 4.71043200

H 1.40669600 1.33293500 2.91954800

H 1.01314800 0.92844900 4.59145100

H 2.60587600 0.52356700 3.94845200

H 0.02943200 -2.08557700 -1.66140600

H 2.21697000 -2.15623000 -2.91722200

H 0.97478800 -3.27508600 -3.50182000

H 2.32312700 -3.89106400 -2.54449000

H -0.55089000 -4.02426000 -0.14543900

H -0.52501600 -4.48747100 -1.85836900

H 0.83055500 -4.91215500 -0.79639400

H 5.60842600 -1.86060600 1.92210200

H 5.23210100 -4.28885800 1.43510700

H 6.77467600 -3.70701600 0.77832000

H 5.39653300 -3.96208200 -0.29635100

H 5.62433400 -0.19049500 0.08697600

H 6.97185900 -1.32785400 -0.10602800

H 5.52417900 -1.47417700 -1.11707800

C -0.95969500 0.28630600 -1.00689400

O -1.62061600 -0.19945800 -2.04268100

H -2.04070000 2.09808200 -2.58138500

C 0.21473800 3.43908700 0.35704500

O 0.28279300 3.12321500 1.53832000

C 1.07500800 4.60397200 -0.18375900

C 0.29076300 5.89201200 0.11390100

C 2.39350400 4.61292700 0.59453700

C 1.37683200 4.50594200 -1.68482600

H -0.66865700 5.89687500 -0.41346900

H 0.09563100 5.97775600 1.18629200

H 0.86622700 6.76604300 -0.20820100

H 2.98889200 3.72735400 0.35250700

H 2.97585600 5.50019000 0.32755700

H 2.20277100 4.61611100 1.66834900

H 2.11436800 5.26709900 -1.95626300

H 1.79148000 3.52603800 -1.94898400

H 0.49171900 4.69690800 -2.30335300

N -0.62447800 2.88087600 -0.55246100

H -0.45705600 3.17348800 -1.50329400

C -1.61517800 1.79206200 -0.49597800

C -2.57598800 2.16601800 -1.63168000

H -2.94600600 3.18393300 -1.48039700

H -3.41095300 1.47340700 -1.71605700

C 0.54365700 0.51098700 -1.28834800

C 0.94547400 0.38397700 -2.61434900

C 1.49288100 0.88487900 -0.33417200

C 2.26872000 0.61544300 -2.99164300

H 0.18670700 0.08869800 -3.33304400

C 2.82237800 1.09086100 -0.68225200

H 1.20231700 1.03375800 0.70167600

C 3.19639000 0.96052100 -2.01843400

H 2.57981600 0.52586400 -4.02719200

H 3.55627400 1.36181800 0.06980700

C -2.32680900 1.73627400 0.85961200

C -1.67216700 1.26247600 2.00505600

C -3.67384400 2.08455700 0.99275000

C -2.33413800 1.10990200 3.21481700

H -0.61748100 1.02607800 1.94791700

C -4.34538300 1.94780200 2.21024000

H -4.22647100 2.46578200 0.14102600

C -3.68275100 1.45040700 3.32476100

H -1.78931900 0.73766800 4.07831000

H -5.39096200 2.23307300 2.27732000

H -4.20269300 1.33879100 4.27107100

Cl 4.85174400 1.28090000 -2.48335600

c-TS4R

C -3.71076800 -3.04221500 1.25155900

C -4.57418500 -1.85551000 0.74477400

C -3.71694300 -1.15355400 -0.33674900

N -2.37380000 -1.41504100 0.19894600

C -2.33471600 -2.50772500 1.01641900

C -1.13212300 -0.90878200 0.04123500

N -0.39055600 -1.79404100 0.73757600

N -1.11852300 -2.79010700 1.36966000

C 1.04305300 -1.86970700 0.77823800

C 1.72409000 -2.24493000 -0.39517300

C 3.11176100 -2.31474500 -0.32747000

C 3.81493200 -2.03015700 0.84800700

C 3.09658300 -1.66361300 1.98008700

C 1.70113400 -1.57798300 1.97342200

C -3.86669000 -1.74974200 -1.74432600

C -4.81252500 -0.93691500 -2.63307800

C -6.20417600 -0.77166100 -2.01985200

C -4.90898500 -1.57529300 -4.01714500

C 0.95640600 -1.20283500 3.24129100

C 1.06853300 -2.33089700 4.27341400

C 1.43985000 0.13387500 3.81537100

C 0.97185300 -2.56833600 -1.67929600

C 1.85955000 -2.53558500 -2.92308400

C 0.27737900 -3.93406400 -1.56663600

C 5.32825500 -2.12370100 0.88299500

C 5.80379100 -3.54854900 0.57899800

C 5.97257900 -1.11803700 -0.07664200

H -3.88905300 -3.28461000 2.29966500

H -3.87031400 -3.94889900 0.65921600

H -5.53764800 -2.17992700 0.34864200

H -4.74613400 -1.15634800 1.56893500

H -3.88537900 -0.07647900 -0.35916700

H 3.66922500 -2.58745300 -1.21818000

H 3.63391500 -1.44307900 2.89971200

H -2.88174900 -1.76367900 -2.21849000

H -4.22476700 -2.78824400 -1.66692800

H -4.34836900 0.05235500 -2.74040400

H -6.85399300 -0.19360200 -2.68367700

H -6.17425500 -0.25187100 -1.05557700

H -6.67695200 -1.74922300 -1.86207200

H -5.53312200 -0.97455500 -4.68529500

H -3.91997600 -1.67505900 -4.47305100

H -5.35571400 -2.57447700 -3.95121800

H -0.10270100 -1.10370500 2.99006000

H 0.67207200 -3.26437200 3.86592400

H 0.50553400 -2.07665600 5.17707400

H 2.11235000 -2.49533500 4.56155600

H 1.34802500 0.95262900 3.09427300

H 0.84743000 0.39831100 4.69752700

H 2.48598100 0.07150600 4.13297300

H 0.20001200 -1.80130800 -1.81731000

H 2.42319700 -1.60089100 -2.98936200

H 1.23651300 -2.62202000 -3.81758200

H 2.56650400 -3.37241200 -2.93247400

H -0.39945100 -3.98158100 -0.71019900

H -0.29581200 -4.14258800 -2.47530300

H 1.02519900 -4.72491900 -1.44563000

H 5.64559400 -1.87217000 1.90286500

H 5.35305300 -4.27045300 1.26494800

H 6.89204300 -3.61742300 0.66874700

H 5.53447200 -3.83934200 -0.44156800

H 5.67561500 -0.09270600 0.16110200

H 7.06385100 -1.17910100 -0.02218600

H 5.67583600 -1.31654000 -1.11171300

C -1.12639700 0.76573100 -1.18656600

O -1.87414400 0.38684800 -2.10379900

H -2.29392600 2.88222300 -2.16916500

C 0.25674500 3.37143900 0.75506500

O 0.41039100 2.80652800 1.82819300

C 1.15790200 4.56635500 0.36605200

C 0.47213500 5.83428600 0.89874500

C 2.50590500 4.37523900 1.06574900

C 1.38908800 4.68533900 -1.14724000

H -0.50770200 5.97900600 0.43233900

H 0.33072100 5.76491900 1.98091400

H 1.08867100 6.71302100 0.68322500

H 3.01820800 3.48995700 0.67649800

H 3.14183200 5.24805300 0.88891000

H 2.36426900 4.24230400 2.13881800

H 2.17410400 5.42355300 -1.33572500

H 1.70860500 3.73148500 -1.58245000

H 0.49996600 5.03843000 -1.68343000

N -0.70769100 3.07261400 -0.15658400

H -0.62134200 3.58888500 -1.01899900

C -1.71993700 2.00575900 -0.27510600

C -2.76369500 2.64632200 -1.21092900

H -3.14754400 3.56527000 -0.76177400

H -3.57987500 1.95935700 -1.42988400

C 0.36659900 0.87682600 -1.48632500

C 0.72847400 0.97104400 -2.82869300

C 1.36241800 0.92551700 -0.51079700

C 2.06412900 1.11544900 -3.20094000

H -0.05695100 0.91343600 -3.57581600

C 2.70104300 1.04676300 -0.86254400

H 1.10050700 0.86685600 0.54145200

C 3.03802500 1.14531900 -2.21052800

H 2.35101000 1.18806200 -4.24444700

H 3.47413400 1.05197300 -0.10102000

C -2.35981800 1.63053100 1.07145600

C -1.64246300 0.97679100 2.08268200

C -3.71226000 1.89226800 1.31889700

C -2.24748100 0.59342500 3.27284500

H -0.59093500 0.77362500 1.94025800

C -4.32672500 1.51114900 2.51305700

H -4.32100500 2.39510800 0.57741500

C -3.59784900 0.85587500 3.49628400

H -1.65522400 0.09538600 4.03533000

H -5.37814600 1.73433500 2.66611700

H -4.06989900 0.55916000 4.42778900

Cl 4.71862800 1.30095600 -2.66527600

t-TS3S-b

C 1.84893400 -4.10999700 0.67260000

C 3.14172500 -3.25767600 0.79472600

C 2.68468200 -1.80888200 1.09657300

N 1.35035300 -1.81580300 0.46873400

C 0.81953600 -3.06731300 0.37887700

C 0.36542800 -0.91695400 0.22106000

N -0.74057200 -1.69100200 0.06179100

N -0.45241000 -3.04433300 0.13592500

C -2.12030900 -1.29949200 0.16527900

C -2.54937800 -0.63087700 1.32986800

C -3.87627300 -0.21556600 1.36137500

C -4.76512500 -0.46722800 0.31174100

C -4.30606300 -1.17700200 -0.79094300

C -2.97997600 -1.60377900 -0.89216600

C 2.51934900 -1.55192100 2.59902500

C 3.83753900 -1.25753300 3.32292500

C 3.65640500 -1.43218800 4.83058300

C 4.32487200 0.15532800 3.00249300

C -2.51980000 -2.36605700 -2.12138800

C -3.12320300 -3.77534700 -2.12636100

C -2.84663200 -1.62157600 -3.42078000

C -1.62954700 -0.40174700 2.52521200

C -2.16768800 0.62732600 3.51893400

C -1.34669900 -1.73270400 3.23870400

C -6.18877800 0.04918800 0.37229800

C -6.92534400 -0.44515000 1.62131600

C -6.20214900 1.58045600 0.29729400

H 1.91081300 -4.84773500 -0.12924600

H 1.59694600 -4.63302000 1.59944600

H 3.80240800 -3.62671500 1.58155500

H 3.68195200 -3.28264600 -0.15219300

H 3.31035100 -1.05551300 0.61385600

H -4.23536500 0.33153500 2.22655200

H -4.99540500 -1.39494800 -1.60376900

H 1.84330100 -0.70023700 2.73286200

H 2.03526300 -2.43601000 3.04071900

H 4.59861800 -1.97658300 2.98437200

H 4.57777000 -1.19318900 5.36907500

H 3.36866700 -2.45708400 5.08383700

H 2.87117100 -0.76097500 5.19660400

H 5.29777700 0.35177300 3.46241100

H 4.42277700 0.32289900 1.92664200

H 3.60984400 0.89345800 3.38228300

H -1.43585700 -2.48160400 -2.06066600

H -2.83201700 -4.31872400 -1.22436800

H -2.77598000 -4.33648500 -2.99982100

H -4.21667100 -3.72987800 -2.16651800

H -2.45251300 -0.59950200 -3.41749100

H -2.41009100 -2.14814300 -4.27521300

H -3.92655100 -1.56065200 -3.58740100

H -0.67685000 -0.00065400 2.16540800

H -2.39972100 1.57420300 3.02248000

H -1.41077000 0.81955100 4.28397400

H -3.06652400 0.26650200 4.02990700

H -0.91643500 -2.47764700 2.56302400

H -0.65100700 -1.57580800 4.06914900

H -2.27561800 -2.14806800 3.64278900

H -6.71542600 -0.34103800 -0.50771600

H -6.90813300 -1.53615200 1.68762700

H -7.96818500 -0.11565300 1.60316500

H -6.46842000 -0.04359500 2.53142700

H -5.72474700 1.93781400 -0.61894000

H -7.22734500 1.96171500 0.32465100

H -5.65550700 2.01557300 1.14079000

C 0.61728900 0.50702600 0.08317200

O 1.60657200 0.88131700 0.94784700

C 3.42369700 2.52598500 -1.43505000

O 3.24596700 3.10249200 -2.49953700

C 4.42484100 3.09807600 -0.40971500

C 5.57412100 2.09661900 -0.22967300

C 4.97936600 4.41762900 -0.94790800

C 3.73006400 3.35309300 0.93550500

H 5.19624800 1.12377800 0.09381400

H 6.11188600 1.95484800 -1.17306600

H 6.28589600 2.46505600 0.51761500

H 4.18019000 5.15104300 -1.08009100

H 5.71857400 4.82271800 -0.24885700

H 5.45180100 4.27152000 -1.92139400

H 4.41956900 3.87187400 1.61076100

H 2.84592500 3.98618100 0.80329200

H 3.41435600 2.43098800 1.42737700

N 2.87720400 1.30716600 -1.08724300

C 1.82014000 0.76270400 -1.75130600

C 0.97153200 1.60513100 -2.67736000

H 1.56853400 1.82887100 -3.56434100

H 0.70178100 2.55610200 -2.21629500

C -0.51865900 1.45799900 -0.03190100

C -0.55570400 2.57792900 0.80612600

C -1.54248700 1.31032000 -0.97722800

C -1.59961500 3.49649900 0.73137800

H 0.24426100 2.71147600 1.52632100

C -2.59456600 2.20994000 -1.05968900

H -1.52393100 0.46871000 -1.66064400

C -2.61719900 3.29959300 -0.19393200

H -1.62562100 4.36062400 1.38614800

H -3.38207600 2.07395600 -1.79326600

C 2.00726300 -0.65642100 -2.20656100

C 3.27351500 -1.24640000 -2.16841400

C 0.94095200 -1.41565400 -2.70849100

C 3.46334300 -2.55803200 -2.60207000

H 4.10655300 -0.64905600 -1.81024700

C 1.12024100 -2.73247800 -3.11154900

H -0.05072800 -0.97529700 -2.76577200

C 2.38754800 -3.31468700 -3.05765000

H 4.46099600 -2.98867600 -2.58606700

H 0.27146300 -3.30421700 -3.47786300

H 2.53514800 -4.33773700 -3.38919400

Cl -3.92787800 4.45146200 -0.28949000

H 0.06326400 1.08706400 -2.98528100

H 2.39187600 1.14682400 0.28460400

t-M3S-b

C 1.72591400 -4.08835500 0.91566700

C 3.03426400 -3.25488400 0.83321000

C 2.61949500 -1.79512400 1.13304400

N 1.25110400 -1.80681700 0.58267200

C 0.69923200 -3.04875500 0.60007000

C 0.29662600 -0.91579400 0.26739300

N -0.82374200 -1.66025800 0.19585800

N -0.58001200 -3.00504200 0.38040700

C -2.20219500 -1.24185100 0.18904300

C -2.68255200 -0.53007300 1.30441500

C -4.01158600 -0.12345700 1.25579900

C -4.84762100 -0.42908500 0.17626900

C -4.33162800 -1.17388400 -0.87761800

C -3.00031000 -1.59589700 -0.89849800

C 2.54888400 -1.50040200 2.63575500

C 3.91321700 -1.12852000 3.22524200

C 3.87605800 -1.22355600 4.74980200

C 4.30401700 0.28059900 2.78307000

C -2.47714300 -2.41649900 -2.06379600

C -3.05155000 -3.83709700 -2.00765200

C -2.77289600 -1.75743400 -3.41548700

C -1.81002300 -0.25414000 2.52403100

C -2.38152400 0.82662400 3.44145600

C -1.59048300 -1.54917800 3.32275800

C -6.28085100 0.06350200 0.14973900

C -7.06159800 -0.37017600 1.39435400

C -6.31516000 1.58643200 -0.01704600

H 1.70160900 -4.91087500 0.19955800

H 1.54996200 -4.49979800 1.91373400

H 3.79097500 -3.60186300 1.53918600

H 3.43800200 -3.31850200 -0.17965700

H 3.21450000 -1.05363200 0.59939800

H -4.41402600 0.45450200 2.08121100

H -4.98051300 -1.43025800 -1.71183300

H 1.85584400 -0.66564900 2.78085900

H 2.14206000 -2.38679900 3.14500000

H 4.67014200 -1.83735400 2.85603800

H 4.83368600 -0.92528900 5.18630900

H 3.65114000 -2.24116900 5.08356900

H 3.10339200 -0.55795300 5.15024700

H 5.30785900 0.54667200 3.12855900

H 4.27914100 0.38358800 1.69437600

H 3.58991400 1.00692600 3.18627200

H -1.39243000 -2.50357600 -1.96187100

H -2.78722400 -4.31945800 -1.06364700

H -2.65721000 -4.44028900 -2.83152700

H -4.14308800 -3.81720900 -2.09365600

H -2.39513000 -0.73069100 -3.46238400

H -2.30221900 -2.32714900 -4.22269600

H -3.84752700 -1.72609800 -3.61964600

H -0.83084700 0.11175300 2.19236700

H -2.60555400 1.74260400 2.88814900

H -1.64865400 1.06544600 4.21658200

H -3.29400800 0.48766900 3.94399400

H -1.14644100 -2.34245600 2.71561800

H -0.93020800 -1.35586900 4.17382900

H -2.54540900 -1.92224200 3.70730100

H -6.76377500 -0.38773100 -0.72625700

H -7.03097000 -1.45503300 1.52518000

H -8.10750400 -0.06039800 1.31322300

H -6.65007600 0.09152000 2.29749700

H -5.79959800 1.89679400 -0.92982000

H -7.34630900 1.94976300 -0.06156700

H -5.81606700 2.08102600 0.82282800

C 0.65327100 0.58750800 0.11472400

O 1.33841800 0.87838700 1.22186400

C 3.84762200 1.96214100 -1.34252800

O 4.07263600 1.76153500 -2.53206300

C 4.78684300 2.87740400 -0.53093000

C 6.06461500 2.07372700 -0.24682100

C 5.13514500 4.06973200 -1.43031400

C 4.16660400 3.38847800 0.77154000

H 5.85858000 1.20815000 0.39137800

H 6.49771000 1.71712400 -1.18547300

H 6.80177400 2.70232300 0.26390800

H 4.24601500 4.67270600 -1.64044400

H 5.87338900 4.70825100 -0.93443300

H 5.53873500 3.71878900 -2.38091500

H 4.84887800 4.10233900 1.24418500

H 3.21744400 3.90004100 0.58205400

H 3.98032200 2.58852400 1.49276100

N 2.82866900 1.37098300 -0.66882100

C 1.65964600 0.76395700 -1.27835700

C 1.09403900 1.73842700 -2.32731400

H 1.87390500 1.91437600 -3.06952000

H 0.82078900 2.68531000 -1.85553200

C -0.59573100 1.47687700 -0.06041000

C -0.72622200 2.54071300 0.82861900

C -1.56413300 1.32410900 -1.05591800

C -1.79206400 3.43350000 0.73519900

H 0.03251700 2.64181800 1.59755700

C -2.64176500 2.19392100 -1.16182300

H -1.49394600 0.50556100 -1.76561800

C -2.74157400 3.25036900 -0.26078000

H -1.88542600 4.26508700 1.42541500

H -3.38977200 2.06112700 -1.93613900

C 1.96184400 -0.56218000 -1.96859300

C 3.24405500 -1.11226200 -2.01158400

C 0.92694300 -1.27086200 -2.59257400

C 3.47436600 -2.34388700 -2.62443400

H 4.07266400 -0.56209300 -1.57961000

C 1.14550100 -2.51195000 -3.18051100

H -0.07628500 -0.85146800 -2.61040000

C 2.42617200 -3.06070500 -3.19318400

H 4.48484600 -2.74123700 -2.65813600

H 0.31633200 -3.04274300 -3.64118600

H 2.60659300 -4.02264300 -3.66210800

Cl -4.06706200 4.38169200 -0.40161800

H 0.21740100 1.34304600 -2.84024900

H 2.62903500 1.53240100 0.33430600

t-TS4S-b

C 1.36874200 -4.21446800 1.16463400

C 2.70725500 -3.42902000 1.10795300

C 2.33307300 -1.93991600 1.30457700

N 0.98284200 -1.92927000 0.72802300

C 0.39516100 -3.16238800 0.74082500

C 0.08921500 -0.98937300 0.34833500

N -1.03082300 -1.72975400 0.20895300

N -0.86176900 -3.08874900 0.43084900

C -2.37284800 -1.23968800 0.05963300

C -2.93668600 -0.50099000 1.11789400

C -4.24376500 -0.05393500 0.95126100

C -4.98497200 -0.32706100 -0.20284100

C -4.38406300 -1.05975500 -1.21973100

C -3.07133200 -1.52778200 -1.11337500

C 2.27713100 -1.52104500 2.77607100

C 3.66216000 -1.24749600 3.37390600

C 3.56351500 -1.12738600 4.89454600

C 4.27839700 0.02169900 2.78136500

C -2.45148800 -2.33465500 -2.23770800

C -3.04263200 -3.74865700 -2.27024500

C -2.60482600 -1.63811200 -3.59372600

C -2.15154500 -0.20182500 2.38849000

C -2.76326300 0.92016500 3.22686600

C -1.99534200 -1.46824800 3.24397200

C -6.40981400 0.17300200 -0.34135600

C -7.30670600 -0.36907600 0.77685700

C -6.45511200 1.70366100 -0.38269800

H 1.35714100 -5.08162800 0.50312800

H 1.13139500 -4.55751400 2.17639200

H 3.41912600 -3.76698500 1.86346900

H 3.15771200 -3.55814500 0.12088100

H 2.97191000 -1.26882900 0.73150700

H -4.70317600 0.53356900 1.73986200

H -4.95323800 -1.27917900 -2.12034700

H 1.66838600 -0.61349700 2.85290400

H 1.77120000 -2.31534500 3.34493100

H 4.32266700 -2.09629100 3.14138900

H 4.54182700 -0.92158900 5.33846400

H 3.17176300 -2.04512000 5.34343800

H 2.89185600 -0.30552500 5.16587800

H 5.27974100 0.19596100 3.18779500

H 4.36333400 -0.03021700 1.69070000

H 3.64970400 0.88752600 3.01518600

H -1.38386200 -2.43247800 -2.02601900

H -2.87295700 -4.25377700 -1.31649200

H -2.57827600 -4.33880700 -3.06689400

H -4.12108800 -3.71310400 -2.45799800

H -2.22877100 -0.60992400 -3.56555400

H -2.05243600 -2.18239000 -4.36622300

H -3.65225000 -1.60040000 -3.90918700

H -1.15133100 0.13384900 2.08919800

H -2.94556900 1.81632600 2.62711100

H -2.07711200 1.18546500 4.03580200

H -3.70784300 0.60847900 3.68616100

H -1.51873600 -2.28347600 2.69396400

H -1.39344000 -1.25002500 4.13166300

H -2.97744600 -1.82074500 3.57626800

H -6.79550700 -0.20560700 -1.29631400

H -7.27653200 -1.46109500 0.81427000

H -8.34313000 -0.05521000 0.62132100

H -6.98710700 0.01088100 1.75251400

H -5.85973700 2.09798500 -1.21098300

H -7.48391500 2.05688200 -0.50193400

H -6.05598500 2.13199000 0.54236000

C 0.93741100 0.98614500 0.20273000

O 1.58588800 1.05856500 1.26001100

C 4.16869300 1.81215700 -1.47810400

O 4.20876400 1.60090000 -2.68316400

C 5.38275400 2.43666500 -0.76093000

C 6.42142400 1.31424700 -0.61021700

C 5.94026700 3.52816700 -1.68069600

C 5.05408400 3.03765400 0.60838800

H 6.05640500 0.52524300 0.05744000

H 6.64207500 0.87222000 -1.58575900

H 7.34921100 1.71155700 -0.18571500

H 5.22775200 4.35283000 -1.78227300

H 6.87115500 3.92766500 -1.26604200

H 6.13072900 3.12095000 -2.67452800

H 5.94001400 3.54328100 1.00496300

H 4.24878200 3.77562500 0.53800200

H 4.76528300 2.27781500 1.34229700

N 3.11916600 1.45982800 -0.69171800

C 1.83586300 0.93797600 -1.16720500

C 1.28583400 1.91437500 -2.22485100

H 2.02324900 1.99959900 -3.02264600

H 1.11936900 2.89579100 -1.77349100

C -0.36077200 1.77654700 0.12794400

C -0.49059200 2.83336500 1.02842500

C -1.38615500 1.54691200 -0.78948500

C -1.61171500 3.65873700 1.00774600

H 0.30251500 2.99477800 1.75047300

C -2.51797800 2.35107500 -0.81961700

H -1.32503000 0.70451100 -1.47100000

C -2.61482700 3.40938800 0.07959100

H -1.71144900 4.48337100 1.70490500

H -3.32022700 2.15167700 -1.52198000

C 2.01193400 -0.45123700 -1.79395700

C 3.22813200 -1.13384000 -1.73158900

C 0.95560800 -1.07034900 -2.47265800

C 3.37549000 -2.40251900 -2.28931500

H 4.07825000 -0.66898600 -1.24546500

C 1.09445000 -2.34032900 -3.02428500

H -0.00283000 -0.56635600 -2.56316800

C 2.30600900 -3.01965500 -2.92821500

H 4.33857300 -2.90143200 -2.23051000

H 0.25370100 -2.79614800 -3.53968700

H 2.41896300 -4.00648400 -3.36541200

Cl -4.02324400 4.44022100 0.04724400

H 0.34420100 1.57405200 -2.65385400

H 3.14600400 1.61378200 0.30894500

t-TS3R-b

C -2.84962800 -3.19675100 1.81545300

C -3.92439700 -2.39675700 1.03700200

C -3.16586600 -1.67779600 -0.09930600

N -1.87077400 -1.44023800 0.57750900

C -1.62193500 -2.39433800 1.52534300

C -0.70220500 -0.80059700 0.31156800

N 0.20797000 -1.44915600 1.08737400

N -0.37447500 -2.43567500 1.86794200

C 1.63868900 -1.45102800 0.95669000

C 2.20114100 -1.86948400 -0.26684900

C 3.58453800 -1.79866500 -0.38152200

C 4.39903300 -1.36040600 0.66755500

C 3.80082300 -1.01607500 1.87300800

C 2.41402900 -1.05083600 2.04603100

C -2.88646000 -2.53052200 -1.34108000

C -4.11534000 -2.97033100 -2.14294900

C -3.65966000 -3.80469100 -3.34131500

C -4.94908300 -1.77691900 -2.60894500

C 1.80757000 -0.67248700 3.38497000

C 2.08646100 -1.77238900 4.41645000

C 2.30907200 0.68851200 3.88093700

C 1.34593500 -2.41628300 -1.40362400

C 2.07080600 -2.45015800 -2.74870000

C 0.82446400 -3.81722800 -1.04839300

C 5.90045300 -1.26322600 0.48322400

C 6.51959600 -2.63223000 0.18201900

C 6.25118200 -0.24945700 -0.61141200

H -3.05725200 -3.26121700 2.88415400

H -2.72037100 -4.21329000 1.43109600

H -4.72396600 -3.03225500 0.65239100

H -4.35293500 -1.63030100 1.68840500

H -3.62747300 -0.72964700 -0.36539000

H 4.04979600 -2.08188200 -1.31989900

H 4.42889100 -0.69944900 2.70260700

H -2.22822400 -1.93887700 -1.99113900

H -2.32049800 -3.42116500 -1.03006500

H -4.74437400 -3.60871400 -1.50733000

H -4.51716600 -4.17146600 -3.91240400

H -3.06504200 -4.66812400 -3.02717600

H -3.04314000 -3.19736600 -4.01325900

H -5.75008700 -2.10132300 -3.27958400

H -5.41497900 -1.24458200 -1.77458200

H -4.31925300 -1.06339800 -3.15355400

H 0.72354800 -0.61022200 3.26380900

H 1.66621700 -2.72409100 4.08228900

H 1.63976100 -1.51245000 5.38091700

H 3.16392300 -1.90202100 4.56392600

H 2.17099200 1.47365000 3.12930500

H 1.76372700 0.98165500 4.78295500

H 3.37332500 0.65503900 4.13395300

H 0.48266900 -1.75523200 -1.53649200

H 2.49727000 -1.47361800 -2.99514000

H 1.36299100 -2.72422600 -3.53583700

H 2.87220200 -3.19654700 -2.75529800

H 0.26783300 -3.82258500 -0.10681800

H 0.16995100 -4.18962100 -1.84343500

H 1.66274100 -4.51355100 -0.94234500

H 6.32176300 -0.90238600 1.42998500

H 6.27977400 -3.35658600 0.96482300

H 7.60788200 -2.55122100 0.10576500

H 6.14814200 -3.02735000 -0.76899900

H 5.83967100 0.73824000 -0.38720900

H 7.33611500 -0.15577500 -0.71790000

H 5.84367500 -0.56063600 -1.57910100

C -0.62167700 0.40727300 -0.47548200

O -1.65883000 0.46051600 -1.38456000

C -3.76625100 1.33666800 0.97237200

O -3.80541400 0.64590900 1.99315900

C -5.08783900 1.70501600 0.26564700

C -4.91654300 1.84091900 -1.25035900

C -6.15118800 0.65585000 0.59310800

C -5.51345400 3.06315800 0.84884000

H -4.23398900 2.65790400 -1.49218300

H -4.51832500 0.92327900 -1.69931200

H -5.88718200 2.04270500 -1.71663900

H -6.26640900 0.55007400 1.67342400

H -7.11272900 0.94209000 0.15473000

H -5.87828400 -0.32804300 0.19270800

H -6.45631000 3.38987300 0.39664200

H -5.65674600 2.98742900 1.93106100

H -4.74833400 3.81867800 0.65009600

N -2.67036400 1.92358000 0.39732100

C -1.42154600 1.84996200 0.93625900

C -1.13773000 1.26336800 2.30381200

H -1.61984100 0.30098500 2.45156200

H -1.54564000 1.93481100 3.06863200

C 0.69026200 0.87083600 -1.01213400

C 0.86161700 0.95946100 -2.39662800

C 1.74243500 1.28370200 -0.18716100

C 2.05920800 1.40982900 -2.94561900

H 0.03707400 0.67369500 -3.04095200

C 2.94091600 1.73902400 -0.71573600

H 1.61838700 1.26495900 0.88940500

C 3.09255700 1.78854900 -2.09803000

H 2.19131500 1.47019300 -4.02042600

H 3.74180500 2.07030300 -0.06382300

C -0.57681300 3.03884100 0.56922400

C -0.68266000 3.59626800 -0.71241000

C 0.33528800 3.60681400 1.46378300

C 0.13481600 4.64706000 -1.10179100

H -1.39258100 3.16824600 -1.41229900

C 1.14281700 4.67605900 1.08028300

H 0.42426100 3.22261400 2.47473000

C 1.05793300 5.18984800 -0.20771600

H 0.05594900 5.04438900 -2.10885500

H 1.84214100 5.10192700 1.79321700

H 1.69781700 6.01132700 -0.51350100

Cl 4.59851000 2.35837300 -2.77795800

H -0.06029500 1.17597000 2.46178500

H -2.29560300 1.14568000 -0.97647300

t-M3R-b

C -2.09842200 -4.00216700 1.55054000

C -3.34243700 -3.26804300 0.98665900

C -2.80122600 -2.28531900 -0.07563000

N -1.48636800 -1.96454700 0.52657300

C -1.03471100 -2.97728800 1.32031600

C -0.47793200 -1.09443500 0.33402500

N 0.55994000 -1.65646100 0.98101900

N 0.21969500 -2.82837200 1.61988200

C 1.96141000 -1.33262500 0.88677400

C 2.60267800 -1.55874200 -0.34711200

C 3.94880500 -1.22272000 -0.42020700

C 4.65151000 -0.71578400 0.67850300

C 3.98096300 -0.56053200 1.88535800

C 2.62365800 -0.86739600 2.02118200

C -2.54899500 -2.91092400 -1.44881600

C -3.81453900 -3.29940600 -2.22025800

C -3.41378500 -3.86247700 -3.58451900

C -4.75238400 -2.10312900 -2.38950200

C 1.94282400 -0.73934400 3.37193800

C 2.36989000 -1.90196300 4.27774900

C 2.22422400 0.60876800 4.04328800

C 1.87960100 -2.19292800 -1.52961300

C 2.60061200 -1.98644000 -2.86186300

C 1.68193000 -3.69839900 -1.28649200

C 6.11812200 -0.35606100 0.54803300

C 6.96260600 -1.58177800 0.18122400

C 6.31731300 0.77067400 -0.47091000

H -2.19452000 -4.27082300 2.60312600

H -1.86271900 -4.91078900 0.98837800

H -4.07681600 -3.95437100 0.56259200

H -3.82470400 -2.70155100 1.78869300

H -3.38990400 -1.37638300 -0.15288700

H 4.47353300 -1.35608400 -1.36065300

H 4.52940200 -0.19203200 2.74890600

H -1.98704200 -2.16291100 -2.02007400

H -1.90974600 -3.79773700 -1.32052900

H -4.34764500 -4.09020600 -1.67306500

H -4.29055500 -4.20217700 -4.14284500

H -2.72616000 -4.70766500 -3.48184800

H -2.91267300 -3.09058500 -4.17849000

H -5.57375900 -2.34249200 -3.07117500

H -5.19747300 -1.78781000 -1.43977800

H -4.19937500 -1.25045700 -2.80125200

H 0.86253400 -0.82177800 3.22240400

H 2.11056100 -2.86041300 3.82144900

H 1.87332900 -1.82979100 5.25015300

H 3.45182000 -1.88073000 4.44534200

H 1.99418600 1.45038200 3.38215700

H 1.61657800 0.70944200 4.94704400

H 3.27364600 0.69514600 4.34109600

H 0.89246600 -1.72392000 -1.62590000

H 2.83510800 -0.93357300 -3.03425300

H 1.96159900 -2.33442700 -3.67793900

H 3.52916400 -2.56572500 -2.90335400

H 1.14933800 -3.90547900 -0.35546200

H 1.12221800 -4.14216100 -2.11577100

H 2.65551800 -4.19616700 -1.22835400

H 6.45422400 0.00553600 1.52785800

H 6.83099400 -2.38632800 0.90949100

H 8.02343500 -1.31744700 0.14430400

H 6.68078100 -1.96763300 -0.80381000

H 5.73907800 1.65805200 -0.20141800

H 7.37305200 1.05103900 -0.53370100

H 5.99142100 0.45997300 -1.46897600

C -0.73338200 0.26048800 -0.40790200

O -1.56763300 -0.01639200 -1.40955500

C -3.87343800 1.63830800 1.12716400

O -3.69240800 2.31933100 2.12604600

C -5.28570000 1.44851800 0.53781300

C -5.26053200 1.44844600 -0.99508000

C -5.83973300 0.11486600 1.06225500

C -6.16693400 2.59496200 1.03671500

H -4.78754500 2.35847500 -1.37785500

H -4.72333400 0.58851000 -1.40776800

H -6.28408500 1.40634800 -1.38181400

H -5.86838600 0.11555900 2.15622800

H -6.85729100 -0.04431000 0.68945400

H -5.22297700 -0.72995900 0.73730800

H -7.19350800 2.45653000 0.68253800

H -6.16237900 2.63541500 2.12685200

H -5.79678300 3.55568700 0.66855600

N -2.87621800 0.94511700 0.50517800

C -1.46423800 1.23752200 0.72832300

C -1.09040200 0.90801000 2.17472100

H -1.41362600 -0.10660800 2.42785100

H -1.61070300 1.59038300 2.84697800

C 0.59134300 0.90202100 -0.88242200

C 0.79965000 0.94884400 -2.25804600

C 1.53716500 1.50337900 -0.04723800

C 1.92877400 1.56094600 -2.79782700

H 0.03405500 0.51330700 -2.89182200

C 2.67378900 2.11211900 -0.56306600

H 1.39765100 1.51392900 1.02798200

C 2.85997400 2.13239200 -1.94178900

H 2.08218600 1.60422200 -3.87100400

H 3.39744700 2.58237700 0.09379300

C -1.20523200 2.70198500 0.36732200

C -1.54027200 3.13632300 -0.92176300

C -0.65236800 3.62008800 1.25761000

C -1.31597800 4.45112000 -1.30801000

H -1.94285400 2.41295300 -1.62694700

C -0.43337800 4.94206600 0.87257900

H -0.38941700 3.31695800 2.26581600

C -0.76187200 5.36248600 -0.41011800

H -1.56989400 4.76613800 -2.31560300

H -0.00830500 5.64379700 1.58367900

H -0.58859100 6.39118300 -0.71014500

Cl 4.27247700 2.92080100 -2.60915800

H -0.01440200 0.98231100 2.35007400

H -2.97297900 0.61100200 -0.46184900

t-TS4R-b

C -1.84657900 -4.43425800 0.83827400

C -3.10686400 -3.63837400 0.40663800

C -2.59341700 -2.45817600 -0.45399300

N -1.25814300 -2.25995300 0.13371600

C -0.80310400 -3.36498000 0.79789300

C -0.29073300 -1.31903300 0.08528800

N 0.72262900 -1.93796300 0.72470300

N 0.42490200 -3.20824800 1.18576800

C 2.06442900 -1.44537300 0.87831500

C 2.88732200 -1.38665000 -0.26207200

C 4.17035300 -0.87541100 -0.09224700

C 4.64316700 -0.45256000 1.15305600

C 3.80478900 -0.56314200 2.25677000

C 2.50389900 -1.06397900 2.14971200

C -2.44830800 -2.77658000 -1.94159900

C -3.77267400 -2.83407800 -2.71080200

C -3.48821900 -3.16173500 -4.17721300

C -4.54116400 -1.51379400 -2.59633000

C 1.65070500 -1.24912400 3.39256500

C 2.06583000 -2.53900500 4.11347000

C 1.71638100 -0.05049300 4.34387200

C 2.40380500 -1.88155500 -1.61879000

C 3.28732900 -1.43122100 -2.78186800

C 2.29752600 -3.41506700 -1.62682500

C 6.03834700 0.12589100 1.29187500

C 7.10968300 -0.82874100 0.75506700

C 6.12952800 1.49197700 0.60129000

H -1.94379800 -4.88908400 1.82459900

H -1.59596300 -5.22496000 0.12426500

H -3.82141800 -4.25418500 -0.14277400

H -3.60884300 -3.24552000 1.29554100

H -3.17604800 -1.55184700 -0.30616500

H 4.82128800 -0.78894100 -0.95660900

H 4.17556000 -0.25577000 3.23135400

H -1.82420700 -1.98956000 -2.38017600

H -1.91240300 -3.73146900 -2.04806800

H -4.39623600 -3.64077200 -2.29842000

H -4.41704700 -3.26316900 -4.74548000

H -2.92446900 -4.09446400 -4.27638200

H -2.89830500 -2.36057400 -4.63586900

H -5.37924000 -1.48918100 -3.29917500

H -4.95725900 -1.36646000 -1.59411700

H -3.87564300 -0.66962600 -2.81046200

H 0.61091000 -1.36954000 3.07539900

H 1.95238800 -3.40173700 3.45303200

H 1.44861200 -2.69333500 5.00406000

H 3.11244700 -2.47850200 4.43055700

H 1.51277800 0.89185100 3.82670300

H 0.97607100 -0.16858800 5.13999400

H 2.69810000 0.03028900 4.82080400

H 1.40435300 -1.46148500 -1.78528500

H 3.45706600 -0.35270100 -2.77179800

H 2.80271300 -1.68868800 -3.72768200

H 4.25852100 -1.93803100 -2.76129300

H 1.64566300 -3.79381300 -0.83711100

H 1.91231600 -3.75958700 -2.59148500

H 3.28873400 -3.85687900 -1.48051900

H 6.22454700 0.27421300 2.36299800

H 7.04132800 -1.80931400 1.23321600

H 8.10843400 -0.42179300 0.93800500

H 7.00368300 -0.97100800 -0.32505100

H 5.41736600 2.19951600 1.03593300

H 7.13423100 1.91269700 0.70440000

H 5.90796800 1.40206300 -0.46817100

C -0.92912800 0.49365900 -0.65689900

O -1.79781800 0.18114700 -1.50631700

C -3.81453300 1.39490000 1.53277900

O -3.47403100 2.00449300 2.53392400

C -5.29806200 1.17037400 1.18149400

C -5.57881400 1.64735200 -0.25151400

C -5.62617000 -0.32349100 1.31896200

C -6.14864300 1.97370400 2.16511200

H -5.25906000 2.68586700 -0.38478200

H -5.07198800 1.03698600 -1.00742600

H -6.65289400 1.59126400 -0.45576800

H -5.42663700 -0.67143500 2.33754300

H -6.68455500 -0.49768600 1.09871600

H -5.03337100 -0.93318700 0.62967400

H -7.21072000 1.80414900 1.96188200

H -5.92868800 1.68123300 3.19358400

H -5.93767100 3.04212100 2.07653800

N -2.92186200 0.83373400 0.66419400

C -1.49938700 1.17561900 0.67603000

C -0.88978300 0.68116100 1.98830400

H -1.24466900 -0.33408300 2.17961300

H -1.22910300 1.31048900 2.81113300

C 0.38432200 1.05161900 -1.19790900

C 0.48477700 1.15842200 -2.58619700

C 1.44714200 1.50587500 -0.41470600

C 1.61908900 1.69093500 -3.18949500

H -0.35500900 0.82173000 -3.18370900

C 2.59076600 2.03654100 -1.00038400

H 1.41284000 1.44492900 0.66657200

C 2.66580700 2.12362100 -2.38467800

H 1.69594300 1.76937300 -4.26847800

H 3.42022900 2.37212900 -0.38830100

C -1.34563400 2.69232100 0.47083200

C -1.95246000 3.27709800 -0.64617000

C -0.61562000 3.50543300 1.33484500

C -1.83701800 4.64027200 -0.88768500

H -2.50352600 2.64599400 -1.33927000

C -0.49891500 4.87321500 1.09468200

H -0.13449500 3.08123500 2.21005000

C -1.10926200 5.44612800 -0.01479900

H -2.31140700 5.07460900 -1.76225800

H 0.06922800 5.49111000 1.78305000

H -1.01766800 6.51149600 -0.20052900

Cl 4.12159900 2.75035700 -3.12377600

H 0.20089300 0.65817000 1.96110600

H -3.22995800 0.55352400 -0.26276700

P1S

O -0.84182500 -0.58846700 2.78447900

O 0.42332900 2.15080600 1.51282100

N -0.89541200 1.34772300 -0.13199400

H -0.93465600 1.13447300 -1.11762500

C -0.63422600 -0.35248300 1.61711700

C -4.32392400 -2.08131200 -1.44616100

C -3.72720400 -2.54174600 -0.27601700

C -2.55614400 -0.43811700 -0.01751600

C -3.17716100 0.02469700 -1.18175600

H -3.00009300 1.04092600 -1.52458600

C -2.85291700 -1.72627300 0.43645400

H -2.40300400 -2.09331300 1.35430200

C 0.60493700 -0.87262200 0.94399400

C -1.63928600 0.48444900 0.78566500

C 0.15762200 2.09416900 0.32168500

C 1.78801100 -0.85022900 1.68522900

H 1.76088800 -0.45125100 2.69376800

C 0.61508900 -1.37707800 -0.35779500

H -0.30163000 -1.41101800 -0.93971700

C 2.96547600 -1.81132700 -0.16630700

C -2.49607200 1.32521000 1.74206700

H -3.20997300 1.90912400 1.15648200

H -1.85501500 1.98838700 2.32368900

H -3.03721500 0.66607800 2.42204000

C -4.05077700 -0.79246100 -1.89331100

H -4.52285000 -0.41588300 -2.79512300

C 0.13954800 3.42949400 -1.84093900

H -0.58592300 4.12984300 -1.41731200

H -0.40794600 2.68663900 -2.43096700

H 0.77942200 3.98057600 -2.53688300

C 2.97693500 -1.30727900 1.13138100

H 3.90437200 -1.27849000 1.69181900

C 1.00646900 2.79491500 -0.74620600

C 1.79411900 -1.86302300 -0.91431700

H 1.81192000 -2.27014700 -1.91896100

C 1.84059200 3.87446200 -0.05492000

H 2.44470700 3.43966700 0.74264900

H 1.19778800 4.63753000 0.39247400

H 2.49892200 4.35513200 -0.78469200

C 1.93642900 1.73045700 -1.35432900

H 1.37747200 0.98299800 -1.92671900

H 2.49410200 1.20555200 -0.57072900

H 2.65255000 2.20762000 -2.03076600

Cl 4.45235500 -2.38700000 -0.87099700

H -3.94060900 -3.54182100 0.08735600

H -5.00249200 -2.71981900 -2.00210400

P1R

O -0.84181800 0.58787200 2.78457400

O 0.42354800 -2.15071900 1.51270200

N -0.89556900 -1.34796400 -0.13200200

H -0.93499000 -1.13492000 -1.11768300

C -0.63425700 0.35214100 1.61715500

C -4.32326500 2.08199500 -1.44612900

C -3.72676600 2.54197700 -0.27569800

C -2.55605200 0.43808600 -0.01752600

C -3.17693200 -0.02429700 -1.18204000

H -3.00005500 -1.04046300 -1.52512500

C -2.85273200 1.72614500 0.43672500

H -2.40290400 2.09288200 1.35473600

C 0.60482800 0.87246900 0.94404500

C -1.63938300 -0.48466800 0.78565500

C 0.15755200 -2.09429300 0.32161000

C 1.78788900 0.85025400 1.68532000

H 1.76075800 0.45131800 2.69387200

C 0.61498800 1.37690600 -0.35775000

H -0.30171500 1.41084300 -0.93967700

C 2.96532100 1.81147200 -0.16618700

C -2.49616900 -1.32545400 1.74200200

H -3.21003600 -1.90936500 1.15641200

H -3.03732400 -0.66638800 2.42200700

H -1.85509900 -1.98863900 2.32359200

C -4.05024000 0.79319500 -1.89354300

H -4.52217400 0.41696600 -2.79557100

C 1.93670300 -1.73072200 -1.35398000

H 2.49449700 -1.20633700 -0.57013900

H 1.37805200 -0.98284900 -1.92611400

H 2.65268500 -2.20792300 -2.03053300

C 2.97677500 1.30742800 1.13150800

H 3.90418800 1.27878500 1.69199400

C 1.00629300 -2.79505400 -0.74632400

C 1.79398200 1.86300700 -0.91422500

H 1.81176000 2.27012200 -1.91887400

C 1.83995200 -3.87511600 -0.05526600

H 1.19681900 -4.63790700 0.39211000

H 2.44437100 -3.44074800 0.74229300

H 2.49795400 -4.35603000 -0.78516200

C 0.13942600 -3.42897100 -1.84143600

H -0.40778000 -2.68577700 -2.43128600

H -0.58627600 -4.12935300 -1.41825900

H 0.77930000 -3.97988100 -2.53750200

Cl 4.45219200 2.38720500 -0.87086100

H -3.94005000 3.54197400 0.08795000

H -5.00158400 2.72073600 -2.00209600
